# Supplementary material for: Association between maternal education and breast feeding practices in China: a population-based cross-sectional study
Source: BMJ Open. 2019 Aug 28;9(8):e028485. doi: 10.1136/bmjopen-2018-028485 (PMC6720234; doi:10.1136/bmjopen-2018-028485)
Supplement: Supplementary data [file bmjopen-2018-028485supp001.pdf]

Supplementary 1. Infant age at interview by maternal education.

	Maternal Education			
	Primary school and below (N=781)	Middle School (N=3842)	High School/Vocational School (N=1990)	College and above (N=3795)
Mean infant age, day (SD)	186.50(106.82)	182.11(104.48)	178.29(105.82)	173.10(105.52)
Range of infant age, day	0-364	0-364	0-364	0-364
Median	190.00	183.00	181.00	170.00
Distribution of infant age, %				
0~91 days (>0, ≤3 months)	23.30	24.83	25.83	27.27
92~183 days (>3, ≤6 months)	24.46	25.25	25.08	26.03
184~274 days (>6, ≤9 months)	24.33	25.43	26.13	23.90
275~364 days (>9, ≤12 months)	27.91	24.49	22.96	22.79
