# Supplementary material for: Association between maternal education and breast feeding practices in China: a population-based cross-sectional study
Source: BMJ Open. 2019 Aug 28;9(8):e028485. doi: 10.1136/bmjopen-2018-028485 (PMC6720234; doi:10.1136/bmjopen-2018-028485)
Supplement: Author's manuscript [file bmjopen-2018-028485.draft_revisions.pdf]

BMJ Open is committed to open peer review. As part of this commitment we make the peer review history of every article we publish publicly available.

When an article is published we post the peer reviewers' comments and the authors' responses online. We also post the versions of the paper that were used during peer review. These are the versions that the peer review comments apply to.

The versions of the paper that follow are the versions that were submitted during the peer review process. They are not the versions of record or the final published versions. They should not be cited or distributed as the published version of this manuscript.

BMJ Open is an open access journal and the full, final, typeset and author-corrected version of record of the manuscript is available on our site with no access controls, subscription charges or pay-per-view fees (http://bmjopen.bmj.com).

If you have any questions on BMJ Open's open peer review process please email info.bmjopen@bmj.com

BMJ Open

The association between maternal education and breastfeeding practices in China: a population-based cross-sectional study

Journal:	*BMJ Open*
Manuscript ID	bmjopen-2018-028485
Article Type:	Research
Date Submitted by the Author:	10-Dec-2018
Complete List of Authors:	Tang, Kun; Tsinghua University, Research Center for Public Health<br>Wang, Hanyu; Peking University Health Science Centre, Institute for Medical Humanities<br>Tan, Shi Hui; Peking University Health Science Centre, School of Basic Medical Sciences<br>Xin, Tong; Peking University Health Science Centre, Department of Global Health<br>Wang, Yuqi; Peking University Health Science Centre, Institute for Medical Humanities<br>Liu, Yuning; Peking University Health Science Centre, School of Public Health<br>Qu, Xueqi; Peking University Health Science Centre, School of Public Health<br>Gaoshan, Junjian; United Nations Population Fund China Office
Keywords:	breastfeeding, EPIDEMIOLOGY, China, Maternal medicine < OBSTETRICS, SOCIAL MEDICINE

SCHOLARONE™
Manuscripts

The association between maternal education and breastfeeding practices in China: a population-based cross-sectional study

Authors: Kun Tang[1],* Hanyu Wang[2],* Shi Hui Tan[3], Tong Xin[4], Yuqi Wang[5], Yuning Liu[6], Xueqi Qu[7], Junjian Gaoshan[8]

*Co-first authors

1 Research Center for Public Health, Tsinghua University, Beijing, China. E-mail: tangkun@post.harvard.edu

2 Institute for Medical Humanities, Peking University Health Science Center, Beijing, China. E-mail: whybest@pku.edu.cn

3 School of Basic Medical Sciences, Peking University Health Science Center, Beijing, China. E-mail: shi.tan@mail.mcgill.ca

4 Department of Global Health, Peking University Health Science Center, Beijing, China. E-mail: xtong.katrina@gmail.com

5 Institute for Medical Humanities, Peking University Health Science Center, Beijing, China. E-mail: 18811761299@163.com

6 School of Public Health, Peking University Health Science Center, Beijing, China. E-mail: lynpkuhsc@126.com

7 School of Public Health, Peking University Health Science Center, Beijing, China. E-mail: quxueqisnow@163.com

8 United Nations Population Fund China Office. E-mail: j.gaoshan@gmail.com

Corresponding Author: Dr. Kun Tang

Address: Tsinghua University, Haidian District, Beijing 100191, P.R. China

Telephone Number: 86-10-82805951

E-mail Address：tangkun@post.harvard.edu

Key Words: Breastfeeding, maternal education, income, social determinants, China

Word counts: 266 for the abstract and 3,181 for the main body of text

For peer review only - http://bmjopen.bmj.com/site/about/guidelines.xhtml

For peer review only

Abstract:

Objective:

To investigate the association between maternal education and breastfeeding in the Chinese population, with the consideration of household income and health-seeking behaviors.

Design:

A nationally representative population-based cross-sectional study

Setting:

77 counties from 12 geographically distinct regions in China

Participants:

10,408 mothers, aged 15-53 years old (Mean: 29.15, SD: 5.11) were classified into primary school and below group (n=781), middle school group (n=3,842), high school/vocational school group (n=1,990), college and above group (n=3,795), according to their highest education.

Outcomes:

Five breastfeeding outcomes, including early initiation of breastfeeding, exclusive breastfeeding under 6 month, predominant breastfeeding under 6 months, current breastfeeding, and children ever breastfed, were calculated based on the standardized questionnaire from the WHO and Wellstart International's toolkit for monitoring and evaluating breastfeeding activities.

Results:

Maternal education is positively related to early initiation of breastfeeding and inversely related to exclusive breastfeeding in the adjusted model. After stratification by household income, the positive relationship between maternal education and early initiation of breastfeeding is only observed in the group with high household income. An inverse relationship between maternal education and exclusive breastfeeding exists in low and high household income groups, and the association is stronger in low household income group.

Conclusions:

For peer review only - http://bmjopen.bmj.com/site/about/guidelines.xhtml

Mothers with a higher education were more likely to early initiate breastfeeding while less likely to exclusively breastfeed their babies. Routine and successful nursing are crucial for the health of infants and largely influenced by social factors such as maternal education observed in the present study. Future public health interventions on breastfeeding promotion should take into consideration the issues related to the educational level of mothers.

Strengths and limitations of this study

The present study is a nationally representative population-based study with large sample size and is based on the first survey conducted in China to specifically explore the social determinants of breastfeeding.

This is the first study to investigate the relationship between maternal education and comprehensive breastfeeding patterns in a national scale in China.

We specified breastfeeding indicators and explored the associations separately and stratified by possible influential factors.

Maternal education in this study represents formal education gained through schooling and it may not reflect the actual health literacy of the mothers

Feeding practices were determined using the 'last-24-hour method' which tends to overestimate the prevalence of EBF as compared to the 'since birth method'

Funding

This study was supported by Bill & Melinda Gates Foundation and China Development Research Foundation.

For peer review only

INTRODUCTION

Extensive research has reported diverse and compelling advantages of breastfeeding for infants, mothers, families, and society.[1] Breastfeeding has become more common in the last few years because of numerous interventions conducted at multiple levels from the communities, hospitals, and the society in the whole, but the rates of breastfeeding still remain below optimal levels as recommended by the world health organization in many countries.[2 3] According to a recent, cross-sectional report of 55 counties from 30 provinces in China, the prevalence of ever breastfeeding was 79.6%, and only 20.8% of 14,539 children surveyed were exclusively breastfed at 6 months.[4]

WHO recognizes almost all mothers as biologically capable to breastfeed, except in a small number of health conditions, that use of breast milk substitutes is justifiable.[5] Breastfeeding practices, however, are not merely biological issues but are also related to health behavior and influenced by multifactorial determinants, including historical, socioeconomic, and cultural factors.[6] Women's breastfeeding practices are also affected by personal attributes such as age, weight, education, and confidence.[6] In China, breastfeeding practice face unique challenges, due to its diverse cultural values, political system, and historical perception of breastfeeding.[7 8]

Education status of the mother has been identified as an important determinant for child welfare, survival, and health.[9 10] Although maternal education and breastfeeding practices have been studied extensively, findings and conclusions are inconsistent.[11-14] Studies conducted in Nepal, United States, and Italy showed that higher maternal education was related to better breastfeeding practices.[11-13] Others studies found that the relationship between maternal education and breastfeeding practices differed among various social classes.[15]

Most of the studies conducted in China explored the effect of maternal education on breastfeeding together

For peer review only - http://bmjopen.bmj.com/site/about/guidelines.xhtml

with other socioeconomic factors. The results across studies were different and sometimes reached the opposite conclusion.[16] Using the data from 12 regions in China, the aim of this study is to investigate the association between maternal education and breastfeeding, taking into account household income and health-seeking behaviors. The findings of this study contribute to the growing body of evidence on social determinants of breastfeeding and have the potential to provide evidence for related interventions and policies.

METHODS

Study design and participants

This population-based study was conducted in 12 geographically defined regions in China. Multi-stage sampling technique was adopted for selection participants. In the first stage, population proportionate sampling (PPS) method was applied, in consideration of the population structure and the status of maternal and child health and of socio-economic development. In the end, 4 megacities (Beijing in North China, Nanning of Guangxi Province in South Central China, Hefei of Anhui province and Nanjing of Jiangsu province in East China), 4 medium-sized cities (Longyan of Fujian province in East China, Liaoyuan of Jilin province in Northeast China, Honghe of Yunnan province and Luzhou of Sichuan province in Southwest China), 2 countryside areas (Zhengding County of Hebei province in North China and Jia County of Henan province in Central China), and 2 underdevelopped rural areas (Tailai county of Heilongjiang province in Northeast China and Ledu county of Qinghai province in Northwest China) were selected. In the second stage, one district or county was chosen in the 12 regions by simple random sampling method. In the third stage, simple sampling method was implemented to select 4-8 communities or villages from the chosen district or county in the 12 regions. A survey team, 5 full-time staff with medical qualifications and field experiences, was established in each of the 12 regions. Mothers whose children were under 12 months were eligible for this study. To encourage involvement, incentives, such as shopping cards and simple household commodities, were prepared for each participant. By the end, 10,408 mothers from 77 counties were recruited for the present

For peer review only - http://bmjopen.bmj.com/site/about/guidelines.xhtml

study. After registration and obtaining informed consent, trained health staff with smartphone-based questionnaire collected participants' information. The questionnaire asked for mothers' socio-demographic information, socio-economic information, breastfeeding behavior, and breastfeeding related environment. Over 90% of participants completed the entire questionnaire. The study has been approved by the Institutional Review Boards at Peking University Health Science Center and the China National Center for Disease Control.

Variable definitions

Maternal education is the main exposure in the study. In the baseline survey, maternal education was classified into eight categories, including no formal school, not graduated from primary school, primary school, middle school, high/vocational school, associate/junior college, university, and postgraduate and above. In the present study, the first three categories were combined as primary school and below and the last three categories were combined as college and above in order to balance the population in each group.

Four breastfeeding outcome variables including early initiation of breastfeeding (EIB), exclusive breastfeeding under 6 months (EBF), predominant breastfeeding under 6 months (PBF), and children ever breastfed (Ever BF), were analyzed. EIB and ever BF were defined according to World Health Organization indicators.[17] The final calculation of EIB prevalence and ever BF prevalence was conducted among mothers with children from 0-12 months of age. EIB prevalence was defined as proportion of mothers with children born in the last 12 months who were put to the breast within an hour after birth. Ever BF prevalence was defined as proportion of children born in the last 12 months who were ever breastfed. EBF and PBF were defined and evaluated according to Wellstart International's toolkit for monitoring and evaluating breastfeeding activities,[18] using a 24-hour recall methodology. Mothers were asked to recall the food they fed their children in the last 24 hours, and the final calculation of EBF prevalence and PBF prevalence was conducted among mothers with children from 0-6 months. EBF prevalence was defined as proportion of

For peer review only - http://bmjopen.bmj.com/site/about/guidelines.xhtml

infants 0–6 months of age who were fed exclusively breast milk. PBF prevalence was defined as proportion of infants 0–6 months of age who were predominantly breastfed, which mainly comprised of children who were fed by breast milk and water.[18] Furthermore, to explore mothers' current breastfeeding situation, the present study also implemented the indicator of current breastfeeding (CBF) prevalence, defined as any breastfeeding in the last 24 hours of children less than 12 months of age.[18]

Mother-infant indicators and family socio-economic status were the two categories of covariates incorporated in the present study. Mother-infant indicators included maternal age, pre-pregnancy BMI, gestational age, infant birth weight, infant sex, parity, and delivery method. The information of maternal age, gestational age, infant birth weight, infant sex, parity and delivery methods were collected based on mothers' self-reported answers. Pre-pregnancy BMI was calculated based on mothers' self-reported weight and height before pregnancy. Apart from maternal education, family socio-economic status includes: household income, residency status, and maternal occupation. These covariates were also collected based on participants' self-reported answers. In the baseline survey, household income was a continuous variable. In the present study, household income was classified into ≤40,000 Yuan (1 USD≈6.33 Chinese Yuan); 40,001-80,000 Yuan; and ＞80,000 Yuan. Occupation was categorized as agriculture and related workers, factory workers, white-collar, and others. Residency status was classified as local or migrant, according to whether the mother lives at the place of domicile. Health-seeking behaviors, including considering breastfeeding before pregnancy, attending postpartum mother support group, and receiving breastfeeding education during the perinatal period, were analyzed in the present study.

Data analysis

Descriptive analyses were used to illustrate the basic demographic, socioeconomic and lifestyle characteristics in different maternal education groups. The data of mothers who did not complete the questionnaires as

For peer review only - http://bmjopen.bmj.com/site/about/guidelines.xhtml

required were excluded from multivariable analyses. The association between maternal education and breastfeeding outcomes was analyzed using logistic regression model. All the odds ratios were presented with 95% confidence interval. Primary school and uneducated group was chosen to be reference groups in all models. Two logistic models were fitted: 1) unadjusted; 2) adjusted for maternal age, infant birth weight, infant sex, parity, delivery method, household income, region (super city/urban/rural/poor rural), residency status (local/migrant), and maternal occupation.

To further understand the relationship between maternal education and early initiation of breastfeeding and exclusive breastfeeding, the adjusted prevalence of early initiation of breastfeeding and exclusive breastfeeding in different maternal education groups was calculated after stratification by household income. The adjusted prevalence of health-seeking behaviors, including considering breastfeeding before pregnancy, attending postpartum mother support group, and receiving breastfeeding education were also calculated in different education groups. The adjusted prevalence was based on logistic regression and the detailed method was described elsewhere.[19] All the analyses were conducted using SAS version 9.4 (SAS Institute, Cary, North Carolina, USA).

Patient involvement

Patients were not involved in setting the research questions or planning the study. Investigators do not know the identity of study participants.

RESULTS

Table 1 shows the baseline characteristics of the study population by maternal education. Of all 10,408 participants included, 7.50% had the highest education at primary school or below, 36.91% had middle school education, 19.12% had high/vocational school education, and 36.46% had college education and above. The

For peer review only - http://bmjopen.bmj.com/site/about/guidelines.xhtml

mean ages for those whose highest education was primary school and below, middle school, high/vocational school, and college and above were 28.76 (SD=6.68), 28.41 (SD=5.21), 28.26 (SD=4.78), and 30.36 (SD=4.51), respectively. The distributions of pre-pregnancy BMI, gestational age, infant birth weight, and infant sex were similar across all maternal education groups. Women with a primary school and below education tended to be multiparous, had vaginal delivery method, lived in urban and poor rural areas, and had lower household income. Women who had primary school education and/or below were more likely to hold agricultural jobs (57.89%) and were less likely to be white-collar (11.77%). Women whose educational level were college and above were more likely to be migrant residents (46.35%) compared to women of other educational levels.

Table 2 presents the relationship between breastfeeding practices and maternal education. No significant association was found between CBF and maternal education. Compared to the primary school and below group, other groups showed a greater odds ratio of early initiation and ever breastfeeding. Compared to the primary school and below group, other groups had a smaller odds ratio of exclusive and predominant breastfeeding. After adjusting for possible confounders, the significant association between maternal education and breastfeeding outcomes was only found in early initiation of breastfeeding and exclusive breastfeeding. The detailed ORs and 95% CIs could be referred in Table 2.

Figure 1 presents the adjusted prevalence of early initiation of breastfeeding in different maternal education groups, stratified by household income. The association between maternal education and early initiation of breastfeeding was only significant in those with household income >80,000 Yuan. In this group, there was an increase of the prevalence of early initiation of breastfeeding from 44.95% (95% CI: 40.65%-49.25%) in those with primary school education and below to 78.39% (95% CI: 76.25%-80.52%) in those with college education and above.

For peer review only - http://bmjopen.bmj.com/site/about/guidelines.xhtml

Figure 2 presents the adjusted prevalence of exclusive breastfeeding in different maternal education groups, stratified by household income. There was no significant association between maternal education and exclusive breastfeeding prevalence in those with household income of 40,001-,80,000 Yuan. For those with household income of ≤40,000 Yuan and ＞80,000 Yuan, there was a general negative association between maternal education and incidence of exclusive breastfeeding. For those with household income of ≤40,000 Yuan, the prevalence of exclusive breastfeeding decreased from 36.54% (95% CI: 29.93%-43.15%) in those with primary school education and below to 11.61% (95% CI: 10.58%-12.65%) in those with college education and above. For those with a household income of >80,000 Yuan, the prevalence of exclusive breastfeeding decreased from 26.83% (95% CI: 26.63%-27.03%) in those with a primary and below education to 9.69% (95% CI: 8.14%-11.23%) in those with a college education.

Figure 3 presents the adjusted prevalence of health-seeking behaviors, including considering breastfeeding before pregnancy, attending mother support group postpartum, and receiving breastfeeding education. There was a general positive association between health-seeking behaviors and maternal education across these three factors. The prevalence of considering breastfeeding before pregnancy increased from 28.35% (95% CI: 25.98%-30.73%) in those with primary school education and below to 53.92% (95% CI: 52.09%-55.75%) in those with college education and above. The prevalence of attending mother support group postpartum increased from 10.09% (95% CI: 9.20%-10.98%) in those with primary school education and below to 56.03% (95% CI: 54.55%-57.51%) in those with college education and above. The prevalence of receiving breastfeeding education increased from 32.65% (95% CI: 30.71%-34.59%) in those with primary school education and below to 66.47% (95% CI: 65.16%-67.78%) in those with college education and above.

DISCUSSION

For peer review only - http://bmjopen.bmj.com/site/about/guidelines.xhtml

Several important findings are illustrated in the present study. First, maternal education is inversely related to exclusive breastfeeding. Second, maternal education is positively related to early initiation of breastfeeding in the high household income group. Third, after stratification by income, the patterns of association between maternal education and breastfeeding indicators differ in different income groups. The positive relationship between maternal education and early initiation of breastfeeding is observed in the group with high household income. The inverse relationship between maternal education and exclusive breastfeeding exists in low and high household income groups, and the association is stronger in low household income group.

This study is based on a large, national representative, population-based survey. Large sample size ensures high precision of the findings. This is one of the few studies focused mainly on the association between maternal education and breastfeeding practices in China. We specified breastfeeding indicators and explored the associations separately and stratified by possible influential factors. Quantitative data were used to analyze health-seeking behavior. Admittedly, there are some potential limitations of the present study. First, causal inference between maternal education and breastfeeding practices is limited due to the study being cross-sectional. Further perspective study is needed in order to establish a causal relationship. Second, maternal education in this study represents formal education gained through schooling and it may not reflect the actual health literacy of the mothers. Lastly, feeding practices were determined using the 'last-24-hour method' which tends to overestimate the prevalence of EBF as compared to the 'since birth method' .[20]

In contrast to early initiation of breastfeeding, maternal education is inversely related to exclusive breastfeeding. This is consistent with previous studies conducted in China. A systematic review conducted in China exploring the relationship between maternal education and breastfeeding found a negative association between breastfeeding indicators and maternal education. The review speculated that maternal leave, occupation, and incorrect traditional perceptions contributed to the negative association.[16] One of the

For peer review only - http://bmjopen.bmj.com/site/about/guidelines.xhtml

traditional perceptions was that breastfeeding would change mothers' body shape and have negative impact on mothers' health.[21] Nevertheless, our finding is opposite to those in the developed countries.[12 13] In China, highly educated mothers are usually working full time in responsible and formal positions. The maternity leave regulated by legislation is 98 days in China,[22] which is not enough to guarantee the six-month exclusive breastfeeding time as recommended by WHO. It is also equally difficult for mothers who have already returned to work to continue breastfeeding due to lack of related supporting facilities and working pressure.[23] Another possible explanation is that highly educated mothers tend to have higher household income, and thus are able to afford infant formula, which is especially true in China.[24] Due to infant formula advertisement and lack of proper breastfeeding education, use of infant formula is sometimes regarded as healthier and also a symbol of wealth in China.[24 25] Besides, incorrect perceptions in some areas maintain that breastfeeding changes mothers' body shape.[21] Women with higher education generally in live in urban areas and in a relatively higher social class. Women in urban areas and at higher social classes may face a higher social pressure. Thus, they may pay more attention to their own body shape and are more willing to choose infant formula.[26]

In the present study, positive association between early initiation of breastfeeding and maternal education is observed in those with household income >80,000 Yuan. This result is consistent with studies done in the developed countries,[12 13 27] yet opposite to those completed in the developing countries. A study conducted in Ethiopia found that higher maternal education level was associated with lower rate of EBF.[28] A nation- wide study in Bangladesh found that higher education was a risk factor for an infant not being exclusively breastfed.[29] The positive association in this study needs to be explored. In the present study, mothers with have higher educational attainment tend to exhibit proactive health behaviors, early consideration of feeding methods and more likely to receive breastfeeding education, all of which may foster early initiation of breastfeeding. It has been suggested that women with higher education tend to have stronger social support,[30]

For peer review only - http://bmjopen.bmj.com/site/about/guidelines.xhtml

and more likely to attend mother support group as observed in this study. Previous study found that social support is an important contributor to early initiation of breastfeeding.[31 32] Furthermore, women with higher education are more likely to live in super cities and have higher household income. There exists a huge disparity of health services between rural and urban areas in China.[33] Thus, the women who live in super cities are more likely to have better access to quality health services. Previous studies found that professional knowledge of nurses and doctors may foster early initiation of breastfeeding.[34 35] A review conducted in India suggested that interventions designed to increase the knowledge and skills of healthcare workers were important to promote early initiation of breastfeeding.[36]

After stratification by income, the patterns of association between maternal education and breastfeeding indicators differ in different income groups. The positive relationship between maternal education and early initiation of breastfeeding is only significant in the high household income group. Higher education may promote better breastfeeding knowledge and health behavior.[37] Nevertheless, apart from knowledge, early initiation of breastfeeding require social support and access to healthcare services.[3] Women with lower household income may not have sufficient financial abilities to attain healthcare services. These women are more likely to give birth at home, where initiation of breastfeeding within 1 hour of birth may not be promoted. Mothers may recognize the benefits of delivery at healthcare facilities, yet they may not be able to afford the expensive hospitals stays and professional services. The inverse relationship between maternal education and exclusive breastfeeding was the strongest in low household income group. A possible explanation is that women with lower income and higher education tend to have heavier workload in order to meet the demands of the family, consequently shortened exclusive breastfeeding duration. People with lower income tend to live in rural areas, where health infrastructure and expertise of health workers are less advanced than that in urban areas. Traditional values may also be more prevalent than in the cities, such as routine supplementary feeding with water,[38] giving rise to the trend found in this study.

For peer review only - http://bmjopen.bmj.com/site/about/guidelines.xhtml

Conclusions

A positive association between maternal education and early initiation of breastfeeding and a negative association between maternal education and exclusive breastfeeding were observed in this study, with the consideration of health seeking behavior and household income. Routine and successful breastfeeding are crucial for the health of the infant and is largely influenced by social factors. This study underscores the influence of maternal education on breastfeeding practices. Findings from this study contribute to the growing body of research on social determinants of breastfeeding. This study suggests that future public health interventions on breastfeeding promotion in China should take into consideration of the effect of maternal education and deliver specific education and intervention according to mother's educational status.

Contributors

KT and HW contributed to the study concept and design, statistical analysis, results interpretation, and drafting and revision of the manuscript. ST contributed to the study concept and design, drafting and revision of the manuscript. TX contributed to the study concept and design, revision of the manuscript. YW contributed to revision of the manuscript. YL and XQ contributed to the study concept and design and results interpretation. JG contributed to the study concept and design. All authors read and approved the final version of the manuscript.

Funding

This study was supported by Bill & Melinda Gates Foundation and China Development Research Foundation.

Competing interests

None declared.

Patient consent

Not required.

For peer review only - http://bmjopen.bmj.com/site/about/guidelines.xhtml

Ethics approval

This study was conducted according to the guidelines laid down in the Declaration of Helsinki and all procedures involving human subjects/patients has been approved by the Institutional Review Boards at Peking University Health Science Center and the China National Center for Disease Control. The participants included in this study provided written informed consent.

Data sharing statement

Data are available upon reasonable request

Acknowledgement

We are grateful for Chinese Center For Disease Control And Prevention for providing the data.

References

1. Eidelman AI, Schanler RJ, Johnston M, et al. Breastfeeding and the use of human milk. *Pediatrics* 2012;129(3):e827-e41.

2. Cai X, Wardlaw T, Brown DW. Global trends in exclusive breastfeeding. *International breastfeeding journal* 2012;7(1):12.

3. Victora CG, Bahl R, Barros AJ, et al. Breastfeeding in the 21st century: epidemiology, mechanisms, and lifelong effect. *The Lancet* 2016;387(10017):475-90.

4. Yang Z, Lai J, Yu D, et al. Breastfeeding rates in China: a cross-sectional survey and estimate of benefits of improvement. *The Lancet* 2016;388:S47.

5. World Health Organization. Acceptable medical reasons for use of breast-milk substitutes. 2009

6. Rollins NC, Bhandari N, Hajeebhoy N, et al. Why invest, and what it will take to improve breastfeeding practices? *The Lancet* 2016;387(10017):491-504.

7. Xu F, Qiu L, Binns CW, et al. Breastfeeding in China: a review. *International Breastfeeding Journal* 2009;4(1):6.

For peer review only - http://bmjopen.bmj.com/site/about/guidelines.xhtml

8. Jiang H, Li M, Yang D, et al. Awareness, intention, and needs regarding breastfeeding: findings from first-time mothers in Shanghai, China. *Breastfeeding Medicine* 2012;7(6):526-34.

9. Meyrose A-K, Klasen F, Otto C, et al. Benefits of maternal education for mental health trajectories across childhood and adolescence. *Social Science & Medicine* 2018;202:170-78.

10. Güneş PM. The role of maternal education in child health: Evidence from a compulsory schooling law. *Economics of Education Review* 2015;47:1-16.

11. Acharya P, Khanal V. The effect of mother's educational status on early initiation of breastfeeding: further analysis of three consecutive Nepal Demographic and Health Surveys. *BMC public health* 2015;15(1):1069.

12. Heck KE, Braveman P, Cubbin C, et al. Socioeconomic status and breastfeeding initiation among California mothers. *Public health reports* 2006;121(1):51-59.

13. Bertini G, Perugi S, Dani C, et al. Maternal education and the incidence and duration of breast feeding: a prospective study. *Journal of pediatric gastroenterology and nutrition* 2003;37(4):447-52.

14. Desai S, Alva S. Maternal education and child health: Is there a strong causal relationship? *Demography* 1998;35(1):71-81.

15. Skafida V. The relative importance of social class and maternal education for breast-feeding initiation. *Public health nutrition* 2009;12(12):2285-92.

16. Zhao J, Zhao Y, Du M, et al. Maternal education and breastfeeding practices in China: A systematic review and meta-analysis. *Midwifery* 2017;50:62-71.

17. World Health Organization. Indicators for assessing infant and young child feeding practices: conclusions of a consensus meeting held 6-8 November 2007 in Washington DC, USA: World Health Organization (WHO) 2008.

18. Lung'aho M, Huffman SL, Labbok MH, et al. Tool kit for monitoring and evaluating breastfeeding practices and programs. *Washington, DC: Wellstart International* 1996

For peer review only - http://bmjopen.bmj.com/site/about/guidelines.xhtml

19. Santos CAS, Fiaccone RL, Oliveira NF, et al. Estimating adjusted prevalence ratio in clustered cross-sectional epidemiological data. *BMC Medical Research Methodology* 2008;8(1):80.

20. Khanal V, Lee AH, Scott JA, et al. Implications of methodological differences in measuring the rates of exclusive breastfeeding in Nepal: findings from literature review and cohort study. *BMC pregnancy and childbirth* 2016;16(1):389.

21. Gao Ll, Chan SWc, You L, et al. Experiences of postpartum depression among first-time mothers in mainland China. *Journal of Advanced Nursing* 2010;66(2):303-12.

22. Jia N, Dong X-y, Song Y-p. Paid Maternity Leave and Breastfeeding in Urban China. *Feminist Economics* 2017:1-23.

23. Bai DL, Fong DYT, Tarrant M. Factors associated with breastfeeding duration and exclusivity in mothers returning to paid employment postpartum. *Maternal and child health journal* 2015;19(5):990-99.

24. Kent G. Global infant formula: monitoring and regulating the impacts to protect human health. *International breastfeeding journal* 2015;10(1):6.

25. Tang L, Lee AH, Binns CW, et al. Widespread usage of infant formula in China: a major public health problem. *Birth* 2014;41(4):339-43.

26. Gottschang SZ. Infant Feeding and the Feminine Body in Urban China. *China urban: Ethnographies of contemporary culture* 2001

27. Meedya S, Fahy K, Kable A. Factors that positively influence breastfeeding duration to 6 months: a literature review. *Women and Birth* 2010;23(4):135-45.

28. Alemayehu T, Haidar J, Habte D. Determinants of exclusive breastfeeding practices in Ethiopia. *Ethiopian Journal of Health Development* 2009;23(1)

29. Mihrshahi S, Kabir I, Roy SK, et al. Determinants of infant and young child feeding practices in Bangladesh: secondary data analysis of Demographic and Health Survey 2004. *Food and nutrition bulletin* 2010;31(2):295-313.

For peer review only - http://bmjopen.bmj.com/site/about/guidelines.xhtml

30. Heaney CA, Israel BA. Social networks and social support. *Health behavior and health education: Theory, research, and practice* 2008;4:189-210.

31. Patel A, Bucher S, Pusdekar Y, et al. Rates and determinants of early initiation of breastfeeding and exclusive breast feeding at 42 days postnatal in six low and middle-income countries: A prospective cohort study. *Reproductive health* 2015;12(2):S10.

32. Bai DL, Fong DYT, Lok KYW, et al. Relationship between the infant feeding preferences of Chinese mothers' immediate social network and early breastfeeding cessation. *Journal of Human Lactation* 2016;32(2):301-08.

33. Liu M, Zhang Q, Lu M, et al. Rural and urban disparity in health services utilization in China. *Medical care* 2007;45(8):767-74.

34. Balogun OO, Dagvadorj A, Anigo KM, et al. Factors influencing breastfeeding exclusivity during the first 6 months of life in developing countries: a quantitative and qualitative systematic review. *Maternal & child nutrition* 2015;11(4):433-51.

35. Sharma IK, Byrne A. Early initiation of breastfeeding: a systematic literature review of factors and barriers in South Asia. *International breastfeeding journal* 2016;11(1):17.

36. Aguayo VM, Gupta G, Singh G, et al. Early initiation of breastfeeding on the rise in India. *BMJ global health* 2016;1(2):e000043.

37. Adler NE, Boyce T, Chesney MA, et al. Socioeconomic status and health: the challenge of the gradient. *American psychologist* 1994;49(1):15.

38. Raven JH, Chen Q, Tolhurst RJ, et al. Traditional beliefs and practices in the postpartum period in Fujian Province, China: a qualitative study. *BMC Pregnancy and Childbirth* 2007;7(1):8.

For peer review only - http://bmjopen.bmj.com/site/about/guidelines.xhtml

Table 1 Demographic and social-economic characteristics of the participants

	Maternal Education			
	Primary school and below (N=781)	Middle School (N=3842)	High School/Vocational School (N=1990)	College and above (N=3795)
Mean maternal age, year (SD)	28.76(6.68)	28.41(5.21)	28.26(4.78)	30.36(4.51)
Mean pre-pregnancy BMI, Kg/m2 (SD)	22.67(4.66)	22.44(4.16)	21.74(4.04)	21.75(4.12)
Mean gestational age, week (SD)	39.02(1.38)	39.03(1.40)	38.94(1.51)	38.98(1.38)
Mean infant birth weight, kg (SD)	3.23(0.47)	3.33(0.59)	3.34(0.67)	3.43(0.75)
Infant sex, %				
Male	50.67	50.64	52.17	50.08
Female	49.33	49.36	47.83	49.92
Parity, %				
Primiparous	29.70	33.87	43.49	62.06
Multiparous	70.30	66.13	56.51	37.94
Delivery method, %				
Vaginal	65.77	56.72	54.59	57.01
Cesarean section	34.23	43.28	45.41	42.99
Region, %				
Super city	9.70	14.16	30.82	61.55
Urban	43.15	32.08	39.09	27.41
Rural	7.15	25.39	21.09	7.29
Poor rural	40.00	28.37	9.00	3.75
Household income, %				
≤40,000 yuan	61.74	37.44	20.06	11.81
40,000-79,999 yuan	22.39	30.01	26.21	13.82
≥80,000 yuan	3.48	7.84	16.40	38.89
Maternal Occupation, %				
Agriculture related	57.89	33.61	9.37	2.27
Factory workers	11.53	19.95	20.31	8.66
White-collar	11.77	26.74	47.23	73.03
Others[1]	18.81	19.71	23.09	16.04
Resident status, %				
Local	69.70	69.15	59.50	53.65
Migrant	30.30	30.85	40.50	46.35
Breastfeeding practice, %				
Early initiation	64.85	68.24	71.11	77.21
Current BF	87.88	88.53	85.09	86.21
Ever BF	95.52	97.31	97.65	97.93
Exclusive BF (0-6 months)	26.53	15.36	13.64	14.06
Predominant BF (0-6 months)	50.00	39.99	18.44	39.29
Health Seeking Behaviors				
Considered feeding method	28.31	45.35	50.24	55.29

[1] Others refer to unidentified occupation, unemployed, and housewives

For peer review only

before pregnancy				
Attended mother support group	8.24	24.40	38.20	49.36
Received breastfeeding education	38.59	47.23	59.13	64.23

Table 2. Relations between breastfeeding practices and maternal education*

	Unadjusted OR (95% CI)			
	Primary school and below	Middle School	High School/Vocational School	College and above
Early initiation	1	1.17(0.99,1.37)	1.34(1.12,1.59)	1.84(1.56,2.16)
Current BF	1	1.07(0.84,1.34)	0.79(0.62,1.00)	0.86(0.69,1.08)
Ever BF	1	1.70(1.16,2.49)	1.95(1.25,3.03)	2.23(1.50,3.31)
Exclusive BF (0-6 months)	1	0.50(0.39,0.65)	0.44(0.33,0.58)	0.45(0.35,0.59)
Predominant BF (0-6 months)	1	0.67(0.54,0.83)	0.40(0.32,0.51)	0.28(0.22,0.35)
	Adjusted OR (95% CI)			
	Primary school and below	Middle School	High School/Vocational School	College and above
Early initiation	1	1.55(1.18,2.05)	1.64(1.19,2.28)	2.06(1.44,2.95)
Current BF	1	0.85(0.55,1.32)	0.67(0.41,1.09)	0.96(0.57,1.62)
Ever BF	1	1.42(0.69,2.93)	1.43(0.59,3.45)	1.80(0.70,4.62)
Exclusive BF (0-6 months)	1	0.45(0.31,0.66)	0.59(0.37,0.95)	0.51(0.31,0.84)
Predominant BF (0-6 months)	1	0.75(0.53,1.10)	0.81(0.54,1.23)	0.64(0.62,0.97)

**Adjusted for maternal age, infant birth weight, infant sex, parity, delivery method, household income, region (super city/urban/rural/poor rural), residency (local/migrant), occupation*

For peer review only

For peer review only - http://bmjopen.bmj.com/site/about/guidelines.xhtml

Figure 1 Adjusted Prevalence* of early initiation in different maternal education groups, stratified by household Income

**After adjusting for maternal age, infant sex, parity, delivery method, paternal education, region (super city/urban/rural/poor rural), residency (local/migrant), occupation*

Figure 2 Adjusted Prevalence* of exclusive breastfeeding in different maternal education groups, stratified by household Income

*After adjusting for maternal age, infant sex, parity, delivery method, paternal education, region (super city/urban/rural/poor rural), residency (local/migrant), occupation

Figure 3 Adjusted Prevalence* of considering feeding method before pregnancy, attending mother support group, receiving breastfeeding education in different maternal education groups*

**After adjusting for maternal age, infant sex, parity, delivery method, paternal education, household income, region (super city/urban/rural/poor rural), residency (local/migrant), occupation.*

For peer review only - http://bmjopen.bmj.com/site/about/guidelines.xhtml

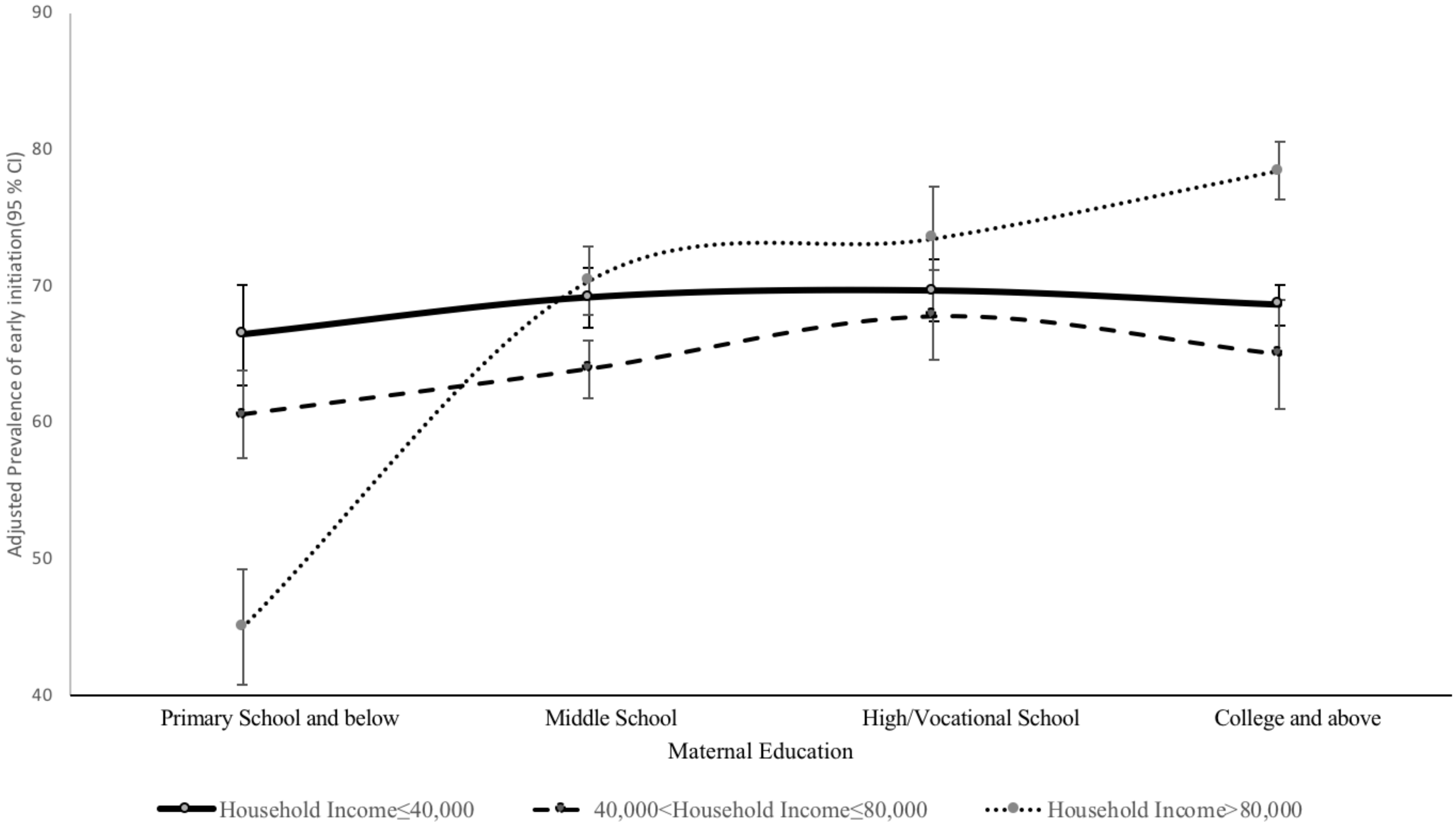
90
80
70
60
50
40
Adjusted Prevalence of early initiation(95 % CI)
Primary School and below
Middle School
High/Vocational School
College and above
Maternal Education
Household Income≤40,000
40,000<Household Income≤80,000
Household Income>80,000

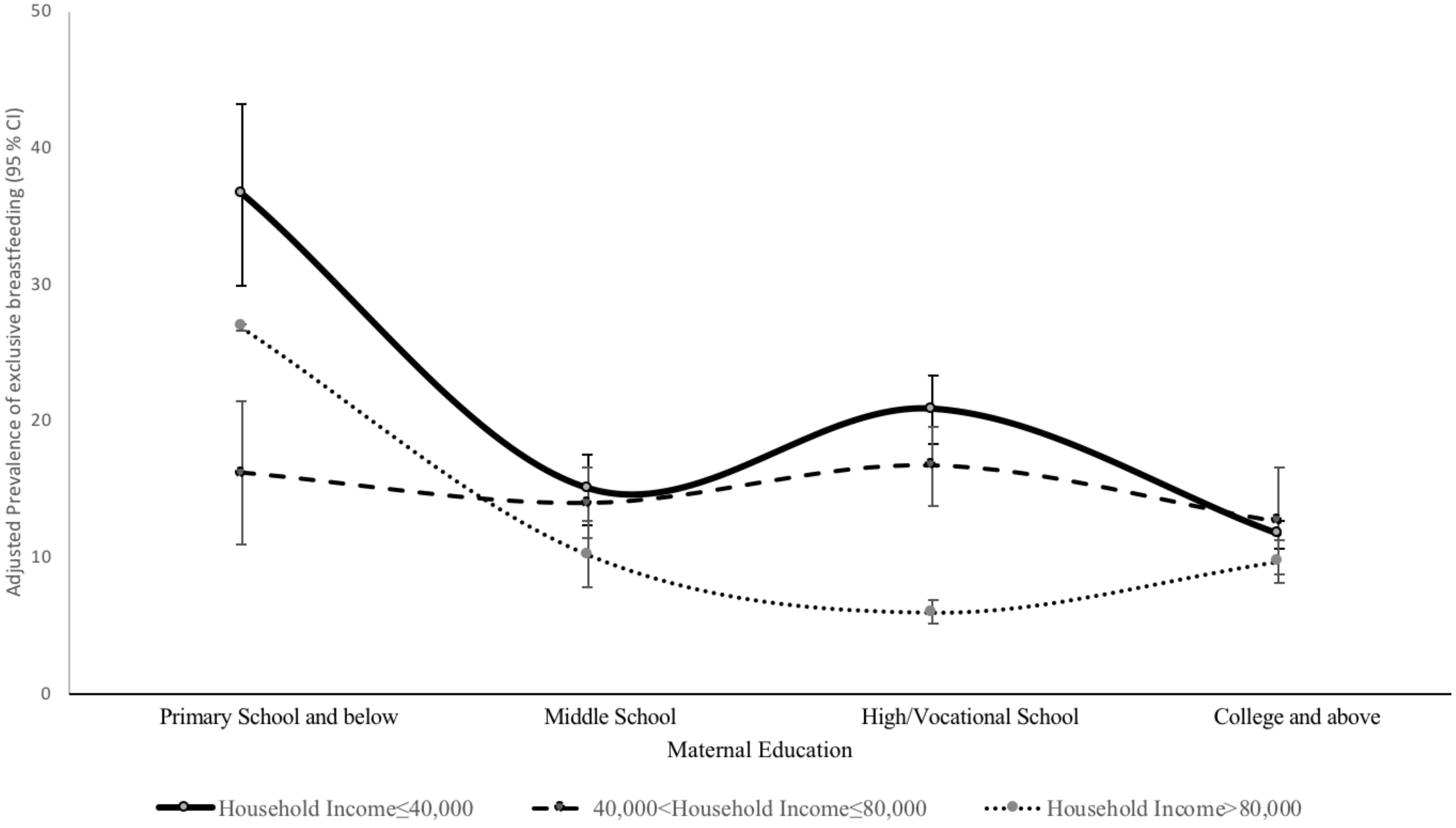

50
40
30
20
10
0
Adjusted Prevalence of exclusive breastfeeding (95 % CI)
Primary School and below
Middle School
High/Vocational School
College and above
Maternal Education
Household Income≤40,000
40,000<Household Income≤80,000
Household Income>80,000

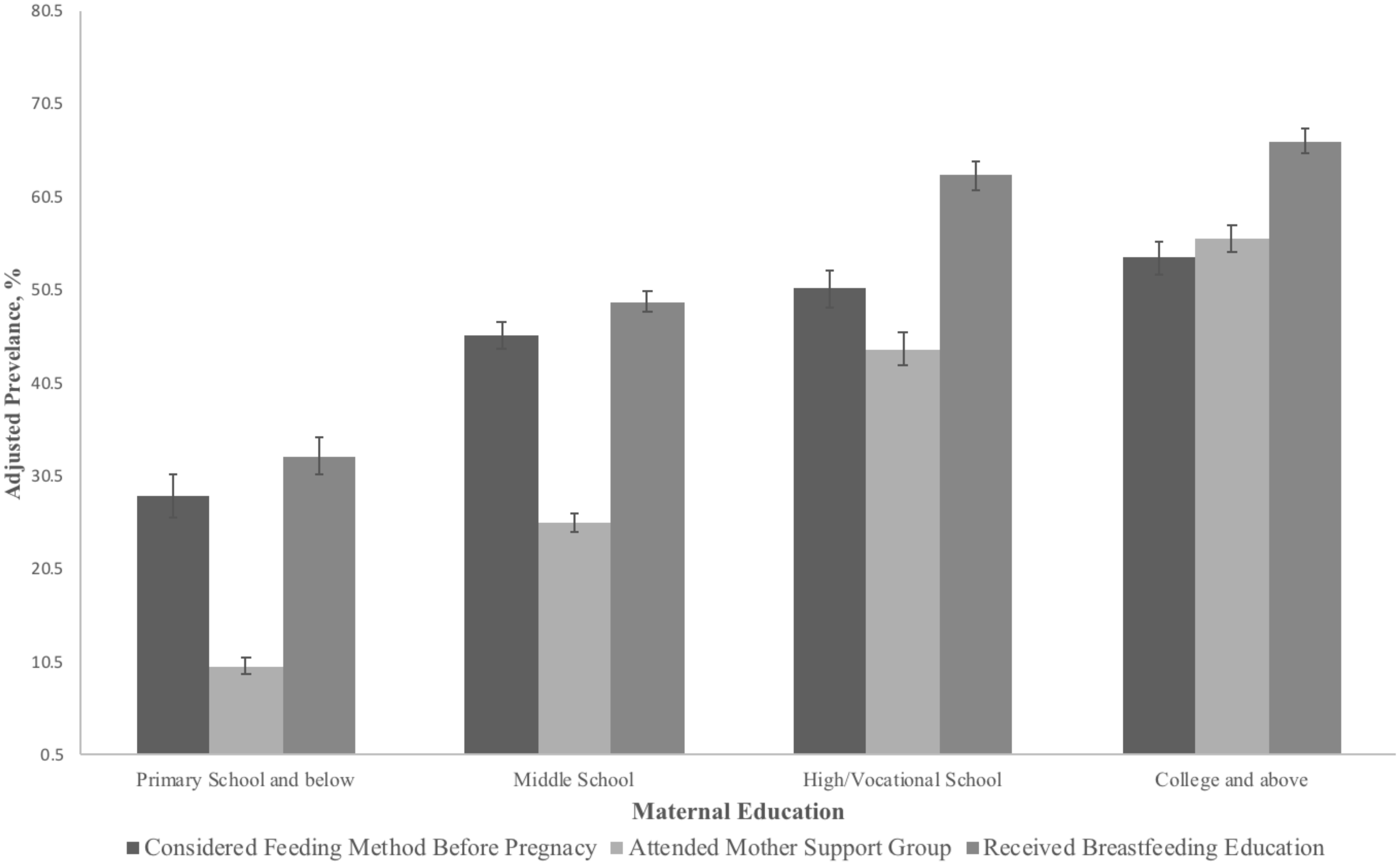
80.5
70.5
60.5
50.5
40.5
30.5
20.5
10.5
0.5
Adjusted Prevelance, %
Primary School and below
Middle School
High/Vocational School
College and above
Maternal Education
Considered Feeding Method Before Pregnacy
Attended Mother Support Group
Received Breastfeeding Education

Reporting checklist for cross sectional study.

Based on the STROBE cross sectional guidelines.

Instructions to authors

Complete this checklist by entering the page numbers from your manuscript where readers will find each of the items listed below.

Your article may not currently address all the items on the checklist. Please modify your text to include the missing information. If you are certain that an item does not apply, please write "n/a" and provide a short explanation.

Upload your completed checklist as an extra file when you submit to a journal.

In your methods section, say that you used the STROBE cross sectional reporting guidelines, and cite them as:

von Elm E, Altman DG, Egger M, Pocock SJ, Gotzsche PC, Vandenbroucke JP. The Strengthening the Reporting of Observational Studies in Epidemiology (STROBE) Statement: guidelines for reporting observational studies.

For peer review only

		Reporting Item	Page Number
Title	#1a	Indicate the study’s design with a commonly used term in the title or the abstract	1
Abstract	#1b	Provide in the abstract an informative and balanced summary of what was done and what was found	2-3
Background / rationale	#2	Explain the scientific background and rationale for the investigation being reported	4
Objectives	#3	State specific objectives, including any prespecified hypotheses	5
Study design	#4	Present key elements of study design early in the paper	5
Setting	#5	Describe the setting, locations, and relevant dates, including periods of recruitment, exposure, follow-up, and data collection	5-6
Eligibility criteria	#6a	Give the eligibility criteria, and the sources and methods of selection of participants.	5-6
	#7	Clearly define all outcomes, exposures, predictors, potential	6-7

		confounders, and effect modifiers. Give diagnostic criteria, if applicable	
Data sources / measurement	#8	For each variable of interest give sources of data and details of methods of assessment (measurement). Describe comparability of assessment methods if there is more than one group. Give information separately for for exposed and unexposed groups if applicable.	7-8
Bias	#9	Describe any efforts to address potential sources of bias	6-8
Study size	#10	Explain how the study size was arrived at	5-6
Quantitative variables	#11	Explain how quantitative variables were handled in the analyses. If applicable, describe which groupings were chosen, and why	6-7
Statistical methods	#12a	Describe all statistical methods, including those used to control for confounding	7-8
	#12b	Describe any methods used to examine subgroups and interactions	7-8
	#12c	Explain how missing data were addressed	7-8
	#12d	If applicable, describe analytical methods taking account of sampling strategy	7-8
	#12e	Describe any sensitivity analyses	7-8
Participants	#13a	Report numbers of individuals at each stage of study—eg numbers potentially eligible, examined for eligibility, confirmed eligible, included in the study, completing follow-up, and analysed. Give information separately for for exposed and unexposed groups if applicable.	8-9
	#13b	Give reasons for non-participation at each stage	8-9
	#13c	Consider use of a flow diagram	8-9
Descriptive data	#14a	Give characteristics of study participants (eg demographic, clinical, social) and information on exposures and potential confounders. Give information separately for exposed and unexposed groups if applicable.	8-9
	#14b	Indicate number of participants with missing data for each variable of interest	8-9
Outcome data	#15	Report numbers of outcome events or summary measures. Give information separately for exposed and unexposed groups if applicable.	9
Main results	#16a	Give unadjusted estimates and, if applicable, confounder-adjusted	9-10

For peer review only

		estimates and their precision (eg, 95% confidence interval). Make clear which confounders were adjusted for and why they were included	
	#16b	Report category boundaries when continuous variables were categorized	9-10
	#16c	If relevant, consider translating estimates of relative risk into absolute risk for a meaningful time period	9-10
Other analyses	#17	Report other analyses done—e.g., analyses of subgroups and interactions, and sensitivity analyses	9-10
Key results	#18	Summarise key results with reference to study objectives	10-11
Limitations	#19	Discuss limitations of the study, taking into account sources of potential bias or imprecision. Discuss both direction and magnitude of any potential bias.	11
Interpretation	#20	Give a cautious overall interpretation considering objectives, limitations, multiplicity of analyses, results from similar studies, and other relevant evidence.	11-13
Generalisability	#21	Discuss the generalisability (external validity) of the study results	11-13
Funding	#22	Give the source of funding and the role of the funders for the present study and, if applicable, for the original study on which the present article is based	14

The STROBE checklist is distributed under the terms of the Creative Commons Attribution License CC-BY. This checklist was completed on 10. December 2018 using http://www.goodreports.org/, a tool made by the EQUATOR Network in collaboration with Penelope.ai

For peer review only

The association between maternal education and breastfeeding practices in China: a population-based cross-sectional study

Journal:	*BMJ Open*
Manuscript ID	bmjopen-2018-028485.R1
Article Type:	Research
Date Submitted by the Author:	17-Mar-2019

Complete List of Authors:	Tang, Kun; Tsinghua University, Research Center for Public Health<br>Wang, Hanyu; Peking University Health Science Centre, Institute for Medical Humanities<br>Tan, Shi Hui; Peking University Health Science Centre, School of Basic Medical Sciences<br>Xin, Tong; Peking University Health Science Centre, Department of Global Health<br>Wang, Yuqi; Peking University Health Science Centre, Institute for Medical Humanities<br>Liu, Yuning; Peking University Health Science Centre, School of Public Health<br>Qu, Xueqi; Peking University Health Science Centre, School of Public Health<br>Gaoshan, Junjian; United Nations Population Fund China Office

**Primary Subject Heading**:	Public health
Secondary Subject Heading:	Nutrition and metabolism, Epidemiology
Keywords:	breastfeeding, EPIDEMIOLOGY, China, Maternal medicine < OBSTETRICS, SOCIAL MEDICINE

SCHOLARONE™
Manuscripts

The association between maternal education and breastfeeding practices in China: a population-based cross-sectional study

Authors: Kun Tang[1],* Hanyu Wang[2],* Shi Hui Tan[3], Tong Xin[4], Yuqi Wang[5], Yuning Liu[6], Xueqi Qu[7], Junjian Gaoshan[8]

*Co-first authors

1 Research Center for Public Health, Tsinghua University, Beijing, China. E-mail: tangkun@post.harvard.edu

2 Institute for Medical Humanities, Peking University Health Science Center, Beijing, China. E-mail: whybest@pku.edu.cn

3 School of Basic Medical Sciences, Peking University Health Science Center, Beijing, China. E-mail: shi.tan@mail.mcgill.ca

4 Department of Global Health, Peking University Health Science Center, Beijing, China. E-mail: xtong.katrina@gmail.com

5 Institute for Medical Humanities, Peking University Health Science Center, Beijing, China. E-mail: 18811761299@163.com

6 School of Public Health, Peking University Health Science Center, Beijing, China. E-mail: lynpkuhsc@126.com

7 School of Public Health, Peking University Health Science Center, Beijing, China. E-mail: quxueqisnow@163.com

8 United Nations Population Fund China Office. E-mail: j.gaoshan@gmail.com

Corresponding Author: Dr. Kun Tang

Address: Tsinghua University, Haidian District, Beijing 100191, P.R. China

Telephone Number: 86-10-82805951

E-mail Address：tangkun@post.harvard.edu

Key Words: Breastfeeding, maternal education, income, social determinants, China

Word counts: 300 for the abstract and 3,181 for the main body of text

For peer review only

Abstract:

Objective:

To investigate the association between maternal education and breastfeeding in the Chinese population, with the consideration of household income and health-seeking behaviors.

Design:

A nationally representative population-based cross-sectional study

Setting:

77 counties from 12 geographically distinct regions in China

Participants:

10,408 mothers with children from 0-12 months of age, aged 15-53 years old (Mean: 29.15, SD: 5.11) were classified into primary school and below group (n=781), middle school group (n=3,842), high school/vocational school group (n=1,990), college and above group (n=3,795), according to their highest education.

Outcomes:

Five breastfeeding outcomes, including early initiation of breastfeeding, exclusive breastfeeding under 6 month, predominant breastfeeding under 6 months, current breastfeeding, and children ever breastfed, were calculated based on the standardized questionnaire from the WHO and Wellstart International's toolkit for monitoring and evaluating breastfeeding activities.

Results:

A higher level of maternal education is positively associated with early initiation of breastfeeding (RR: 1.22, 95% CI: 1.12-1.30 for the highest education group) and was inversely associated with exclusive breastfeeding (RR: 0.59, 95% CI: 0.38-0.88 for the highest education group). Stratified by household income, a positive association with early initiation was observed only in the group with the highest household income (adjusted prevalence: 44.95%, 95% CI: 40.65%-49.25% in the lowest education group versus 78.39%, 95% CI: 76.25%-

For peer review only

80.52% in the highest education group), and an inverse association with exclusive breastfeeding was found in both low and high household income groups.

Conclusions:

Mothers with a higher education were more likely to early initiate breastfeeding while less likely to exclusively breastfeed their babies. Routine and successful nursing are crucial for the health of infants and largely influenced by social factors such as maternal education observed in the present study. Future public health interventions on breastfeeding promotion should take into consideration the issues related to educational level of mothers.

Strengths and limitations of this study

The present study is a nationally representative population-based study with large sample size and is based on the first survey conducted in China to specifically explore the social determinants of breastfeeding.

This is the first study to investigate the relationship between maternal education and comprehensive breastfeeding patterns in a national scale in China.

We specified breastfeeding indicators and explored the associations separately and stratified by possible influential factors.

Maternal education in this study represents formal education gained through schooling and it may not reflect the actual health literacy of the mothers

Information was based on self-report in the survey and feeding practices were determined using the 'last-24-hour method' which tends to overestimate the prevalence of exclusive breastfeeding as compared to the 'since birth method'

Funding

This study was supported by Bill & Melinda Gates Foundation and China Development Research Foundation.

For peer review only - http://bmjopen.bmj.com/site/about/guidelines.xhtml

For peer review only

INTRODUCTION

Extensive research has reported diverse and compelling advantages of breastfeeding for infants, mothers, families, and society.[1] Breastfeeding has become more common in the last few years because of numerous interventions conducted at multiple levels from the communities, hospitals, and the society in the whole, but the rates of breastfeeding still remain below optimal levels as recommended by the world health organization in many countries.[2 3] According to a recent, cross-sectional report of 55 counties from 30 provinces in China, the prevalence of ever breastfeeding was 79.6%, and only 20.8% of 14,539 children surveyed were exclusively breastfed at 6 months.[4]

WHO recognizes almost all mothers as biologically capable to breastfeed, except in a small number of health conditions, that use of breast milk substitutes is justifiable.[5] Breastfeeding practices, however, are not merely biological issues but are also related to health behavior and influenced by multifactorial determinants, including historical, socioeconomic, and cultural factors.[6] Women's breastfeeding practices are also affected by personal attributes such as age, weight, education, and confidence.[6] In China, breastfeeding practice face unique challenges, due to its diverse cultural values, political system, and historical perception of breastfeeding.[7 8]

Education status of the mother has been identified as an important determinant for child welfare, survival, and health.[9-11] Skafida et al found that maternal education was a more informative predictor of breastfeeding initiation than occupation.[12] Although maternal education and breastfeeding practices have been studied extensively and most of the previous literature reported a positive association between maternal education and breastfeeding practices, the positive association may not be true in all context.[13-17] Studies conducted in Nepal,

For peer review only - http://bmjopen.bmj.com/site/about/guidelines.xhtml

United States, and Italy showed that higher maternal education was related to better breastfeeding practices.[13-15] while studies in Ethiopia and Bangladesh observed a negative association of breastfeeding and maternal education.[16 17]

Most of the studies conducted in China explored the effect of maternal education on breastfeeding together with other socioeconomic factors. The results across studies were different and sometimes reached the opposite conclusion.[18] Using the data from 12 regions in China, the aim of this study is to investigate the association between maternal education and breastfeeding, taking into account household income and health-seeking behaviors. The findings of this study contribute to the growing body of evidence on social determinants of breastfeeding and have the potential to provide evidence for related interventions and policies.

METHODS

Study design and participants

This population-based study was conducted in 12 geographically defined regions in China from July 2017 to January 2018. Multi-stage sampling technique was adopted for selection participants. In the first stage, population proportionate sampling (PPS) method was applied, in consideration of the population structure and the status of maternal and child health and of socio-economic development. The selection of 12 study sites was made carefully, aiming to maximize geographic diversity (including northern, southern, eastern and western regions with very different climate and cultures), socioeconomic diversity (including affluent cities and impoverished inland rural areas, as well as cities of different population sizes) and taking into account past maternal and child health situation (i.e. the maternal mortality rates), immigration population proportion, population stability, local commitment and capacity in order to provide a balanced and representative sampling. In the end, 4 megacities (Beijing in North China, Nanning of Guangxi Province in South Central China, Hefei of Anhui province and Nanjing of Jiangsu province in East China), 4 medium-sized cities (Longyan of Fujian

For peer review only - http://bmjopen.bmj.com/site/about/guidelines.xhtml

province in East China, Liaoyuan of Jilin province in Northeast China, Honghe of Yunnan province and Luzhou of Sichuan province in Southwest China), 2 countryside areas (Zhengding County of Hebei province in North China and Jia County of Henan province in Central China), and 2 underdeveloped rural areas (Tailai county of Heilongjiang province in Northeast China and Ledu county of Qinghai province in Northwest China) were selected. In the second stage, one district or county was chosen in the 12 regions by simple random sampling method. In the third stage, simple sampling method was implemented to select 4-8 communities or villages from the chosen district or county in the 12 regions. A survey team, 5 full-time staff with medical qualifications and field experience, was established in each of the 12 regions. Mothers whose children were under 12 months were eligible for this study. To encourage involvement, incentives, such as shopping cards and simple household commodities, were prepared for each participant. By the end, 10,408 mothers from 77 counties were recruited for the present study. After registration and obtaining informed consent, trained health staff with smartphone-based questionnaire collected participants' information. The questionnaire asked for mothers' socio-demographic information, socio-economic information, breastfeeding behavior, and breastfeeding related environment. Over 90% of participants completed the entire questionnaire. The study has been approved by the Institutional Review Boards at Peking University Health Science Center and the China National Center for Disease Control. All the participants included in this study provided informed consent.

For peer review only

Variable definitions

Maternal education is the main exposure in the study. In the baseline survey, maternal education was classified into eight categories, including no formal school, not graduated from primary school, primary school, middle school, high/vocational school, associate/junior college, university, and postgraduate and above. In the present study, those with no formal school education, dropped out from primary school, or those with primary school education only were classified into the 'Primary School and Below' group; those with middle school education

For peer review only - http://bmjopen.bmj.com/site/about/guidelines.xhtml

were classified as the 'Middle School' group; those with high/vocational school education were classified as the 'High/Vocational School' group; and those with an education of associate/junior college, university, postgraduate and above were classified as the 'College and Above' group. This categorization enabled balanced population in each group and made the results interpretable because primary school and below could be regarded as practically illiterate and those with college and above education could be seen as having high education in China.

Four breastfeeding outcome variables including early initiation of breastfeeding (EIB), exclusive breastfeeding under 6 months (EBF), predominant breastfeeding under 6 months (PBF), and children ever breastfed (Ever BF), were analyzed. EIB and ever BF were defined according to World Health Organization indicators.[19] The final calculation of EIB prevalence and ever BF prevalence was conducted among mothers with children from 0-12 months of age. EIB prevalence was defined as proportion of mothers with children born in the last 12 months who were put to the breast within an hour after birth. Ever BF prevalence was defined as proportion of children born in the last 12 months who were ever breastfed. EBF and PBF were defined and evaluated according to Wellstart International's toolkit for monitoring and evaluating breastfeeding activities,[20] using a 24-hour recall methodology. Mothers were asked to recall the food they fed their children in the last 24 hours, and the final calculation of EBF prevalence and PBF prevalence was conducted among mothers with children from 0-6 months. EBF prevalence was defined as proportion of infants 0–6 months of age who were fed exclusively breast milk. PBF prevalence was defined as proportion of infants 0–6 months of age who were predominantly breastfed, which mainly comprised of children who were fed by breast milk and water.[20] Furthermore, to explore mothers' current breastfeeding situation, the present study also implemented the indicator of current breastfeeding (CBF) prevalence, defined as any breastfeeding in the last 24 hours of children less than 12 months of age.[20]

For peer review only - http://bmjopen.bmj.com/site/about/guidelines.xhtml

For peer review only

Mother-infant indicators and family socio-economic status were the two categories of covariates incorporated in the present study. Mother-infant indicators included maternal age, infant age, pre-pregnancy BMI, gestational age, infant birth weight, infant sex, parity, and delivery method. The information of maternal age, gestational age, infant birth weight, infant sex, parity and delivery methods were collected based on mothers' self-reported answers. Infant age was calculated by infants' birth date and survey date. Pre-pregnancy BMI was calculated based on mothers' self-reported weight and height before pregnancy. Apart from maternal education, family socio-economic status includes: household income, residency status, and maternal occupation. These covariates were also collected based on participants' self-reported answers. In the baseline survey, household income was a continuous variable. In the present study, household income was classified into ≤40,000 Yuan (1 USD≈6.33 Chinese Yuan); 40,001-80,000 Yuan; and ＞80,000 Yuan. Occupation was categorized as agriculture and related workers, factory workers, white-collar, and others. Residency status was classified as local or migrant, according to whether the mother lives at the place of residence. Health-seeking behaviors, including considering breastfeeding before pregnancy, attending postpartum mother support group, and receiving breastfeeding education during the perinatal period, were analyzed in the present study.

Data analysis

Descriptive analyses were used to illustrate the basic demographic, socioeconomic and lifestyle characteristics in different maternal education groups. The data of mothers who did not complete the questionnaires as required were excluded from multivariable analyses. The association between maternal education and breastfeeding outcomes was analyzed using logistic regression model. Because the prevalence of the outcomes was high, odds ratio tended to overestimate the risk ratio. Thus, corrected risk ratio was calculated by the widely-used methods proposed by Zhang and Yu.[21] All the risk ratios were presented with 95% confidence interval. Primary school and uneducated group was chosen to be reference groups in all models. Two models were fitted: 1) unadjusted; 2) adjusted for maternal age, infant birth weight, infant sex, parity, delivery method,

For peer review only - http://bmjopen.bmj.com/site/about/guidelines.xhtml

household income, region (super city/urban/rural/poor rural), residency status (local/migrant), paternal education and maternal occupation. The adjusted variables were chosen by reviewing relevant literature and according to the social determinants of breastfeeding framework.[6]

To further understand the relationship between maternal education and early initiation of breastfeeding and exclusive breastfeeding, the adjusted prevalence of early initiation of breastfeeding and exclusive breastfeeding in different maternal education groups was calculated after stratification by household income. The adjusted prevalence of health-seeking behaviors, including considering breastfeeding before pregnancy, attending postpartum mother support group, and receiving breastfeeding education were also calculated in different education groups. The adjusted prevalence was based on logistic regression and the detailed method was described elsewhere.[22] All the analyses were conducted using SAS version 9.4 (SAS Institute, Cary, North Carolina, USA).

For peer review only

Patient involvement

Patients were not involved in setting the research questions or planning the study. Investigators do not know the identity of study participants.

RESULTS

Table 1 shows the baseline characteristics of the study population by maternal education. Of all 10,408 participants included, 7.50% had the highest education at primary school or below, 36.91% had middle school education, 19.12% had high/vocational school education, and 36.46% had college education and above. The mean maternal ages for those whose highest education was primary school and below, middle school, high/vocational school, and college and above were 28.76 (SD=6.68), 28.41 (SD=5.21), 28.26 (SD=4.78), and 30.36 (SD=4.51), respectively. The mean infant age (in days) for mothers whose highest education was

For peer review only - http://bmjopen.bmj.com/site/about/guidelines.xhtml

primary school and below, middle school, high/vocational school, and college and above were 186.50 (SD=106.82), 182.11 (SD=104.48), 178.29 (SD=105.82), and 173.10 (SD=105.52), respectively. The distributions of pre-pregnancy BMI, gestational age, infant birth weight, and infant sex were similar across all maternal education groups. Women with a primary school and below education tended to be multiparous, had vaginal delivery method, lived in urban and poor rural areas, and had lower household income. Women who had primary school education and/or below were more likely to hold agricultural jobs (57.89%) and were less likely to be white-collar (11.77%). Women whose educational level were college and above were more likely to be migrants (46.35%) compared to women of other educational levels.

The overall EIB, CBF, Ever BF, EBF, and PBF rates were 71.80%, 86.98%, 97.47%, 15.40%, and 36.37%, respectively. Detailed breastfeeding rates in mothers with different educational levels could be found at the end of *Table 1.*

Table 2 presents the relationship between breastfeeding practices and maternal education. No significant association was found between CBF and maternal education. Compared to the primary school and below group, other groups showed a greater risk ratio of early initiation and ever breastfeeding. Compared to the primary school and below group, other groups had a smaller risk ratio of exclusive and predominant breastfeeding. After adjusting for possible confounders, the significant association between maternal education and breastfeeding outcomes was only found in early initiation of breastfeeding and exclusive breastfeeding. The detailed RRs and 95% CIs could be referred in Table 2.

Figure 1 presents the adjusted prevalence of early initiation of breastfeeding in different maternal education groups, stratified by household income. The association between maternal education and early initiation of breastfeeding was only significant in those with household income >80,000 Yuan. In this group, a lower

For peer review only - http://bmjopen.bmj.com/site/about/guidelines.xhtml

prevalence of early initiation of breastfeeding (44.95%, 95% CI: 40.65%-49.25%) was observed in those with primary school education and below compared to those with college education and above (78.39%, 95% CI: 76.25%-80.52%)).

Figure 2 presents the adjusted prevalence of exclusive breastfeeding in different maternal education groups, stratified by household income. There was no significant association between maternal education and exclusive breastfeeding prevalence in those with household income of 40,001-,80,000 Yuan. For those with household income of ≤40,000 Yuan and ＞80,000 Yuan, there was a general negative association between maternal education and incidence of exclusive breastfeeding. For those with household income of ≤40,000 Yuan, a higher prevalence of exclusive breastfeeding was observed in those with primary school education and below (36.54%, 95% CI: 29.93%-43.15%) to compared to those with college education and above (11.61%, 95% CI: 10.58%-12.65%). For those with a household income of >80,000 Yuan, a higher prevalence of exclusive breastfeeding was observed in those with a primary and below education (26.83%, 95% CI: 26.63%-27.03%) compared to in those with a college education (9.69%, 95% CI: 8.14%-11.23%).

Figure 3 presents the adjusted prevalence of health-seeking behaviors, including considering breastfeeding before pregnancy, attending mother support group postpartum, and receiving breastfeeding education. There was a general positive association between health-seeking behaviors and maternal education across these three factors. A lower prevalence of considering breastfeeding before pregnancy was observed in those with primary school education and below (28.35%, 95% CI: 25.98%-30.73%) compared to those with college education and above (53.92%, 95% CI: 52.09%-55.75%). A higher prevalence of attending mother support group postpartum was observed 1in those with primary school education and below (10.09%, 95% CI: 9.20%-10.98%) compared to those with college education and above (56.03%, 95% CI: 54.55%-57.51%). A lower prevalence of receiving breastfeeding education was also observed in those with primary school education

For peer review only - http://bmjopen.bmj.com/site/about/guidelines.xhtml

and below (32.65%,95% CI: 30.71%-34.59%) compared to those with college education and above (66.47%, 95% CI: 65.16%-67.78%).

DISCUSSION

Several important findings are illustrated in the present study. First, maternal education is inversely related to exclusive breastfeeding. Second, maternal education is positively related to early initiation of breastfeeding in the high household income group. Third, after stratification by income, the patterns of association between maternal education and breastfeeding indicators differ in different income groups. The positive relationship between maternal education and early initiation of breastfeeding is observed in the group with high household income. The inverse relationship between maternal education and exclusive breastfeeding exists in low and high household income groups, and the association is stronger in low household income group.

This study is based on a large, national representative, population-based survey. Large sample size ensures high precision of the findings. The associations observed in the present study accorded with and contributed to the development of social determinants of breastfeeding framework.[6] This is one of the few studies focused mainly on the association between maternal education and breastfeeding practices in China. We specified breastfeeding indicators and explored the associations separately and stratified by possible influential factors. Quantitative data were used to analyze health-seeking behavior. Admittedly, there are some potential limitations of the present study. First, causal inference between maternal education and breastfeeding practices is limited due to the study being cross-sectional. Further prospective study is needed in order to establish a causal relationship. Second, maternal education in this study represents formal education gained through schooling and it may not reflect the actual health literacy of the mothers. Third, feeding practices were determined using the 'last-24-hour method' which tends to overestimate the prevalence of EBF as compared to the 'since birth method' [23]and the information used in the analysis was all based on mothers' self-report ,

For peer review only - http://bmjopen.bmj.com/site/about/guidelines.xhtml

which might introduce recall biases. Last, one of the potential limitations of the present study is that we didn't survey the number of current births among the mothers. As the association between maternal education and infant breastfeeding practices might differ in twins and singletons, this could affect the accuracy of the present findings.

In contrast to early initiation of breastfeeding, maternal education is inversely related to exclusive breastfeeding. This is consistent with previous studies conducted in China.[18] A systematic review conducted in China exploring the relationship between maternal education and breastfeeding found a negative association between breastfeeding indicators and maternal education. The review speculated that maternal leave, occupation, and incorrect traditional perceptions contributed to the negative association.[18] One of the traditional perceptions was that breastfeeding would change mothers' body shape and have negative impact on mothers' health.[24] Nevertheless, our finding is opposite to those in the developed countries.[14 15] In China, highly educated mothers are usually working full time in responsible and formal positions. The maternity leave regulated by legislation is 98 days in China,[25] which is not enough to guarantee the six-month exclusive breastfeeding time as recommended by WHO. It is also equally difficult for mothers who have already returned to work to continue breastfeeding due to lack of related supporting facilities and working pressure.[26] Another possible explanation is that highly educated mothers tend to have higher household income, and thus are able to afford infant formula, which is especially true in China.[27] Due to infant formula advertisement and lack of proper breastfeeding education, use of infant formula is sometimes regarded as healthier and also a symbol of wealth in China.[27 28] Besides, incorrect perceptions in some areas maintain that breastfeeding changes mothers' body shape.[24] Women with higher education generally in live in urban areas and in a relatively higher social class. Women in urban areas and at higher social classes may face a higher social pressure. Thus, they may pay more attention to their own body shape and are more willing to choose infant formula.[29]

For peer review only - http://bmjopen.bmj.com/site/about/guidelines.xhtml

In the present study, positive association between early initiation of breastfeeding and maternal education is observed in those with household income >80,000 Yuan. The positive association between maternal education and early initiation is consistent with studies conducted in the developed countries.[14 15 30] Previous studies in developing countries showed inconsistent results concerning the relationship between maternal education and early initiation of breastfeeding.[13 31-33] A study conducted in Laos found that early initiation of breastfeeding was most prevalent among mothers with higher education[31] and a study in Nepal observed the similar result.[13] A study conducted in Vietnam, however, found that mothers with no education were more likely to initiate breastfeeding.[33] Results from the WHO Global Survey that was conducted mostly in developing countries found an overall negative association between maternal education and early initiation of breastfeeding.[32] The positive association in this study needs to be explored. In the present study, mothers with higher educational attainment tend to exhibit proactive health behaviors, early consideration of feeding methods and more likely to receive breastfeeding education, all of which may foster early initiation of breastfeeding. It has been suggested that women with higher education tend to have stronger social support,[34] and more likely to attend mother support group as observed in this study. Previous study found that social support is an important contributor to early initiation of breastfeeding.[35 36] Furthermore, women with higher education are more likely to live in super cities and have higher household income. There exists a huge disparity of health services between rural and urban areas in China.[37] Thus, the women who live in super cities are more likely to have better access to quality health services. Previous studies found that professional knowledge of nurses and doctors may foster early initiation of breastfeeding.[38 39] A review conducted in India suggested that interventions designed to increase the knowledge and skills of healthcare workers were important to promote early initiation of breastfeeding.[40]

After stratification by income, the patterns of association between maternal education and breastfeeding

For peer review only - http://bmjopen.bmj.com/site/about/guidelines.xhtml

indicators differ in different income groups. The positive relationship between maternal education and early initiation of breastfeeding is only significant in the high household income group. Higher education may promote better breastfeeding knowledge and health behavior.[41] Nevertheless, apart from knowledge, early initiation of breastfeeding require social support and access to healthcare services.[3] Women with lower household income may not have sufficient financial abilities to attain healthcare services. These women are more likely to give birth at home, where initiation of breastfeeding within 1 hour of birth may not be promoted. Mothers may recognize the benefits of delivery at healthcare facilities, yet they may not be able to afford the expensive hospitals stays and professional services. The inverse relationship between maternal education and exclusive breastfeeding was the strongest in low household income group. A possible explanation is that women with lower income and higher education tend to have heavier workload in order to meet the demands of the family, consequently shortened exclusive breastfeeding duration. People with lower income tend to live in rural areas, where health infrastructure and expertise of health workers are less advanced than that in urban areas. Traditional values may also be more prevalent than in the cities, such as routine supplementary feeding with water,[42] giving rise to the associations found in this study.

Associations found in the present study had implications for future public health interventions to promote breastfeeding in China. There are also several related factors to consider when designing such interventions. In the present study, Ever BF rates were much higher than EIB rates, which might partly due to the high caesarean section prevalence in China.[7] Baby Friendly Hospital Initiative covered most of the hospitals with obstetrical department in China and have had positive impacts on breastfeeding practices, but the implementation of this Initiative was highly questionable due to a lack of routine monitoring and evaluation, which might reduce EIB rates.[7 43] Sociocultural norms in China, such as postpartum confinement, were widely practiced, which might also affect mothers' breastfeeding behaviors.[42] In addition, despite a nation-wide melamine milk scandal in 2008, the infant formula marketing was even more prevalent in China [27 28], after the

For peer review only - http://bmjopen.bmj.com/site/about/guidelines.xhtml

Chinese government abolished the International Code of Marketing of Breast-milk Substitutes.[44] All of these suggest that a large public campaign involving multisectoral stakeholders to promote breastfeeding practices is highly needed in China.

Conclusions

A positive association between maternal education and early initiation of breastfeeding and a negative association between maternal education and exclusive breastfeeding were observed in this study, with the consideration of health seeking behavior and household income. Routine and successful breastfeeding, as per The American Academy of Pediatrics recommendation,[1] are crucial for the health of the infant and is largely influenced by social factors. This study underscores the influence of maternal education on breastfeeding practices. Findings from this study contribute to the growing body of research on social determinants of breastfeeding. This study suggests that extensive public health interventions on breastfeeding promotion in China should target on mothers with higher levels of education, especially among those with medium to high incomes. Relevant supportive policies and programs for this group of mothers, including enforcing full-term maternal leave, providing breastfeeding education and counseling, building a mother-friendly workplace, as well as breaking cultural and social norms, should be implemented to create an enabling environment for breastfeeding practice among Chinese mothers.

Contributors

KT and HW contributed to the study concept and design, statistical analysis, results interpretation, and drafting and revision of the manuscript. ST contributed to the study concept and design, drafting and revision of the manuscript. TX contributed to the study concept and design, revision of the manuscript. YW contributed to revision of the manuscript. YL and XQ contributed to the study concept and design and results interpretation. JG contributed to the study concept and design. All authors read and approved the final version of the

For peer review only - http://bmjopen.bmj.com/site/about/guidelines.xhtml

manuscript.

Funding

This study was supported by Bill & Melinda Gates Foundation and China Development Research Foundation.

Competing interests

None declared.

Patient consent

Not required.

Ethics approval

This study was conducted according to the guidelines laid down in the Declaration of Helsinki and all procedures involving human subjects/patients has been approved by the Institutional Review Boards at Peking University Health Science Center and the China National Center for Disease Control. The participants included in this study provided written informed consent.

Data sharing statement

Data are available upon reasonable request

Acknowledgement

We are grateful for Chinese Center For Disease Control And Prevention for providing the data.

References

1. Eidelman AI, Schanler RJ, Johnston M, et al. Breastfeeding and the use of human milk. *Pediatrics* 2012;129(3):e827-e41.
2. Cai X, Wardlaw T, Brown DW. Global trends in exclusive breastfeeding. *International breastfeeding journal* 2012;7(1):12.
3. Victora CG, Bahl R, Barros AJ, et al. Breastfeeding in the 21st century: epidemiology, mechanisms, and lifelong effect. *The Lancet* 2016;387(10017):475-90.
4. Yang Z, Lai J, Yu D, et al. Breastfeeding rates in China: a cross-sectional survey and estimate of benefits of improvement. *The Lancet* 2016;388:S47.
5. World Health Organization. Acceptable medical reasons for use of breast-milk substitutes. 2009
6. Rollins NC, Bhandari N, Hajeebhoy N, et al. Why invest, and what it will take to improve breastfeeding practices? *The Lancet* 2016;387(10017):491-504.
7. Xu F, Qiu L, Binns CW, et al. Breastfeeding in China: a review. *International Breastfeeding Journal*

For peer review only - http://bmjopen.bmj.com/site/about/guidelines.xhtml

2009;4(1):6.
8. Jiang H, Li M, Yang D, et al. Awareness, intention, and needs regarding breastfeeding: findings from first-time mothers in Shanghai, China. *Breastfeeding Medicine* 2012;7(6):526-34.
9. Meyrose A-K, Klasen F, Otto C, et al. Benefits of maternal education for mental health trajectories across childhood and adolescence. *Social Science & Medicine* 2018;202:170-78.
10. Güneş PM. The role of maternal education in child health: Evidence from a compulsory schooling law. *Economics of Education Review* 2015;47:1-16.
11. Desai S, Alva S. Maternal education and child health: Is there a strong causal relationship? *Demography* 1998;35(1):71-81.
12. Skafida V. The relative importance of social class and maternal education for breast-feeding initiation. *Public health nutrition* 2009;12(12):2285-92.
13. Acharya P, Khanal V. The effect of mother's educational status on early initiation of breastfeeding: further analysis of three consecutive Nepal Demographic and Health Surveys. *BMC public health* 2015;15(1):1069.
14. Heck KE, Braveman P, Cubbin C, et al. Socioeconomic status and breastfeeding initiation among California mothers. *Public health reports* 2006;121(1):51-59.
15. Bertini G, Perugi S, Dani C, et al. Maternal education and the incidence and duration of breast feeding: a prospective study. *Journal of pediatric gastroenterology and nutrition* 2003;37(4):447-52.
16. Alemayehu T, Haidar J, Habte D. Determinants of exclusive breastfeeding practices in Ethiopia. *Ethiopian Journal of Health Development* 2009;23(1)
17. Mihrshahi S, Kabir I, Roy SK, et al. Determinants of infant and young child feeding practices in Bangladesh: secondary data analysis of Demographic and Health Survey 2004. *Food and nutrition bulletin* 2010;31(2):295-313.
18. Zhao J, Zhao Y, Du M, et al. Maternal education and breastfeeding practices in China: A systematic review and meta-analysis. *Midwifery* 2017;50:62-71.
19. World Health Organization. Indicators for assessing infant and young child feeding practices: conclusions of a consensus meeting held 6-8 November 2007 in Washington DC, USA: World Health Organization (WHO) 2008.
20. Lung'aho M, Huffman SL, Labbok MH, et al. Tool kit for monitoring and evaluating breastfeeding practices and programs. *Washington, DC: Wellstart International* 1996
21. Zhang J, Kai FY. What's the relative risk?: A method of correcting the odds ratio in cohort studies of common outcomes. *Jama* 1998;280(19):1690-91.
22. Santos CAS, Fiaccone RL, Oliveira NF, et al. Estimating adjusted prevalence ratio in clustered cross-sectional epidemiological data. *BMC Medical Research Methodology* 2008;8(1):80.
23. Khanal V, Lee AH, Scott JA, et al. Implications of methodological differences in measuring the rates of exclusive breastfeeding in Nepal: findings from literature review and cohort study. *BMC pregnancy and childbirth* 2016;16(1):389.
24. Gao Ll, Chan SWc, You L, et al. Experiences of postpartum depression among first-time mothers in mainland China. *Journal of Advanced Nursing* 2010;66(2):303-12.
25. Jia N, Dong X-y, Song Y-p. Paid Maternity Leave and Breastfeeding in Urban China. *Feminist Economics* 2017:1-23.
26. Bai DL, Fong DYT, Tarrant M. Factors associated with breastfeeding duration and exclusivity in mothers returning to paid employment postpartum. *Maternal and child health journal* 2015;19(5):990-99.
27. Kent G. Global infant formula: monitoring and regulating the impacts to protect human health. *International breastfeeding journal* 2015;10(1):6.
28. Tang L, Lee AH, Binns CW, et al. Widespread usage of infant formula in China: a major public health problem. *Birth* 2014;41(4):339-43.

For peer review only - http://bmjopen.bmj.com/site/about/guidelines.xhtml

29. Gottschang SZ. Infant Feeding and the Feminine Body in Urban China. *China urban: Ethnographies of contemporary culture* 2001
30. Meedya S, Fahy K, Kable A. Factors that positively influence breastfeeding duration to 6 months: a literature review. *Women and Birth* 2010;23(4):135-45.
31. Kounnavong S, Pak-Gorstein S, Akkhavong K, et al. Key determinants of optimal breastfeeding practices in Laos. *Food and Nutrition Sciences* 2013;4(10):61.
32. Takahashi K, Ganchimeg T, Ota E, et al. Prevalence of early initiation of breastfeeding and determinants of delayed initiation of breastfeeding: secondary analysis of the WHO global survey. *Scientific reports* 2017;7:44868.
33. Bui QT-T, Lee H-Y, Le AT-K, et al. Trends and determinants for early initiation of and exclusive breastfeeding under six months in Vietnam: results from the Multiple Indicator Cluster Surveys, 2000–2011. *Global health action* 2016;9(1):29433.
34. Heaney CA, Israel BA. Social networks and social support. *Health behavior and health education: Theory, research, and practice* 2008;4:189-210.
35. Patel A, Bucher S, Pusdekar Y, et al. Rates and determinants of early initiation of breastfeeding and exclusive breast feeding at 42 days postnatal in six low and middle-income countries: A prospective cohort study. *Reproductive health* 2015;12(2):S10.
36. Bai DL, Fong DYT, Lok KYW, et al. Relationship between the infant feeding preferences of Chinese mothers' immediate social network and early breastfeeding cessation. *Journal of Human Lactation* 2016;32(2):301-08.
37. Liu M, Zhang Q, Lu M, et al. Rural and urban disparity in health services utilization in China. *Medical care* 2007;45(8):767-74.
38. Balogun OO, Dagvadorj A, Anigo KM, et al. Factors influencing breastfeeding exclusivity during the first 6 months of life in developing countries: a quantitative and qualitative systematic review. *Maternal & child nutrition* 2015;11(4):433-51.
39. Sharma IK, Byrne A. Early initiation of breastfeeding: a systematic literature review of factors and barriers in South Asia. *International breastfeeding journal* 2016;11(1):17.
40. Aguayo VM, Gupta G, Singh G, et al. Early initiation of breastfeeding on the rise in India. *BMJ global health* 2016;1(2):e000043.
41. Adler NE, Boyce T, Chesney MA, et al. Socioeconomic status and health: the challenge of the gradient. *American psychologist* 1994;49(1):15.
42. Raven JH, Chen Q, Tolhurst RJ, et al. Traditional beliefs and practices in the postpartum period in Fujian Province, China: a qualitative study. *BMC Pregnancy and Childbirth* 2007;7(1):8.
43. Tang L, Binns CW, Lee AH, et al. Low prevalence of breastfeeding initiation within the first hour of life in a rural area of Sichuan Province, China. *Birth* 2013;40(2):134-42.
44. China NHaFPCotPsRo. Order No. 17: National health and family planning Commission's decree on deciding to repeal the regulations on the sale of substitutes for breast milk substitutes. 2017

For peer review only

For peer review only - http://bmjopen.bmj.com/site/about/guidelines.xhtml

Table 1 Demographic and social-economic characteristics of the participants

	Maternal Education			
	Primary school and below (N=781)	Middle School (N=3842)	High School/Vocational School (N=1990)	College and above (N=3795)
Mean maternal age, year (SD)	28.76(6.68)	28.41(5.21)	28.26(4.78)	30.36(4.51)
Mean pre-pregnancy BMI, Kg/m^2 (SD)	22.67(4.66)	22.44(4.16)	21.74(4.04)	21.75(4.12)
Mean gestational age, week (SD)	39.02(1.38)	39.03(1.40)	38.94(1.51)	38.98(1.38)
Mean infant birth weight, kg (SD)	3.23(0.47)	3.33(0.59)	3.34(0.67)	3.43(0.75)
Mean infant age, day (SD)	186.50(106.82)	182.11(104.48)	178.29(105.82)	173.10(105.52)
Infant sex, %				
Male	50.67	50.64	52.17	50.08
Female	49.33	49.36	47.83	49.92
Parity, %				
Primiparous	29.70	33.87	43.49	62.06
Multiparous	70.30	66.13	56.51	37.94
Delivery method, %				
Vaginal	65.77	56.72	54.59	57.01
Cesarean section	34.23	43.28	45.41	42.99
Region, %				
Super city	9.70	14.16	30.82	61.55
Urban	43.15	32.08	39.09	27.41
Rural	7.15	25.39	21.09	7.29
Poor rural	40.00	28.37	9.00	3.75
Household income, %				
≤40,000 yuan	61.74	37.44	20.06	11.81
40,000-79,999 yuan	22.39	30.01	26.21	13.82
≥80,000 yuan	3.48	7.84	16.40	38.89
Maternal Occupation, %				
Agriculture related	57.89	33.61	9.37	2.27
Factory workers	11.53	19.95	20.31	8.66
White-collar	11.77	26.74	47.23	73.03
Others[1]	18.81	19.71	23.09	16.04
Resident status, %				
Local	69.70	69.15	59.50	53.65
Migrant	30.30	30.85	40.50	46.35
Breastfeeding practice, %				
Early initiation	64.85	68.24	71.11	77.21
Current BF	87.88	88.53	85.09	86.21
Ever BF	95.52	97.31	97.65	97.93
Exclusive BF (0-6 months)	26.53	15.36	13.64	14.06

[1] Others refer to unidentified occupation, unemployed, and housewives

For peer review only - http://bmjopen.bmj.com/site/about/guidelines.xhtml

For peer review only

Predominant BF (0-6 months)	50.00	39.99	18.44	39.29
Health Seeking Behaviors				
Considered feeding method before pregnancy	28.31	45.35	50.24	55.29
Attended mother support group	8.24	24.40	38.20	49.36
Received breastfeeding education	38.59	47.23	59.13	64.23

Table 2. Relations between breastfeeding practices and maternal education*

	Corrected Unadjusted Risk Ratio (95% CI)			
	Primary school and below	Middle School	High School/Vocational School	College and above
Early initiation	1	1.05(1.00,1.10)	1.10(1.04,1.15)	1.19(1.14,1.23)
Current BF	1	1.01(0.98,1.03)	0.97(0.93,1.00)	0.98(0.95,1.01)
Ever BF	1	1.02(1.01,1.03)	1.02(1.01,1.03)	1.02(1.01,1.03)
Exclusive BF (0-6 months)	1	0.58(0.47,0.72)	0.52(0.40,0.65)	0.53(0.42,0.66)
Predominant BF (0-6 months)	1	0.80(0.70,0.91)	0.57(0.48,0.68)	0.44(0.36,0.52)
	Corrected Adjusted Risk Ratio (95% CI)			
	Primary school and below	Middle School	High School/Vocational School	College and above
Early initiation	1	1.14(1.06,1.22)	1.16(1.06,1.25)	1.22(1.12,1.30)
Current BF	1	0.98(0.91,1.03)	0.94(0.85,1.01)	0.99(0.92,1.05)
Ever BF	1	1.01(0.98,1.03)	1.01(0.97,1.03)	1.02(0.98,1.03)
Exclusive BF (0-6 months)	1	0.53(0.38,0.73)	0.66(0.44,0.96)	0.59(0.38,0.88)
Predominant BF (0-6 months)	1	0.86(0.69,1.05)	0.90(0.7,1.10)	0.78(0.77,0.98)

**Adjusted for maternal age, infant birth weight, infant sex, parity, delivery method, household income, region (super city/urban/rural/poor rural), residency (local/migrant), paternal education, and occupation.*

For peer review only

Figure 1 Adjusted Prevalence* of early initiation in different maternal education groups, stratified by household Income

**After adjusting for maternal age, infant sex, parity, delivery method, paternal education, region (super city/urban/rural/poor rural), residency (local/migrant), occupation*

Figure 2 Adjusted Prevalence* of exclusive breastfeeding in different maternal education groups, stratified by household Income

*After adjusting for maternal age, infant sex, parity, delivery method, paternal education, region (super city/urban/rural/poor rural), residency (local/migrant), occupation

Figure 3 Adjusted Prevalence* of considering feeding method before pregnancy, attending mother support group, receiving breastfeeding education in different maternal education groups*

**After adjusting for maternal age, infant sex, parity, delivery method, paternal education, household income, region (super city/urban/rural/poor rural), residency (local/migrant), occupation.*

For peer review only - http://bmjopen.bmj.com/site/about/guidelines.xhtml

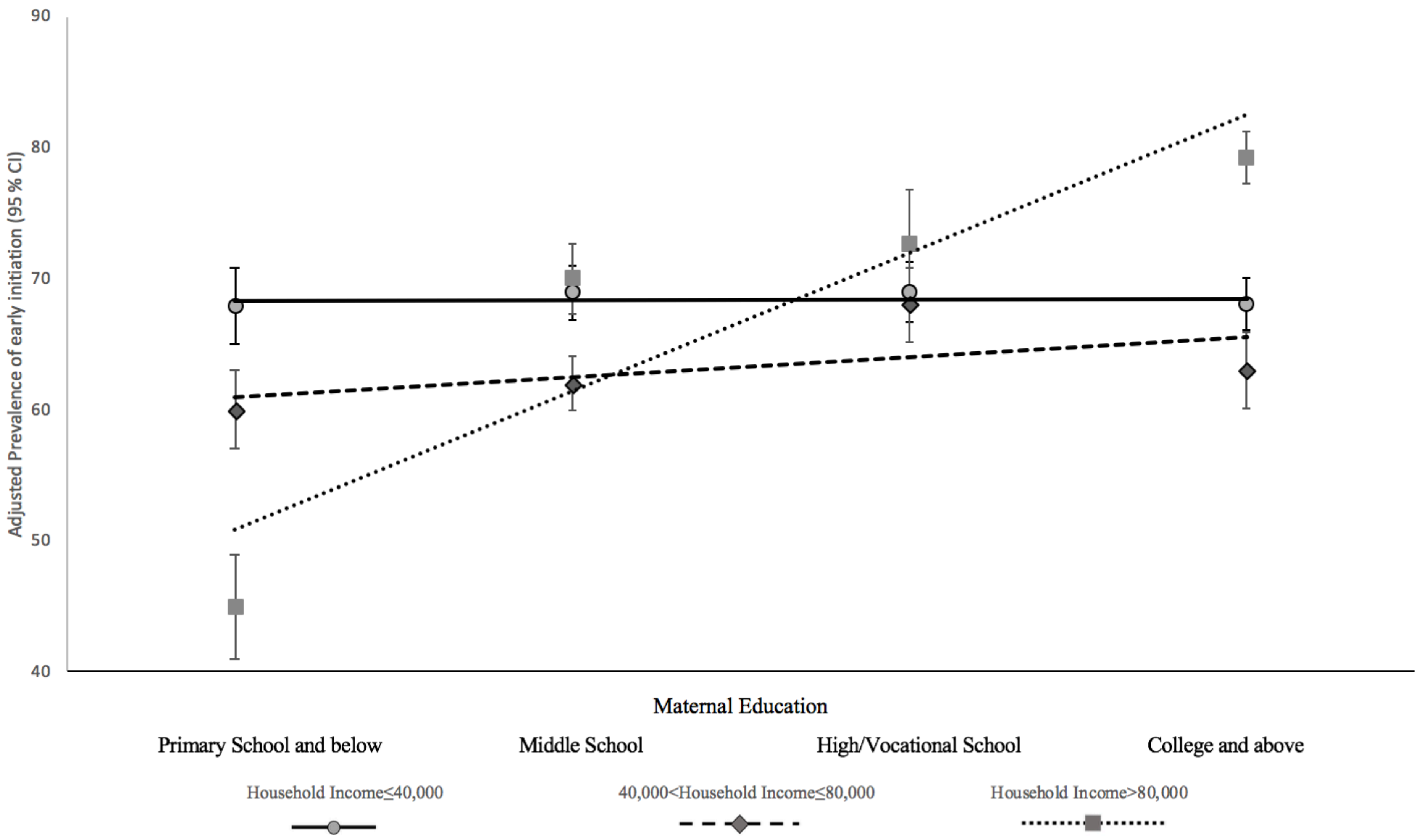
90
80
70
60
50
40
Adjusted Prevalence of early initiation (95 % CI)
Maternal Education
Primary School and below
Middle School
High/Vocational School
College and above
Household Income≤40,000
40,000<Household Income≤80,000
Household Income>80,000

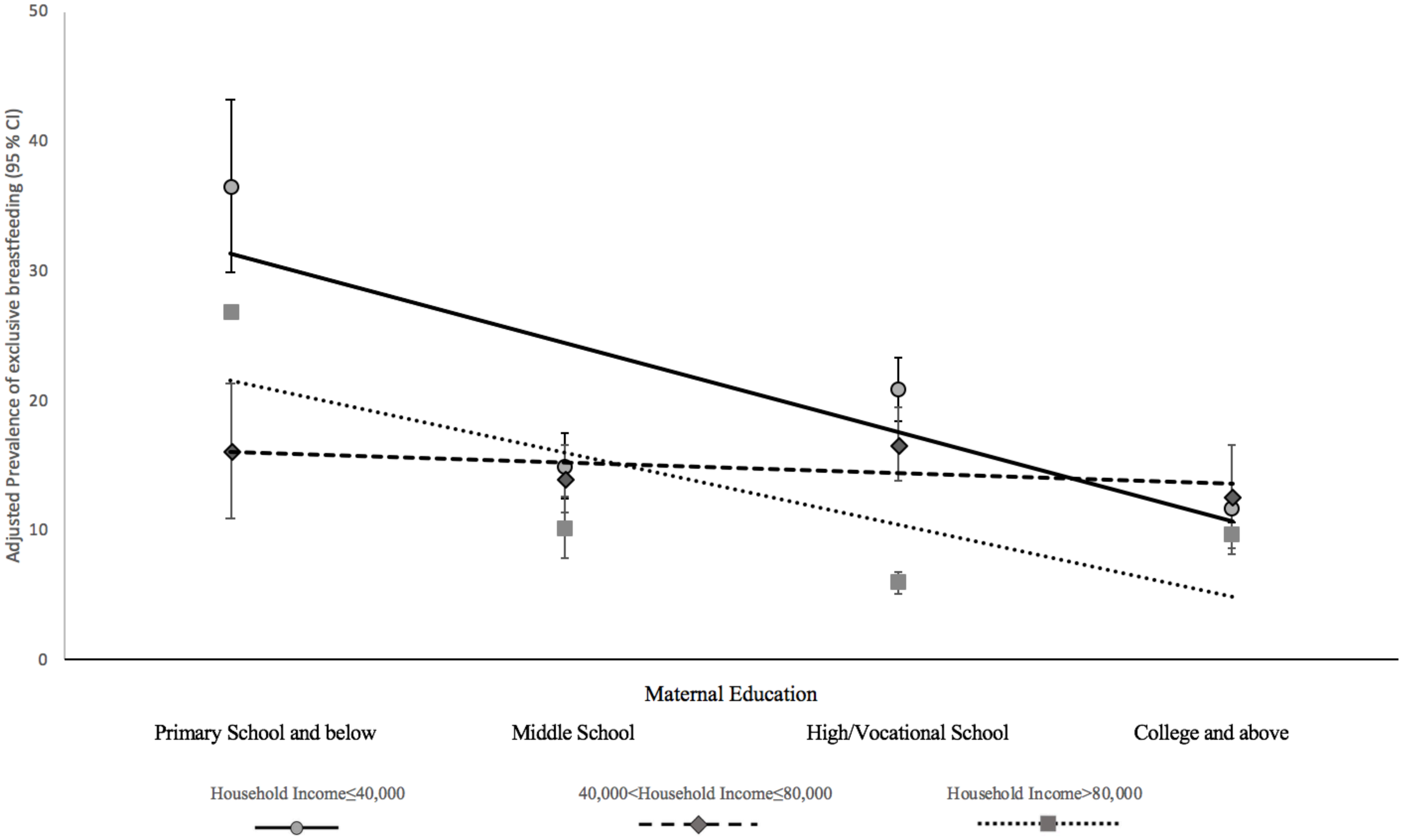
50
40
30
20
10
0
Adjusted Prevalence of exclusive breastfeeding (95 % CI)
Maternal Education
Primary School and below
Middle School
High/Vocational School
College and above
Household Income≤40,000
40,000<Household Income≤80,000
Household Income>80,000

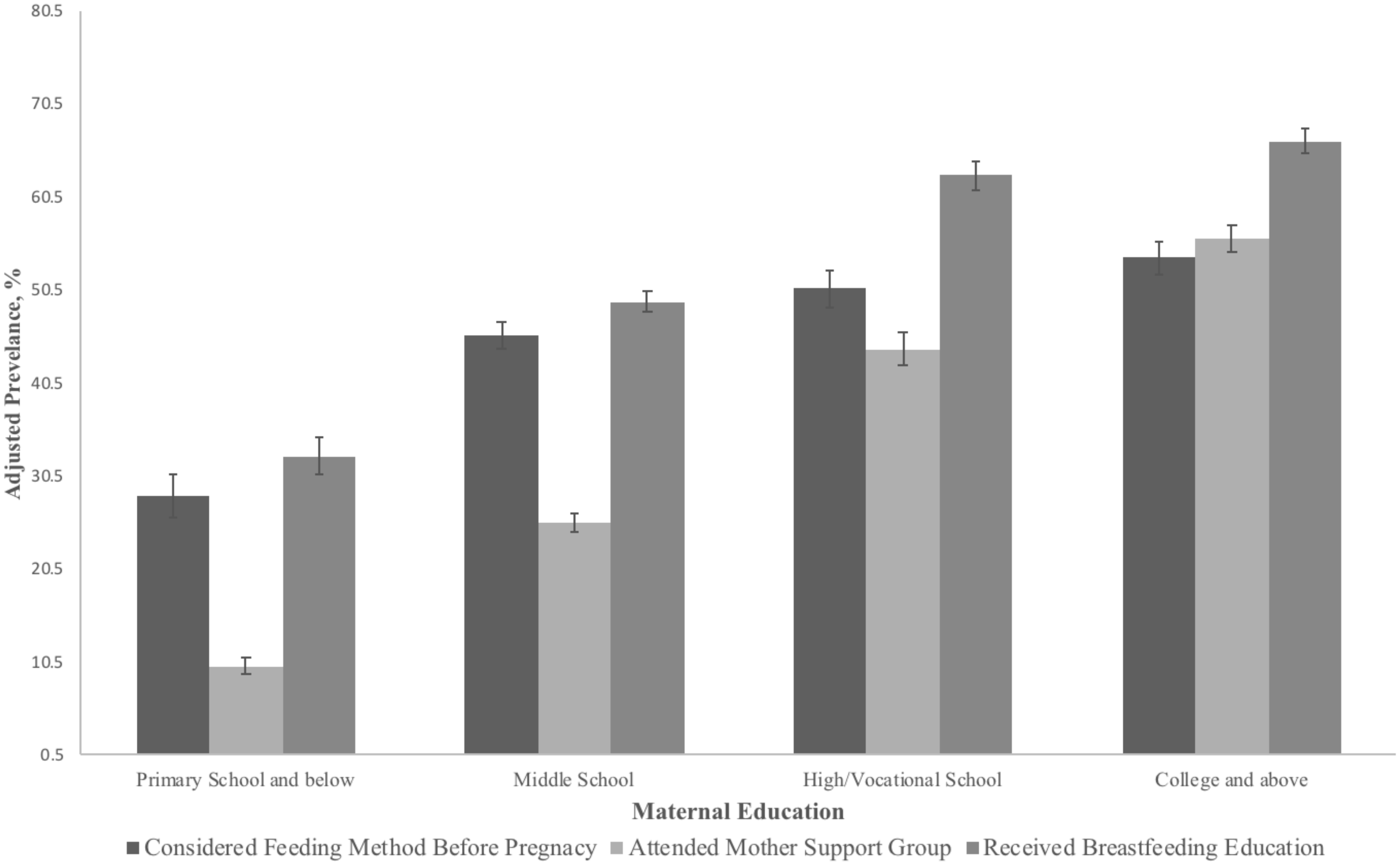
80.5
70.5
60.5
50.5
40.5
30.5
20.5
10.5
0.5
Adjusted Prevelance, %
Primary School and below
Middle School
High/Vocational School
College and above
Maternal Education
Considered Feeding Method Before Pregnacy
Attended Mother Support Group
Received Breastfeeding Education

Reporting checklist for cross sectional study.

Based on the STROBE cross sectional guidelines.

Instructions to authors

Complete this checklist by entering the page numbers from your manuscript where readers will find each of the items listed below.

Your article may not currently address all the items on the checklist. Please modify your text to include the missing information. If you are certain that an item does not apply, please write "n/a" and provide a short explanation.

Upload your completed checklist as an extra file when you submit to a journal.

In your methods section, say that you used the STROBE cross sectional reporting guidelines, and cite them as:

von Elm E, Altman DG, Egger M, Pocock SJ, Gotzsche PC, Vandenbroucke JP. The Strengthening the Reporting of Observational Studies in Epidemiology (STROBE) Statement: guidelines for reporting observational studies.

		Reporting Item	Page Number
Title	#1a	Indicate the study's design with a commonly used term in the title or the abstract	1
Abstract	#1b	Provide in the abstract an informative and balanced summary of what was done and what was found	2-3
Background / rationale	#2	Explain the scientific background and rationale for the investigation being reported	4
Objectives	#3	State specific objectives, including any prespecified hypotheses	5
Study design	#4	Present key elements of study design early in the paper	5
Setting	#5	Describe the setting, locations, and relevant dates, including periods of recruitment, exposure, follow-up, and data collection	5-6
Eligibility criteria	#6a	Give the eligibility criteria, and the sources and methods of selection of participants.	5-6

For peer review only

	#7	Clearly define all outcomes, exposures, predictors, potential confounders, and effect modifiers. Give diagnostic criteria, if applicable	6-7
Data sources / measurement	#8	For each variable of interest give sources of data and details of methods of assessment (measurement). Describe comparability of assessment methods if there is more than one group. Give information separately for for exposed and unexposed groups if applicable.	6-8
Bias	#9	Describe any efforts to address potential sources of bias	6-8
Study size	#10	Explain how the study size was arrived at	5-6
Quantitative variables	#11	Explain how quantitative variables were handled in the analyses. If applicable, describe which groupings were chosen, and why	6-8
Statistical methods	#12a	Describe all statistical methods, including those used to control for confounding	8-9
	#12b	Describe any methods used to examine subgroups and interactions	8-9
	#12c	Explain how missing data were addressed	8-9
	#12d	If applicable, describe analytical methods taking account of sampling strategy	8-9
	#12e	Describe any sensitivity analyses	8-9
Participants	#13a	Report numbers of individuals at each stage of study—eg numbers potentially eligible, examined for eligibility, confirmed eligible, included in the study, completing follow-up, and analysed. Give information separately for for exposed and unexposed groups if applicable.	5-6
	#13b	Give reasons for non-participation at each stage	5-6
	#13c	Consider use of a flow diagram	5-6
Descriptive data	#14a	Give characteristics of study participants (eg demographic, clinical, social) and information on exposures and potential confounders. Give information separately for exposed and unexposed groups if applicable.	5-6
	#14b	Indicate number of participants with missing data for each variable of interest	5-6

For peer review only

Outcome data	#15	Report numbers of outcome events or summary measures. Give information separately for exposed and unexposed groups if applicable.	8
Main results	#16a	Give unadjusted estimates and, if applicable, confounder-adjusted estimates and their precision (eg, 95% confidence interval). Make clear which confounders were adjusted for and why they were included	9-11
	#16b	Report category boundaries when continuous variables were categorized	9-11
	#16c	If relevant, consider translating estimates of relative risk into absolute risk for a meaningful time period	9-11
Other analyses	#17	Report other analyses done—e.g., analyses of subgroups and interactions, and sensitivity analyses	9-10
Key results	#18	Summarise key results with reference to study objectives	12
Limitations	#19	Discuss limitations of the study, taking into account sources of potential bias or imprecision. Discuss both direction and magnitude of any potential bias.	12
Interpretation	#20	Give a cautious overall interpretation considering objectives, limitations, multiplicity of analyses, results from similar studies, and other relevant evidence.	13-15
Generalisability	#21	Discuss the generalisability (external validity) of the study results	13-15
Funding	#22	Give the source of funding and the role of the funders for the present study and, if applicable, for the original study on which the present article is based	17

The STROBE checklist is distributed under the terms of the Creative Commons Attribution License CC-BY. This checklist was completed on 10. December 2018 using http://www.goodreports.org/, a tool made by the EQUATOR Network in collaboration with Penelope.ai

For peer review only

BMJ Open

The association between maternal education and breastfeeding practices in China: a population-based cross-sectional study

Journal:	*BMJ Open*
Manuscript ID	bmjopen-2018-028485.R2
Article Type:	Research
Date Submitted by the Author:	23-May-2019
Complete List of Authors:	Tang, Kun; Tsinghua University, Research Center for Public Health<br>Wang, Hanyu; Peking University Health Science Centre, Institute for Medical Humanities<br>Tan, Shi Hui; Peking University Health Science Centre, School of Basic Medical Sciences<br>Xin, Tong; Peking University Health Science Centre, Department of Global Health<br>Qu, Xueqi; Peking University Health Science Centre, School of Public Health<br>Tang, Tianyu; Tsinghua University, Research Center for Public Health<br>Wang, Yuqi; Peking University Health Science Centre, Institute for Medical Humanities<br>Liu, Yuning; Peking University Health Science Centre, School of Public Health<br>Gaoshan, Junjian; United Nations Population Fund China Office
**Primary Subject Heading**:	Public health
Secondary Subject Heading:	Nutrition and metabolism, Epidemiology
Keywords:	breastfeeding, EPIDEMIOLOGY, China, Maternal medicine < OBSTETRICS, SOCIAL MEDICINE

SCHOLARONE™
Manuscripts

The association between maternal education and breastfeeding practices in China: a population-based cross-sectional study

Authors: Kun Tang[1],* Hanyu Wang[2],* Shi Hui Tan[3], Tong Xin[4], Xueqi Qu[5], Tianyu Tang[6], Yuqi Wang[7], Yuning Liu[8], , Junjian Gaoshan[9]

*Co-first authors

[1] Research Center for Public Health, Tsinghua University, Beijing, China. E-mail: tangkun@post.harvard.edu

[2] Institute for Medical Humanities, Peking University Health Science Center, Beijing, China. E-mail: whybest@pku.edu.cn

[3] School of Basic Medical Sciences, Peking University Health Science Center, Beijing, China. E-mail: shi.tan@mail.mcgill.ca

[4] Department of Global Health, Peking University Health Science Center, Beijing, China. E-mail: xtong.katrina@gmail.com

[5] School of Public Health, Peking University Health Science Center, Beijing, China. E-mail: quxueqisnow@163.com

[6] Research Center for Public Health, Tsinghua University, Beijing, China. E-mail: tianyu.tang@outlook.com

[7] Institute for Medical Humanities, Peking University Health Science Center, Beijing, China. E-mail: 18811761299@163.com

[8] School of Public Health, Peking University Health Science Center, Beijing, China. E-mail: lynpkuhsc@126.com

[9] United Nations Population Fund China Office. E-mail: j.gaoshan@gmail.com

Corresponding Author: Dr. Kun Tang

Address: Tsinghua University, Haidian District, Beijing 100191, P.R. China

Telephone Number: 86-10-82805951

E-mail Address：tangkun@post.harvard.edu

Keywords: Breastfeeding, maternal education, income, social determinants, China

Word count: 300 for the abstract and 3,979 for the main body of text

For peer review only - http://bmjopen.bmj.com/site/about/guidelines.xhtml

Abstract:

Objective:

To investigate the association between maternal education and breastfeeding in the Chinese population, with a consideration of household income and health-seeking behaviors.

Design:

A nationally representative population-based cross-sectional study

Setting:

77 counties from 12 geographically distinct regions in China

Participants:

10,408 mothers with children from 0-12 months of age, aged 15-53 years old (Mean: 29.15, SD: 5.11) were classified into primary school or below group (n = 781), middle school group (n = 3,842), high school/vocational school group (n=1,990), college or above group (n=3,795), according to their highest completed education.

Outcomes:

Five breastfeeding outcomes, including early initiation of breastfeeding, exclusive breastfeeding under six months, predominant breastfeeding under six months, current breastfeeding, and children ever breastfed, were calculated based on the standardized questionnaire from the WHO and Wellstart International's toolkit for monitoring and evaluating breastfeeding activities.

Results:

The prevalence of early initiation of breastfeeding and exclusive breastfeeding was 64.85% and 26.53% for mothers with primary school or below education, respectively. A higher level of maternal education was positively associated with early initiation of breastfeeding (RR: 1.22; 95% CI: 1.12-1.30) and was inversely associated with exclusive breastfeeding (RR: 0.59; 95% CI: 0.38-0.88). Stratified by household income, a

For peer review only

For peer review only - http://bmjopen.bmj.com/site/about/guidelines.xhtml

positive association with early initiation was observed only in the group with the highest household income and an inverse association with exclusive breastfeeding was found in both low and high household income groups.

Conclusions:

Mothers with a higher education were more likely to initiate early breastfeeding when they were also from a high-income household while also being less likely to exclusively breastfeed their babies. Routine and successful nursing are crucial for the health of infants and is largely influenced by social factors (maternal education), observed in the present study. Future public health interventions to promote breastfeeding should consider the issues related to the educational level of mothers.

Strengths and limitations of this study

- The present study is a nationally representative population-based study with a large sample size, and is based on the first survey conducted in China to specifically explore the social determinants of breastfeeding.
- This is the first study to investigate the relationship between maternal education and comprehensive breastfeeding patterns on a national scale in China.
- We specified breastfeeding indicators and explored the associations separately and stratified by possible influential factors.
- Maternal education in this study represents the formal education gained through schooling and it may not reflect the actual health literacy of the mothers.
- Information was based on self-report in the survey and feeding practices were determined using the ‘last-24-hour method’, which tends to overestimate the prevalence of exclusive breastfeeding as compared to the ‘since birth method.’

Funding

For peer review only - http://bmjopen.bmj.com/site/about/guidelines.xhtml

This study was supported by Bill & Melinda Gates Foundation and China Development Research Foundation.

INTRODUCTION

Extensive research has reported the diverse and compelling advantages of breastfeeding for infants, mothers, families, and society.[1] Breastfeeding has become more common in the last few years because of numerous interventions conducted at multiple levels from the communities, hospitals, and society as a whole, but the breastfeeding rates remain below optimal levels recommended by the World Health Organization (WHO) in many countries.[2 3] According to a recent cross-sectional report of 55 counties from 30 provinces in China, the prevalence of if a mother ever breastfeed was 79.6%, and only 20.8% of 14,539 children surveyed were exclusively breastfed at 6 months.[4]

WHO recognizes almost all mothers as biologically capable to breastfeed, except in a small number of health conditions, and that the use of breast milk substitutes is justifiable.[5] Breastfeeding practices, however, are not merely biological issues but are also related to health behaviors and are influenced by multifactorial determinants, including historical, socioeconomic, and cultural factors.[6] Women's breastfeeding practices are also affected by personal attributes, such as age, weight, education, and confidence.[6] In China, breastfeeding practices face unique challenges, due to China's diverse cultural values, political system, and historical perception of breastfeeding.[7 8]

The education status of the mother has been identified as an important determinant for child welfare, survival, and health.[9-11] Skafida et al. found that maternal education was a more informative predictor of breastfeeding initiation than occupation.[12] Although maternal education and breastfeeding practices have been studied

For peer review only - http://bmjopen.bmj.com/site/about/guidelines.xhtml

For peer review only

extensively and most of the previous literature reported a positive association between maternal education and breastfeeding practices, the positive association may not be accurate in all contexts.[13-17] Studies conducted in Nepal, the United States, and Italy showed that higher maternal education level was related to better breastfeeding practices.[13-15] However, studies in Ethiopia and Bangladesh observed a negative association between breastfeeding and maternal education.[16 17]

Most of the previous studies conducted in China explored the effect of maternal education on breastfeeding, together with other socioeconomic factors. The results across studies were different and sometimes reached opposing conclusions.[18] Using data from 12 regions in China, the aim of this study is to investigate the association between maternal education and breastfeeding, taking into account household income and health-seeking behaviors. The findings of this study contribute to the growing body of evidence on the social determinants of breastfeeding and have the potential to provide evidence for related interventions and policies.

METHODS

Study design and participants

This population-based study was conducted in 12 geographically defined regions in China from July 2017 to January 2018. Multi-stage sampling technique was adopted for the selection of the participants. In the first stage, population proportionate sampling (PPS) method was applied, due to the population structure and the status of maternal and child health and socioeconomic development. The selection of the 12 study sites was made carefully, aiming to maximize geographic diversity (including northern, southern, eastern and western regions with very different climate and cultures), socioeconomic diversity (including affluent cities and impoverished inland rural areas, as well as cities of different population sizes) and taking into account past maternal and child health situation (i.e. the maternal mortality rates), immigration population proportion, population stability, local commitment and capacity in order to provide a balanced and representative sampling.

For peer review only - http://bmjopen.bmj.com/site/about/guidelines.xhtml

In the end, four megacities (Beijing in North China, Nanning of Guangxi Province in South Central China, Hefei of Anhui province, and Nanjing of Jiangsu province in East China), four medium-sized cities (Longyan of Fujian province in East China, Liaoyuan of Jilin province in Northeast China, Honghe of Yunnan province, and Luzhou of Sichuan province in Southwest China), two countryside areas (Zhengding County of Hebei province in North China and Jia County of Henan province in Central China), and two underdeveloped rural areas (Tailai county of Heilongjiang province in Northeast China and Ledu county of Qinghai province in Northwest China) were selected. In the second stage, one district or county was chosen from the 12 regions by simple random sampling method. In the third stage, a simple sampling method was implemented to select four to eight communities or villages from the chosen district or county in the 12 regions. A survey team of five full-time staff with medical qualifications and field experience was established in each of the 12 regions. Mothers with children under 12 months were identified through birth registration by the China National Center for Disease Control. Mothers whose children were under 12 months were eligible to participate in this study. To encourage involvement, incentives such as gift cards and simple household commodities were prepared for each participant. In the end, 10,408 mothers from 77 counties were recruited for the present study. After registration and obtaining informed consent, trained health staff with smartphone-based questionnaires collected participants' information. The questionnaire asked for mothers' sociodemographic information, socioeconomic information, breastfeeding behavior, and breastfeeding related environment. Over 90% of the participants completed the entire questionnaire. The Institutional Review Board at Peking University Health Science Center and the China National Center for Disease Control approved this study. All participants included in this study provided informed consent.

Variable definitions

Maternal education was the primary variable in the study. In the baseline survey, maternal education was classified into eight categories, including no formal education, did not graduate from primary school, primary

For peer review only - http://bmjopen.bmj.com/site/about/guidelines.xhtml

school, middle school, high/vocational school, associate/junior college, university, and postgraduate and above. In the present study, those with no formal school education, dropped out from primary school, or those with only a primary school education were classified into the ‘Primary School and Below’ group; those with middle school education were classified as the ‘Middle School’ group; those with high/vocational school education were classified as the ‘High/Vocational School’ group; and those with an education of associate/junior college, university, postgraduate and above were classified as the ‘College and Above’ group. This categorization enabled a balanced population in each group and made the results interpretable because primary school and below could be regarded as practically illiterate, and those that completed college and above could be seen as having higher education in China.

Four breastfeeding outcome variables including early initiation of breastfeeding (EIB), exclusive breastfeeding under six months (EBF), predominant breastfeeding under six months (PBF), and children ever breastfed (ever BF), were analyzed. EIB and ever BF were defined according to World Health Organization indicators.[19] The final calculation of EIB prevalence and ever BF prevalence was conducted among mothers with children from 0-12 months of age. EIB prevalence was defined as the proportion of children born in the last 12 months who were put to the breast within an hour after birth. Ever BF prevalence was defined as the proportion of children who were ever breastfed among all children aged 0-12 months. EBF and PBF were defined and evaluated according to Wellstart International’s toolkit for monitoring and evaluating breastfeeding activities,[20] using a 24-hour recall methodology. Mothers were asked to recall the food they fed their children in the last 24 hours, and the final calculation of EBF prevalence and PBF prevalence was conducted among mothers with children from 0-6 months. EBF prevalence was defined as the proportion of infants 0–6 months of age who were exclusively fed breast milk. PBF prevalence was defined as the proportion of infants 0–6 months of age who were predominantly breastfed, which mainly comprised children who were fed by breast milk and water.[20] Furthermore, to explore mothers’ current breastfeeding situation, the present

For peer review only - http://bmjopen.bmj.com/site/about/guidelines.xhtml

study also implemented an indicator of current breastfeeding (CBF) prevalence, defined as any breastfeeding in the last 24 hours of children less than 12 months of age.[20]

- $EIB = \frac{\text{children aged from } 0-12 \text{ months who were put to the breast within an hour after birth}}{\text{children aged from } 0-12 \text{ months}}$
- $EBF = \frac{\text{children aged from } 0-6 \text{ months who were exclusively fed breast milk in the last 24 hours}}{\text{children aged from } 0-6 \text{ months}}$
- $PBF = \frac{\text{children aged from } 0-6 \text{ months who were fed predominantly breast milk in the last 24 hours}}{\text{children aged from } 0-6 \text{ months}}$
- $Ever\ BF = \frac{\text{children aged from } 0-12 \text{ months who were ever breastfed.}}{\text{children aged from } 0-12 \text{ months}}$
- $CBF = \frac{\text{children aged from } 0-12 \text{ months who were breastfed in the last 24 hours.}}{\text{children aged from } 0-12 \text{ months}}$

Mother-infant indicators and family socioeconomic status were the two categories of covariates incorporated in the present study. Mother-infant indicators included maternal age, infant age, pre-pregnancy BMI, gestational age, infant birth weight, infant sex, parity, and delivery method. All covariate data were collected from the mother's self-reported answers, aside from infant age and pre-pregnancy BMI. Infant age was calculated by the infants' birth date and survey date. Pre-pregnancy BMI was calculated based on the mothers' self-reported weight and height before pregnancy. Apart from maternal education, family socioeconomic status included household income, residency status, and maternal occupation, which were also collected based on participants' self-reported answers. In the baseline survey, household income was a continuous variable. In the present study, household income was classified into ≤ 40,000 Yuan (1 USD ≈ 6.33 Chinese Yuan); 40,001-80,000 Yuan; and > 80,000 Yuan. Occupation was categorized as agriculture and related workers, factory workers, white-collar, or others. Residency status was classified as local or migrant, according to whether the mother lives at their place of residence. Health-seeking behaviors, including considering breastfeeding before pregnancy, attending postpartum mother support groups, and receiving breastfeeding education during the perinatal period, were analyzed in the present study.

Data analysis

Descriptive analyses were used to illustrate the basic demographic, socioeconomic, and lifestyle

For peer review only - http://bmjopen.bmj.com/site/about/guidelines.xhtml

characteristics in different maternal education groups. The data from the mothers that did not complete the questionnaires as required were excluded from the multivariable analyses. Since the prevalence of the outcomes was high, the odds ratios tended to overestimate the risk ratios. Thus, a log-binomial (or binomial log-linear regression) model[21 22] was employed to calculate the risk ratios to explore the association between maternal education and breastfeeding outcomes. All of the risk ratios were presented with a 95% confidence interval. The primary school and uneducated group were chosen to be the reference groups for all models. Two models were fitted: 1) unadjusted; 2) adjusted for maternal age, infant birth weight, infant sex, parity, delivery method, household income, region (super city/urban/rural/poor rural), residency status (local/migrant), paternal education and maternal occupation. The adjusted variables were chosen by reviewing relevant literature and was in accordance with the social determinants of breastfeeding framework.[6]

To further understand the relationship between maternal education and early initiation of breastfeeding and exclusive breastfeeding, the adjusted prevalence of the three variables was calculated after stratification by household income. The adjusted prevalence of health-seeking behaviors, including considering breastfeeding before pregnancy, attending a postpartum mother support group, and receiving breastfeeding education were also calculated in the different education groups. The adjusted prevalence was based on logistic regression, and the specific method is described elsewhere.[23] All the analyses were conducted using SAS version 9.4 (SAS Institute, Cary, North Carolina, USA).

Patient involvement

Patients were not involved in setting the research questions or planning the study. Investigators, including both the interviewers and the researchers, do not know the identities of the study participants.

RESULTS

For peer review only - http://bmjopen.bmj.com/site/about/guidelines.xhtml

Table 1 shows the baseline characteristics of the study population by maternal education. Of all 10,408 participants that were included, 7.50% finished their education at primary school or below, 36.91% had a middle school education, 19.12% had a high/vocational school education, and 36.46% had a college education and above. The mean maternal ages for those whose highest education was primary school and below, middle school, high/vocational school, and college and above were 28.76 (SD = 6.68), 28.41 (SD = 5.21), 28.26 (SD = 4.78), and 30.36 (SD = 4.51), respectively. The mean infant age (in days) for mothers whose highest education was primary school and below, middle school, high/vocational school, and college and above were 186.50 (SD=106.82), 182.11 (SD=104.48), 178.29 (SD=105.82), and 173.10 (SD=105.52), respectively. The detailed distribution of infant age at interview by maternal education can be found in *Supplement 1*. The distributions of pre-pregnancy BMI, gestational age, infant birth weight, and infant sex were similar across all maternal education groups. Women with an education level of primary school or below tended to be multiparous, had vaginal deliveries, lived in urban and poor rural areas, and had lower household incomes. Women who had primary school education or below were more likely to hold agricultural jobs (57.89%) and were less likely to be white-collar (11.77%). Women whose educational level was college and above were more likely to be migrants (46.35%) compared to women of other educational levels.

The overall EIB, CBF, ever BF, EBF, and PBF rates were 71.80%, 86.98%, 97.47%, 15.40%, and 36.37%, respectively. The prevalence of EIB was 64.85%, 68.24%, 71.11%, 77.21% and the prevalence of EBF was 26.53%, 15.36%, 13.64%, and 14.06% for mothers whose highest education was primary school and below, middle school, high/vocational school, and college and above, respectively. Detailed breastfeeding rates in mothers with different educational levels can be found at the end of *Table 1*.

Table 2 presents the relationship between breastfeeding practices and maternal education. No significant association was found between CBF and maternal education. Compared to the primary school and below

For peer review only - http://bmjopen.bmj.com/site/about/guidelines.xhtml

group, other groups showed a greater risk ratio (RR) of early initiation and ever breastfeeding. Compared to the primary school or below group, other groups had a smaller risk ratio of exclusive and predominant breastfeeding. After adjusting for possible confounders, the significant association between maternal education and breastfeeding outcomes was only found in the early initiation of breastfeeding (RR = 1.05, 95% CI: 1.01-1.11 for mothers with a middle school education; RR = 1.10, 95% CI: 1.03-1.17 for mothers with a high school/vocational school education; and RR = 1.16, 95% CI: 1.10-1.23 for mothers with a college or above education), and exclusive breastfeeding (RR = 0.66, 95% CI: 0.53-0.81 for mothers with a middle school education; RR = 0.63, 95% CI: 0.49-0.81 for mothers with a high school/vocational school education; and RR = 0.74, 95% CI: 0.65-0.83 for mothers with a college or above education). The detailed RRs and 95% CIs are found in Table 2.

Figure 1 presents the adjusted prevalence of early initiation of breastfeeding in the different maternal education groups, stratified by household income. The association between maternal education and early initiation of breastfeeding was only significant in those with household income > 80,000 Yuan. In this group, a lower prevalence of early initiation of breastfeeding (44.95%; 95% CI: 40.65 - 49.25%) was observed in those with a primary school education or below compared to those with a college education or above (78.39%; 95% CI: 76.25 - 80.52%).

Figure 2 presents the adjusted prevalence of exclusive breastfeeding in different maternal education groups, stratified by household income. There was no significant association between maternal education and exclusive breastfeeding prevalence in those with household income of 40,001- 80,000 Yuan. For those with household income of ≤ 40,000 Yuan and > 80,000 Yuan, there was a general negative association between maternal education and the incidence of exclusive breastfeeding. For those with household income of ≤ 40,000 Yuan, a higher prevalence of exclusive breastfeeding was observed in those with a primary school education

For peer review only - http://bmjopen.bmj.com/site/about/guidelines.xhtml

or below (36.54%; 95% CI: 29.93 - 43.15%) to compared to those with a college education or above (11.61%; 95% CI: 10.58 - 12.65%). For those with a household income of > 80,000 Yuan, a higher prevalence of exclusive breastfeeding was observed in those with a primary or below education (26.83%; 95% CI: 26.63 - 27.03%) compared to those with a college education or above (9.69%; 95% CI: 8.14 - 11.23%).

Figure 3 presents the adjusted prevalence of health-seeking behaviors, including considering breastfeeding before pregnancy, attending a mother support group postpartum, and receiving breastfeeding education. There was a general positive association between health-seeking behaviors and maternal education across these three factors. A lower prevalence of considering breastfeeding before pregnancy was observed in those with a primary school education or below (28.35%; 95% CI: 25.98 - 30.73%) compared to those with a college education or above (53.92%; 95% CI: 52.09 - 55.75%). A higher prevalence of attending mother support groups postpartum was observed in those with a primary school education or below (10.09%; 95% CI: 9.20 - 10.98%) compared to those with a college education or above (56.03%; 95% CI: 54.55 - 57.51%). A lower prevalence of receiving breastfeeding education was also observed in those with a primary school education or below (32.65%; 95% CI: 30.71 - 34.59%) compared to those with a college education or above (66.47%; 95% CI: 65.16 - 67.78%).

DISCUSSION

Several significant findings are illustrated in the present study. First, maternal education is inversely related to exclusive breastfeeding. Second, maternal education is positively related to the early initiation of breastfeeding in the high-income households. Third, after stratification by income, the patterns of association between maternal education and breastfeeding indicators differed in the different income groups. The positive relationship between maternal education and early initiation of breastfeeding is observed in the group with the highest household income. An inverse relationship between maternal education and exclusive breastfeeding

For peer review only - http://bmjopen.bmj.com/site/about/guidelines.xhtml

exists in the low and high household income groups, and the association is stronger in the low-income households.

This study is based on a large, national representative, population-based survey. The large sample size ensures high precision in the findings. The associations observed in the present study accorded with and contributed to the development of the social determinants of breastfeeding framework.[6] This is one of the few studies mainly focused on the association between maternal education and breastfeeding practices in China. We specified breastfeeding indicators, explored the associations separately, and stratified using possible influential factors. Quantitative data were used to analyze the health-seeking behaviors. Admittedly, there are some potential limitations in the present study. First, causal inference between maternal education and breastfeeding practices is limited due to the study being cross-sectional. Further prospective study is needed in order to establish a causal relationship. Second, maternal education in this study represents formal education gained through schooling, and it may not reflect the actual health literacy of the mothers. Third, feeding practices were determined using the 'last-24-hour method', which tends to overestimate the prevalence of EBF as compared to the 'since birth method'[24], and the information used in the analysis was all based on mothers' self-report, which might introduce recall biases. Last, one of the potential limitations of the present study is that we did not survey the number of current births among the mothers. As the association between maternal education and infant breastfeeding practices might differ in twins and singletons, this could affect the accuracy of the present findings.

In contrast to the early initiation of breastfeeding, maternal education is inversely related to exclusive breastfeeding, which is consistent with previous studies conducted in China.[18] A systematic review conducted in China exploring the relationship between maternal education and breastfeeding indicators found a negative association. The review speculated that maternal leave, occupation, and incorrect traditional perceptions

For peer review only - http://bmjopen.bmj.com/site/about/guidelines.xhtml

contributed to the negative association.[18] One of the traditional perceptions was that breastfeeding would change the mothers' body shape and have a negative impact on her health.[25] Nevertheless, our finding was opposite to those in developed countries.[14 15] In China, highly educated mothers are usually working full time in responsible and formal positions. The maternity leave regulated by legislation is 98 days in China,[26] which is not enough to guarantee the six-month exclusive breastfeeding time recommended by WHO. It is also equally difficult for mothers who have already returned to work to continue breastfeeding due to the lack of related supporting facilities and increased working pressure.[27] Another possible explanation is that highly educated mothers tend to have higher household incomes, and thus are able to afford infant formula, which is especially relevant in China.[28] Due to prevalent advertising of infant formula and a lack of proper breastfeeding education, the use of infant formula is sometimes regarded as healthier and is also a symbol of wealth in China.[28 29] Besides, incorrect perceptions in some areas maintain that breastfeeding changes a mothers' body shape.[25] Women with higher education generally live in urban areas and are of a relatively higher social class. Women in those urban areas and higher social classes may face higher social pressures. Thus, they may pay more attention to their body shape and are more willing to choose infant formula.[30]

In the present study, a positive association between the early initiation of breastfeeding and maternal education is observed in the household incomes > 80,000 Yuan. The positive association between maternal education and early initiation is consistent with other studies conducted in developed countries.[14 15 31] Previous studies in developing countries showed inconsistent results concerning the relationship between maternal education and the early initiation of breastfeeding.[13 32-34] A study conducted in Laos found that the early initiation of breastfeeding was most prevalent among mothers with higher education[32] and a study in Nepal observed the similar result. [13] A study conducted in Vietnam, however, found that mothers with no education were more likely to initiate breastfeeding.[34] Results from the WHO Global Survey that was conducted primarily in developing countries found an overall negative association between maternal education and early initiation of

For peer review only - http://bmjopen.bmj.com/site/about/guidelines.xhtml

breastfeeding.[33] The positive association in this study needs to be explored. In the present study, mothers with higher educational attainment tended to exhibit proactive health behaviors, early consideration of feeding methods, and were more likely to receive breastfeeding education, all of which may foster the early initiation of breastfeeding. It has been suggested that women with higher education tend to have stronger social support,[35] and are more likely to attend mother support groups, which was also observed in this study. Previous studies have found that social support is an important contributor to the early initiation of breastfeeding.[36 37] Furthermore, women with higher education are more likely to live in super cities and have a higher household income. There is a considerable disparity of health services between rural and urban areas in China.[38] Thus, women who live in super cities are more likely to have better access to quality health services. Previous studies found that the professional knowledge from nurses and doctors may foster early initiation of breastfeeding.[39 40] A review conducted in India suggested that interventions designed to increase the knowledge and skills of healthcare workers were important in promoting the early initiation of breastfeeding.[41]

After stratification by income, the patterns of association between maternal education and breastfeeding indicators differed across income groups. The positive relationship between maternal education and the early initiation of breastfeeding was only significant in the high household income group. Higher education may promote better breastfeeding knowledge and health behaviors.[42] Nevertheless, apart from knowledge, the early initiation of breastfeeding requires social support and access to healthcare services.[3] Women with lower household incomes may not have the financial ability to obtain healthcare services. These women are more likely to give birth at home, where initiation of breastfeeding within one hour of birth may not be promoted. Mothers may recognize the benefits of delivery at healthcare facilities, yet they may not be able to afford the expensive hospitals stays and professional services. The inverse relationship between maternal education and exclusive breastfeeding was the strongest in low-income households. A possible explanation is that women with a lower income and higher education tend to have a heavier workload in order to meet the demands of

For peer review only - http://bmjopen.bmj.com/site/about/guidelines.xhtml

the family, which consequently shortened the duration of exclusive breastfeeding. People with a lower income tend to live in more rural areas, where health infrastructure and the expertise of healthcare workers may be less advanced than urban areas. Traditional values may also be more prevalent than in the cities, such as routine supplementary feeding with water,[43] which may have given rise to the associations found in this study.

Associations found in the present study had implications for future public health interventions to promote breastfeeding in China. There are also several related factors to consider when designing such interventions. In the present study, ever BF rates were much higher than EIB rates, which might be partly due to the high cesarean section prevalence in China.[7] Baby Friendly Hospital Initiative is utilized in most of the hospitals with obstetrical departments in China and has had positive impacts on breastfeeding practices, but the implementation of this Initiative was highly questionable due to a lack of routine monitoring and evaluation, which might reduce EIB rates.[7 44] Sociocultural norms in China, such as postpartum confinement, are widely practiced, which may also affect mothers' breastfeeding behaviors.[43] In addition, despite a nation-wide melamine milk scandal in 2008, the infant formula marketing became even more prevalent in China [28 29] after the Chinese government abolished the International Code of Marketing of Breast-milk Substitutes.[45] All of these suggest that an extensive public campaign involving multisectoral stakeholders to promote breastfeeding practices is highly needed in China.

Conclusions

A positive association between maternal education and the early initiation of breastfeeding, and a negative association between maternal education and exclusive breastfeeding were observed in this study, with the consideration of health-seeking behavior and household income. Routine and successful breastfeeding, as per the American Academy of Pediatrics recommendation,[1] is crucial for the health of the infant and is primarily influenced by social factors. This study underscores the influence of maternal education on breastfeeding

For peer review only - http://bmjopen.bmj.com/site/about/guidelines.xhtml

practices. Findings from this study contribute to the growing body of research on the social determinants of breastfeeding. This study suggests that extensive public health interventions on breastfeeding promotion in China should target mothers with higher levels of education, especially among those with medium to high incomes. Appropriate supportive policies and programs for this group of mothers, including enforcing full-term maternal leave, providing breastfeeding education and counseling, building a mother-friendly workplace, as well as breaking cultural and social norms, should be implemented to create an encouraging environment for breastfeeding practice among Chinese mothers.

Contributors

KT and HW contributed to the study concept and design, statistical analysis, results interpretation, and drafting and revision of the manuscript. ST contributed to the study concept and design, drafting and revision of the manuscript. TX contributed to the study concept and design, revision of the manuscript. YW and TT contributed to revision of the manuscript. YL and XQ contributed to the study concept and design and results interpretation. JG contributed to the study concept and design. All authors read and approved the final version of the manuscript.

Funding

This study was supported by Bill & Melinda Gates Foundation and China Development Research Foundation.

Competing interests

None declared.

Patient consent

Obtained.

Ethics approval

This study was conducted according to the guidelines laid down in the Declaration of Helsinki and all procedures involving human subjects/patients has been approved by the Institutional Review Boards at Peking

For peer review only - http://bmjopen.bmj.com/site/about/guidelines.xhtml

University Health Science Center and the China National Center for Disease Control. The participants included in this study provided written informed consent.

Data sharing statement

Data are available upon reasonable request

Acknowledgement

We are grateful for Chinese Center For Disease Control And Prevention for providing the data.

References

1. Eidelman AI, Schanler RJ, Johnston M, et al. Breastfeeding and the use of human milk. *Pediatrics* 2012;129(3):e827-e41.
2. Cai X, Wardlaw T, Brown DW. Global trends in exclusive breastfeeding. *International breastfeeding journal* 2012;7(1):12.
3. Victora CG, Bahl R, Barros AJ, et al. Breastfeeding in the 21st century: epidemiology, mechanisms, and lifelong effect. *The Lancet* 2016;387(10017):475-90.
4. Yang Z, Lai J, Yu D, et al. Breastfeeding rates in China: a cross-sectional survey and estimate of benefits of improvement. *The Lancet* 2016;388:S47.
5. World Health Organization. Acceptable medical reasons for use of breast-milk substitutes. 2009
6. Rollins NC, Bhandari N, Hajeebhoy N, et al. Why invest, and what it will take to improve breastfeeding practices? *The Lancet* 2016;387(10017):491-504.
7. Xu F, Qiu L, Binns CW, et al. Breastfeeding in China: a review. *International Breastfeeding Journal* 2009;4(1):6.
8. Jiang H, Li M, Yang D, et al. Awareness, intention, and needs regarding breastfeeding: findings from first-time mothers in Shanghai, China. *Breastfeeding Medicine* 2012;7(6):526-34.
9. Meyrose A-K, Klasen F, Otto C, et al. Benefits of maternal education for mental health trajectories across childhood and adolescence. *Social Science & Medicine* 2018;202:170-78.
10. Güneş PM. The role of maternal education in child health: Evidence from a compulsory schooling law. *Economics of Education Review* 2015;47:1-16.
11. Desai S, Alva S. Maternal education and child health: Is there a strong causal relationship? *Demography* 1998;35(1):71-81.
12. Skafida V. The relative importance of social class and maternal education for breast-feeding initiation. *Public health nutrition* 2009;12(12):2285-92.
13. Acharya P, Khanal V. The effect of mother's educational status on early initiation of breastfeeding: further analysis of three consecutive Nepal Demographic and Health Surveys. *BMC public health* 2015;15(1):1069.
14. Heck KE, Braveman P, Cubbin C, et al. Socioeconomic status and breastfeeding initiation among California mothers. *Public health reports* 2006;121(1):51-59.
15. Bertini G, Perugi S, Dani C, et al. Maternal education and the incidence and duration of breast feeding: a prospective study. *Journal of pediatric gastroenterology and nutrition* 2003;37(4):447-52.
16. Alemayehu T, Haidar J, Habte D. Determinants of exclusive breastfeeding practices in Ethiopia. *Ethiopian Journal of Health Development* 2009;23(1)
17. Mihrshahi S, Kabir I, Roy SK, et al. Determinants of infant and young child feeding practices in Bangladesh: secondary data analysis of Demographic and Health Survey 2004. *Food and nutrition*

For peer review only - http://bmjopen.bmj.com/site/about/guidelines.xhtml

bulletin 2010;31(2):295-313.

18. Zhao J, Zhao Y, Du M, et al. Maternal education and breastfeeding practices in China: A systematic review and meta-analysis. *Midwifery* 2017;50:62-71.
19. World Health Organization. Indicators for assessing infant and young child feeding practices: conclusions of a consensus meeting held 6-8 November 2007 in Washington DC, USA: World Health Organization (WHO) 2008.
20. Lung'aho M, Huffman SL, Labbok MH, et al. Tool kit for monitoring and evaluating breastfeeding practices and programs. *Washington, DC: Wellstart International* 1996
21. Barros AJ, Hirakata VN. Alternatives for logistic regression in cross-sectional studies: an empirical comparison of models that directly estimate the prevalence ratio. *BMC medical research methodology* 2003;3(1):21.
22. Greenland S. Model-based estimation of relative risks and other epidemiologic measures in studies of common outcomes and in case-control studies. *American journal of epidemiology* 2004;160(4):301-05.
23. Santos CAS, Fiaccone RL, Oliveira NF, et al. Estimating adjusted prevalence ratio in clustered cross-sectional epidemiological data. *BMC Medical Research Methodology* 2008;8(1):80.
24. Khanal V, Lee AH, Scott JA, et al. Implications of methodological differences in measuring the rates of exclusive breastfeeding in Nepal: findings from literature review and cohort study. *BMC pregnancy and childbirth* 2016;16(1):389.
25. Gao Ll, Chan SWc, You L, et al. Experiences of postpartum depression among first-time mothers in mainland China. *Journal of Advanced Nursing* 2010;66(2):303-12.
26. Jia N, Dong X-y, Song Y-p. Paid Maternity Leave and Breastfeeding in Urban China. *Feminist Economics* 2017:1-23.
27. Bai DL, Fong DYT, Tarrant M. Factors associated with breastfeeding duration and exclusivity in mothers returning to paid employment postpartum. *Maternal and child health journal* 2015;19(5):990-99.
28. Kent G. Global infant formula: monitoring and regulating the impacts to protect human health. *International breastfeeding journal* 2015;10(1):6.
29. Tang L, Lee AH, Binns CW, et al. Widespread usage of infant formula in China: a major public health problem. *Birth* 2014;41(4):339-43.
30. Gottschang SZ. Infant Feeding and the Feminine Body in Urban China. *China urban: Ethnographies of contemporary culture* 2001
31. Meedya S, Fahy K, Kable A. Factors that positively influence breastfeeding duration to 6 months: a literature review. *Women and Birth* 2010;23(4):135-45.
32. Kounnavong S, Pak-Gorstein S, Akkhavong K, et al. Key determinants of optimal breastfeeding practices in Laos. *Food and Nutrition Sciences* 2013;4(10):61.
33. Takahashi K, Ganchimeg T, Ota E, et al. Prevalence of early initiation of breastfeeding and determinants of delayed initiation of breastfeeding: secondary analysis of the WHO global survey. *Scientific reports* 2017;7:44868.
34. Bui QT-T, Lee H-Y, Le AT-K, et al. Trends and determinants for early initiation of and exclusive breastfeeding under six months in Vietnam: results from the Multiple Indicator Cluster Surveys, 2000–2011. *Global health action* 2016;9(1):29433.
35. Heaney CA, Israel BA. Social networks and social support. *Health behavior and health education: Theory, research, and practice* 2008;4:189-210.
36. Patel A, Bucher S, Pusdekar Y, et al. Rates and determinants of early initiation of breastfeeding and exclusive breast feeding at 42 days postnatal in six low and middle-income countries: A prospective cohort study. *Reproductive health* 2015;12(2):S10.
37. Bai DL, Fong DYT, Lok KYW, et al. Relationship between the infant feeding preferences of Chinese

For peer review only - http://bmjopen.bmj.com/site/about/guidelines.xhtml

mothers' immediate social network and early breastfeeding cessation. *Journal of Human Lactation* 2016;32(2):301-08.

38. Liu M, Zhang Q, Lu M, et al. Rural and urban disparity in health services utilization in China. *Medical care* 2007;45(8):767-74.

39. Balogun OO, Dagvadorj A, Anigo KM, et al. Factors influencing breastfeeding exclusivity during the first 6 months of life in developing countries: a quantitative and qualitative systematic review. *Maternal & child nutrition* 2015;11(4):433-51.

40. Sharma IK, Byrne A. Early initiation of breastfeeding: a systematic literature review of factors and barriers in South Asia. *International breastfeeding journal* 2016;11(1):17.

41. Aguayo VM, Gupta G, Singh G, et al. Early initiation of breastfeeding on the rise in India. *BMJ global health* 2016;1(2):e000043.

42. Adler NE, Boyce T, Chesney MA, et al. Socioeconomic status and health: the challenge of the gradient. *American psychologist* 1994;49(1):15.

43. Raven JH, Chen Q, Tolhurst RJ, et al. Traditional beliefs and practices in the postpartum period in Fujian Province, China: a qualitative study. *BMC Pregnancy and Childbirth* 2007;7(1):8.

44. Tang L, Binns CW, Lee AH, et al. Low prevalence of breastfeeding initiation within the first hour of life in a rural area of Sichuan Province, China. *Birth* 2013;40(2):134-42.

45. China NHaFPCotPsRo. Order No. 17: National health and family planning Commission's decree on deciding to repeal the regulations on the sale of substitutes for breast milk substitutes. 2017

For peer review only

For peer review only - http://bmjopen.bmj.com/site/about/guidelines.xhtml

Table 1 Demographic and social-economic characteristics of the participants

	Maternal Education			
	Primary school and below (N=781)	Middle School (N=3842)	High School/Vocational School (N=1990)	College and above (N=3795)
Mean maternal age, year (SD)	28.76(6.68)	28.41(5.21)	28.26(4.78)	30.36(4.51)
Mean pre-pregnancy BMI, Kg/m^2 (SD)	22.67(4.66)	22.44(4.16)	21.74(4.04)	21.75(4.12)
Mean gestational age, week (SD)	39.02(1.38)	39.03(1.40)	38.94(1.51)	38.98(1.38)
Mean infant birth weight, kg (SD)	3.23(0.47)	3.33(0.59)	3.34(0.67)	3.43(0.75)
Mean infant age, day (SD)	186.50(106.82)	182.11(104.48)	178.29(105.82)	173.10(105.52)
Infant sex, %				
Male	50.67	50.64	52.17	50.08
Female	49.33	49.36	47.83	49.92
Parity, %				
Primiparous	29.70	33.87	43.49	62.06
Multiparous	70.30	66.13	56.51	37.94
Delivery method, %				
Vaginal	65.77	56.72	54.59	57.01
Cesarean section	34.23	43.28	45.41	42.99
Region, %				
Super city	9.70	14.16	30.82	61.55
Urban	43.15	32.08	39.09	27.41
Rural	7.15	25.39	21.09	7.29
Poor rural	40.00	28.37	9.00	3.75
Household income, %				
≤40,000 yuan	61.74	37.44	20.06	11.81
40,000-79,999 yuan	22.39	30.01	26.21	13.82
≥80,000 yuan	3.48	7.84	16.40	38.89
Maternal Occupation, %				
Agriculture related	57.89	33.61	9.37	2.27
Factory workers	11.53	19.95	20.31	8.66
White-collar	11.77	26.74	47.23	73.03
Others[1]	18.81	19.71	23.09	16.04
Resident status, %				
Local	69.70	69.15	59.50	53.65
Migrant	30.30	30.85	40.50	46.35
Breastfeeding practice, %				
Early initiation	64.85	68.24	71.11	77.21
Current BF	87.88	88.53	85.09	86.21

[1] Others refer to unidentified occupation, unemployed, and housewives

For peer review only - http://bmjopen.bmj.com/site/about/guidelines.xhtml

For peer review only

Ever BF	95.52	97.31	97.65	97.93
Exclusive BF (0-6 months)	26.53	15.36	13.64	14.06
Predominant BF (0-6 months)	50.00	39.99	18.44	39.29
Health Seeking Behaviors				
Considered feeding method before pregnancy	28.31	45.35	50.24	55.29
Attended mother support group	8.24	24.40	38.20	49.36
Received breastfeeding education	38.59	47.23	59.13	64.23

	Unadjusted Risk Ratio (95% CI)			
	Primary school and below	Middle School	High School/Vocational School	College and above
Early initiation	1	1.05(0.97,1.13)	1.10(1.01,1.19)	1.19(1.09,1.29)
Current BF	1	1.01(1.01,1.01)	0.97(0.96,1.00)	0.98(0.97,1.01)
Ever BF	1	1.02(1.02,1.03)	1.02(1.02,1.02)	1.03(1.03,1.03)
Exclusive BF (0-6 months)	1	0.58(0.52,0.64)	0.51(0.42,0.60)	0.53(0.47,0.59)
Predominant BF (0-6 months)	1	0.80(0.72,0.87)	0.37(0.24,0.50)	0.79(0.70,0.89)
	Adjusted Risk Ratio (95% CI)			
	Primary school and below	Middle School	High School/Vocational School	College and above
Early initiation	1	1.05(1.01,1.11)	1.10(1.03,1.17)	1.16(1.10,1.23)
Current BF	1	1.01(0.99,1.04)	1.02(0.99,1.05)	1.04(1.01,1.07)
Ever BF	1	1.01(1.01,1.01)	1.01(1.01,1.01)	1.01(1.01,1.01)
Exclusive BF (0-6 months)	1	0.66(0.53,0.81)	0.63(0.49,0.81)	0.74(0.65,0.83)
Predominant BF (0-6 months)	1	0.89(0.79,1.00)	0.84(0.72,0.97)	0.73(0.62,0.85)

Table 2. Relations between breastfeeding practices and maternal education*

**Adjusted for maternal age, infant birth weight, infant sex, parity, delivery method, household income, region (super city/urban/rural/poor rural), residency (local/migrant), paternal education, and occupation.*

For peer review only - http://bmjopen.bmj.com/site/about/guidelines.xhtml

Figure 1 Adjusted Prevalence* of early initiation in different maternal education groups, stratified by household Income

**After adjusting for maternal age, infant sex, parity, delivery method, paternal education, region (super city/urban/rural/poor rural), residency (local/migrant), occupation*

Figure 2 Adjusted Prevalence* of exclusive breastfeeding in different maternal education groups, stratified by household Income

*After adjusting for maternal age, infant sex, parity, delivery method, paternal education, region (super city/urban/rural/poor rural), residency (local/migrant), occupation

Figure 3 Adjusted Prevalence* of considering feeding method before pregnancy, attending mother support group, receiving breastfeeding education in different maternal education groups*

**After adjusting for maternal age, infant sex, parity, delivery method, paternal education, household income, region (super city/urban/rural/poor rural), residency (local/migrant), occupation.*

For peer review only

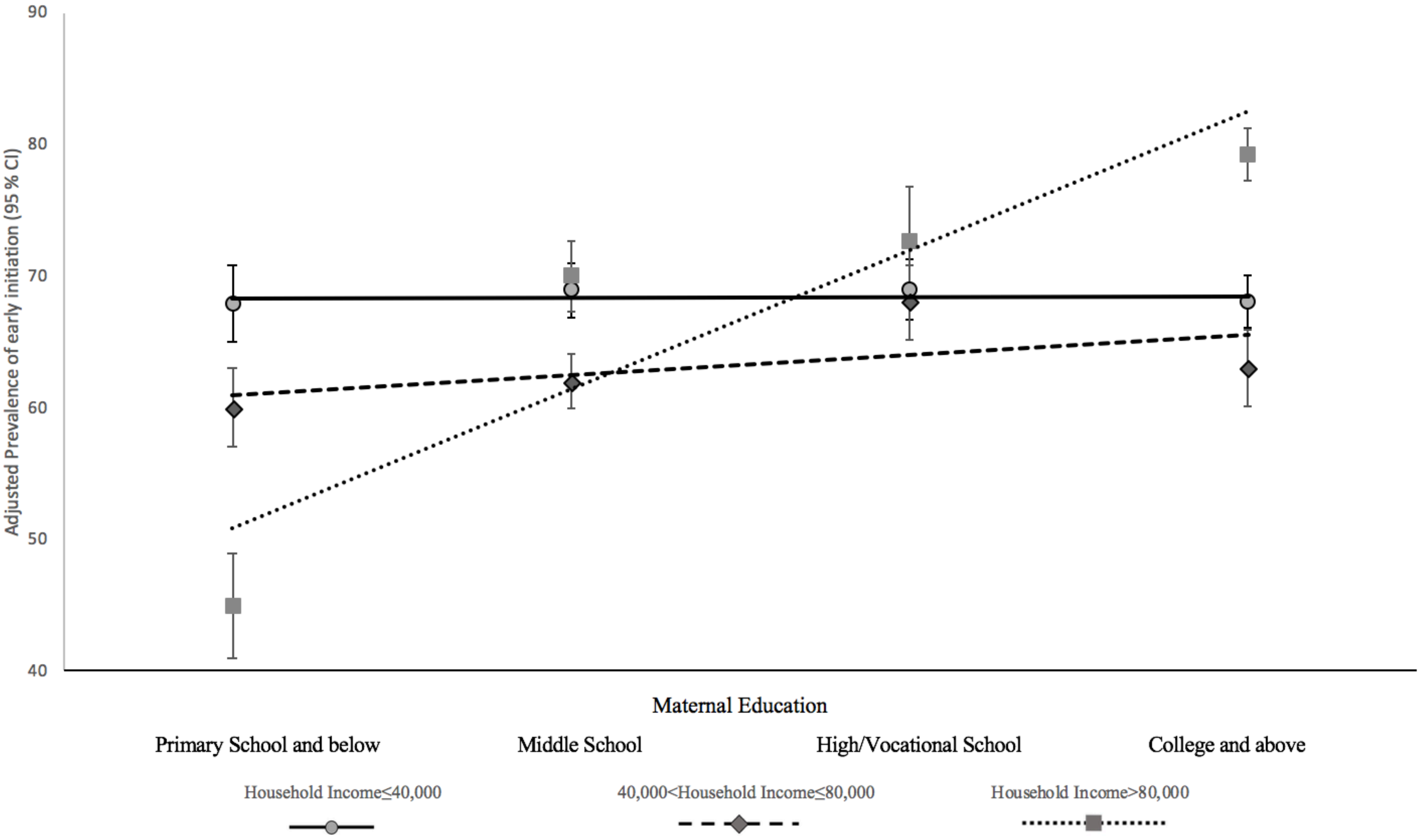
90
80
70
60
50
40
Adjusted Prevalence of early initiation (95 % CI)
Maternal Education
Primary School and below
Middle School
High/Vocational School
College and above
Household Income≤40,000
40,000<Household Income≤80,000
Household Income>80,000

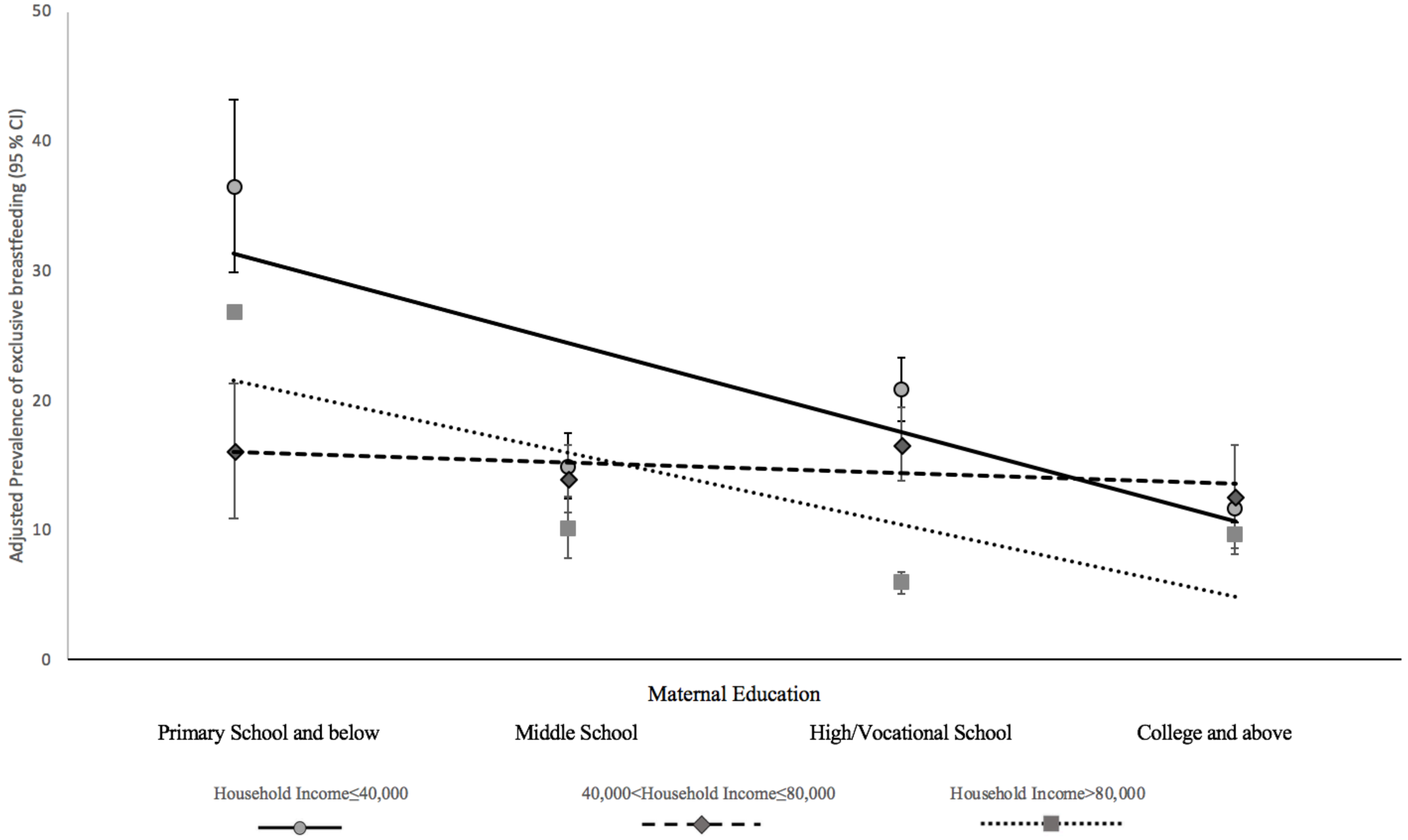
Adjusted Prevalence of exclusive breastfeeding (95 % CI)
50
40
30
20
10
0
Maternal Education
Primary School and below
Middle School
High/Vocational School
College and above
Household Income≤40,000
40,000<Household Income≤80,000
Household Income>80,000

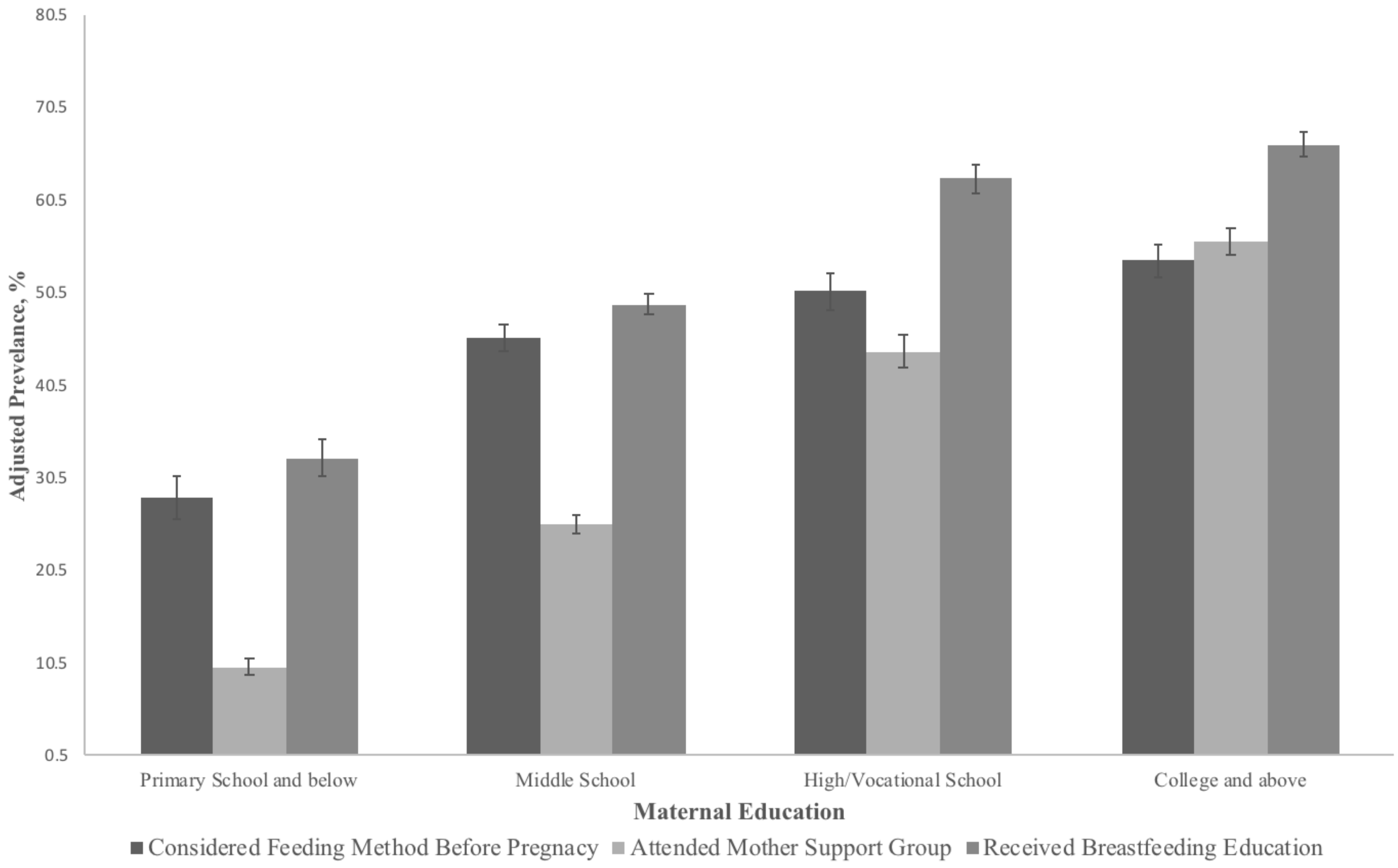
80.5
70.5
60.5
50.5
40.5
30.5
20.5
10.5
0.5
Adjusted Prevelance, %
Primary School and below
Middle School
High/Vocational School
College and above
Maternal Education
Considered Feeding Method Before Pregnacy
Attended Mother Support Group
Received Breastfeeding Education

Supplementary 1. Infant age at interview by maternal education.

	Maternal Education			
	Primary school and below (N=781)	Middle School (N=3842)	High School/Vocational School (N=1990)	College and above (N=3795)
Mean infant age, day (SD)	186.50(106.82)	182.11(104.48)	178.29(105.82)	173.10(105.52)
Range of infant age, day	0-364	0-364	0-364	0-364
Median	190.00	183.00	181.00	170.00
Distribution of infant age, %				
0~91 days (>0, ≤3 months)	23.30	24.83	25.83	27.27
92~183 days (>3, ≤6 months)	24.46	25.25	25.08	26.03
184~274 days (>6, ≤9 months)	24.33	25.43	26.13	23.90
275~364 days (>9, ≤12 months)	27.91	24.49	22.96	22.79

For peer review only

Reporting checklist for cross sectional study.

Based on the STROBE cross sectional guidelines.

Instructions to authors

Complete this checklist by entering the page numbers from your manuscript where readers will find each of the items listed below.

Your article may not currently address all the items on the checklist. Please modify your text to include the missing information. If you are certain that an item does not apply, please write "n/a" and provide a short explanation.

Upload your completed checklist as an extra file when you submit to a journal.

In your methods section, say that you used the STROBE cross sectional reporting guidelines, and cite them as:

von Elm E, Altman DG, Egger M, Pocock SJ, Gotzsche PC, Vandenbroucke JP. The Strengthening the Reporting of Observational Studies in Epidemiology (STROBE) Statement: guidelines for reporting observational studies.

For peer review only

		Reporting Item	Page Number
Title	#1a	Indicate the study's design with a commonly used term in the title or the abstract	1
Abstract	#1b	Provide in the abstract an informative and balanced summary of what was done and what was found	2-3
Background / rationale	#2	Explain the scientific background and rationale for the investigation being reported	4
Objectives	#3	State specific objectives, including any prespecified hypotheses	5
Study design	#4	Present key elements of study design early in the paper	5
Setting	#5	Describe the setting, locations, and relevant dates, including periods of recruitment, exposure, follow-up, and data collection	5-6
Eligibility criteria	#6a	Give the eligibility criteria, and the sources and methods of selection of participants.	5-6

	#7	Clearly define all outcomes, exposures, predictors, potential confounders, and effect modifiers. Give diagnostic criteria, if applicable	6-7
Data sources / measurement	#8	For each variable of interest give sources of data and details of methods of assessment (measurement). Describe comparability of assessment methods if there is more than one group. Give information separately for for exposed and unexposed groups if applicable.	6-8
Bias	#9	Describe any efforts to address potential sources of bias	6-8
Study size	#10	Explain how the study size was arrived at	5-6
Quantitative variables	#11	Explain how quantitative variables were handled in the analyses. If applicable, describe which groupings were chosen, and why	6-8
Statistical methods	#12a	Describe all statistical methods, including those used to control for confounding	8-9
	#12b	Describe any methods used to examine subgroups and interactions	8-9
	#12c	Explain how missing data were addressed	8-9
	#12d	If applicable, describe analytical methods taking account of sampling strategy	8-9
	#12e	Describe any sensitivity analyses	8-9
Participants	#13a	Report numbers of individuals at each stage of study—eg numbers potentially eligible, examined for eligibility, confirmed eligible, included in the study, completing follow-up, and analysed. Give information separately for for exposed and unexposed groups if applicable.	5-6
	#13b	Give reasons for non-participation at each stage	5-6
	#13c	Consider use of a flow diagram	5-6
Descriptive data	#14a	Give characteristics of study participants (eg demographic, clinical, social) and information on exposures and potential confounders. Give information separately for exposed and unexposed groups if applicable.	5-6
	#14b	Indicate number of participants with missing data for each variable of interest	5-6

For peer review only

Outcome data	#15	Report numbers of outcome events or summary measures. Give information separately for exposed and unexposed groups if applicable.	8
Main results	#16a	Give unadjusted estimates and, if applicable, confounder-adjusted estimates and their precision (eg, 95% confidence interval). Make clear which confounders were adjusted for and why they were included	9-11
	#16b	Report category boundaries when continuous variables were categorized	9-11
	#16c	If relevant, consider translating estimates of relative risk into absolute risk for a meaningful time period	9-11
Other analyses	#17	Report other analyses done—e.g., analyses of subgroups and interactions, and sensitivity analyses	9-10
Key results	#18	Summarise key results with reference to study objectives	12
Limitations	#19	Discuss limitations of the study, taking into account sources of potential bias or imprecision. Discuss both direction and magnitude of any potential bias.	12
Interpretation	#20	Give a cautious overall interpretation considering objectives, limitations, multiplicity of analyses, results from similar studies, and other relevant evidence.	13-15
Generalisability	#21	Discuss the generalisability (external validity) of the study results	13-15
Funding	#22	Give the source of funding and the role of the funders for the present study and, if applicable, for the original study on which the present article is based	17

The STROBE checklist is distributed under the terms of the Creative Commons Attribution License CC-BY. This checklist was completed on 10. December 2018 using http://www.goodreports.org/, a tool made by the EQUATOR Network in collaboration with Penelope.ai

For peer review only

The association between maternal education and breastfeeding practices in China: a population-based cross-sectional study

Journal:	*BMJ Open*
Manuscript ID	bmjopen-2018-028485.R3
Article Type:	Research
Date Submitted by the Author:	11-Jul-2019
Complete List of Authors:	Tang, Kun; Tsinghua University, Research Center for Public Health<br>Wang, Hanyu; Peking University Health Science Centre, Institute for Medical Humanities<br>Tan, Shi Hui; Peking University Health Science Centre, School of Basic Medical Sciences<br>Xin, Tong; Peking University Health Science Centre, Department of Global Health<br>Qu, Xueqi; Peking University Health Science Centre, School of Public Health<br>Tang, Tianyu; Tsinghua University, Research Center for Public Health<br>Wang, Yuqi; Peking University Health Science Centre, Institute for Medical Humanities<br>Liu, Yuning; Peking University Health Science Centre, School of Public Health<br>Gaoshan, Junjian; United Nations Population Fund China Office
**Primary Subject Heading**:	Public health
Secondary Subject Heading:	Nutrition and metabolism, Epidemiology
Keywords:	breastfeeding, EPIDEMIOLOGY, China, Maternal medicine < OBSTETRICS, SOCIAL MEDICINE

SCHOLARONE™
Manuscripts

The association between maternal education and breastfeeding practices in China: a population-based cross-sectional study

Authors: Kun Tang[1],* Hanyu Wang[2],* Shi Hui Tan[3], Tong Xin[4], Xueqi Qu[5], Tianyu Tang[6], Yuqi Wang[7], Yuning Liu[8], , Junjian Gaoshan[9]

*Co-first authors

[1] Research Center for Public Health, Tsinghua University, Beijing, China. E-mail: tangkun@post.harvard.edu

[2] Institute for Medical Humanities, Peking University Health Science Center, Beijing, China. E-mail: whybest@pku.edu.cn

[3] School of Basic Medical Sciences, Peking University Health Science Center, Beijing, China. E-mail: shi.tan@mail.mcgill.ca

[4] Department of Global Health, Peking University Health Science Center, Beijing, China. E-mail: xtong.katrina@gmail.com

[5] School of Public Health, Peking University Health Science Center, Beijing, China. E-mail: quxueqisnow@163.com

[6] Research Center for Public Health, Tsinghua University, Beijing, China. E-mail: tianyu.tang@outlook.com

[7] Institute for Medical Humanities, Peking University Health Science Center, Beijing, China. E-mail: 18811761299@163.com

[8] School of Public Health, Peking University Health Science Center, Beijing, China. E-mail: lynpkuhsc@126.com

[9] United Nations Population Fund China Office. E-mail: j.gaoshan@gmail.com

Corresponding Author: Dr. Kun Tang

Address: Tsinghua University, Haidian District, Beijing 100191, P.R. China

Telephone Number: 86-10-82805951

E-mail Address：tangkun@post.harvard.edu

Keywords: Breastfeeding, maternal education, income, social determinants, China

Word count: 300 for the abstract and 3,979 for the main body of text

For peer review only - http://bmjopen.bmj.com/site/about/guidelines.xhtml

Abstract:

Objective:

To investigate the association between maternal education and breastfeeding in the Chinese population, with a consideration of household income and health-seeking behaviors.

Design:

A nationally representative population-based cross-sectional study.

Setting:

77 counties from 12 geographically distinct regions in China.

Participants:

10,408 mothers with children from 0-12 months of age, aged 15-53 years old (Mean: 29.15, SD: 5.11) were classified into primary school or below group (n=781), middle school group (n=3,842), high school/vocational school group (n=1,990), college or above group (n=3,795), according to their highest completed education.

Outcomes:

Five breastfeeding outcomes, including early initiation of breastfeeding (EIB), exclusive breastfeeding under six months (EBF), predominant breastfeeding under six months (PBF), current breastfeeding (CBF), and children ever breastfed, were calculated based on the standardized questionnaire from the WHO and Wellstart International's toolkit for monitoring and evaluating breastfeeding activities.

Results:

The absolute risk of EIB and EBF in the lowest maternal education level was 64.85% and 26.53%, respectively while the absolute risk of EIB and EBF in the highest maternal education level was 77.21% and 14.06%, respectively. A higher level of maternal education was positively associated with EIB (RR: 1.22; 95% CI: 1.12-1.30) and was inversely associated with EBF (RR: 0.59; 95% CI: 0.38-0.88). Stratified by household income, a positive association with EIB was observed only in the group with the highest household income

For peer review only - http://bmjopen.bmj.com/site/about/guidelines.xhtml

and an inverse association with EBF was found in both low and high household income groups.

Conclusions:

Mothers with a higher education were more likely to initiate early breastfeeding when they were also from a high-income household while also being less likely to exclusively breastfeed their babies. Routine and successful nursing are crucial for the health of infants and is influenced by maternal education. Future public health interventions to promote breastfeeding should consider the issues related to the educational level of mothers.

Strengths and limitations of this study

- The present study is a nationally representative population-based study with a large sample size, and is based on the first survey conducted in China to specifically explore the social determinants of breastfeeding.
- This is the first study to investigate the relationship between maternal education and comprehensive breastfeeding patterns on a national scale in China.
- We specified breastfeeding indicators and explored the associations separately and stratified by possible influential factors.
- Maternal education in this study represents the formal education gained through schooling and it may not reflect the actual health literacy of the mothers.
- Information was based on self-report in the survey and feeding practices were determined using the 'last-24-hour method', which tends to overestimate the prevalence of exclusive breastfeeding as compared to the 'since birth method.'

Funding

This study was supported by Bill & Melinda Gates Foundation and China Development Research Foundation.

For peer review only - http://bmjopen.bmj.com/site/about/guidelines.xhtml

INTRODUCTION

Extensive research has reported the diverse and compelling advantages of breastfeeding for infants, mothers, families, and society.[1] Breastfeeding has become more common in the last few years because of numerous interventions conducted at multiple levels from the communities, hospitals, and society as a whole, but the breastfeeding rates remain below optimal levels recommended by the World Health Organization (WHO) in many countries.[2 3] According to a recent cross-sectional report of 55 counties from 30 provinces in China, the prevalence of mother ever breastfeed was 79.6%, and only 20.8% of 14,539 children surveyed were exclusively breastfed at 6 months.[4]

WHO recognizes almost all mothers as biologically capable to breastfeed, except in a small number of health conditions, and that the use of breast milk substitutes is justifiable.[5] Breastfeeding practices, however, are not merely biological issues but are also related to health behaviors and are influenced by multifactorial determinants, including historical, socioeconomic, and cultural factors.[6] Women's breastfeeding practices are also affected by personal attributes, such as age, weight, education, and confidence.[6] In China, breastfeeding practices face unique challenges, due to China's diverse cultural values, political system, and historical perception of breastfeeding.[7 8]

The education status of the mother has been identified as an important determinant for child welfare, survival, and health.[9-11] Skafida et al. found that maternal education was a more informative predictor of breastfeeding initiation than occupation.[12] Although maternal education and breastfeeding practices have been studied extensively and most of the previous literature reported a positive association between maternal education and breastfeeding practices, the positive association may not be accurate in all contexts.[13-17] Studies conducted in

For peer review only - http://bmjopen.bmj.com/site/about/guidelines.xhtml

Nepal, the United States, and Italy showed that higher maternal education level was related to better breastfeeding practices.[13-15] However, studies in Ethiopia and Bangladesh observed a negative association between breastfeeding and maternal education.[16 17]

Most of the previous studies conducted in China explored the effect of maternal education on breastfeeding, together with other socioeconomic factors. The results across studies were different and sometimes reached opposing conclusions.[18] Using data from 12 regions in China, the aim of this study is to investigate the association between maternal education and breastfeeding, taking into account household income and health-seeking behaviors. The findings of this study contribute to the growing body of evidence on the social determinants of breastfeeding and have the potential to provide evidence for related interventions and policies.

METHODS

Study design and participants

This population-based study was conducted in 12 geographically defined regions in China from July 2017 to January 2018. Multi-stage sampling technique was adopted for the selection of the participants. In the first stage, population proportionate sampling (PPS) method was applied, due to the population structure and the status of maternal and child health and socioeconomic development. The selection of the 12 study sites was made carefully, aiming to maximize geographic diversity (including northern, southern, eastern and western regions with very different climate and cultures), socioeconomic diversity (including affluent cities and impoverished inland rural areas, as well as cities of different population sizes) and taking into account past maternal and child health situation (i.e. the maternal mortality rates), immigration population proportion, population stability, local commitment and capacity in order to provide a balanced and representative sampling. In the end, four megacities (Beijing in North China, Nanning of Guangxi Province in South Central China, Hefei of Anhui province, and Nanjing of Jiangsu province in East China), four medium-sized cities (Longyan

For peer review only

of Fujian province in East China, Liaoyuan of Jilin province in Northeast China, Honghe of Yunnan province, and Luzhou of Sichuan province in Southwest China), two countryside areas (Zhengding County of Hebei province in North China and Jia County of Henan province in Central China), and two underdeveloped rural areas (Tailai county of Heilongjiang province in Northeast China and Ledu county of Qinghai province in Northwest China) were selected. In the second stage, one district or county was chosen from the 12 regions by simple random sampling method. In the third stage, a simple sampling method was implemented to select four to eight communities or villages from the chosen district or county in the 12 regions. A survey team of five full-time staff with medical qualifications and field experience was established in each of the 12 regions. Mothers with children under 12 months were identified through birth registration by the China National Center for Disease Control. Mothers whose children were under 12 months were eligible to participate in this study. To encourage involvement, incentives such as gift cards and simple household commodities were prepared for each participant. In the end, 10,408 mothers from 77 counties were recruited for the present study. After registration and obtaining informed consent, trained health staff with smartphone-based questionnaires collected participants' information. The questionnaire asked for mothers' sociodemographic information, socioeconomic information, breastfeeding behavior, and breastfeeding related environment. Over 90% of the participants completed the entire questionnaire. The Institutional Review Board at Peking University Health Science Center and the China National Center for Disease Control approved this study. All participants included in this study provided written informed consent.

Variable definitions

Maternal education was the primary variable in the study. In the baseline survey, maternal education was classified into eight categories, including no formal education, did not graduate from primary school, primary school, middle school, high/vocational school, associate/junior college, university, and postgraduate and above. In the present study, those with no formal school education, dropped out from primary school, or those

For peer review only - http://bmjopen.bmj.com/site/about/guidelines.xhtml

with only a primary school education were classified into the 'Primary School and Below' group; those with middle school education were classified as the 'Middle School' group; those with high/vocational school education were classified as the 'High/Vocational School' group; and those with an education of associate/junior college, university, postgraduate and above were classified as the 'College and Above' group. This categorization enabled a balanced population in each group and made the results interpretable because primary school and below could be regarded as practically illiterate, and those that completed college and above could be seen as having higher education in China.

Four breastfeeding outcome variables including early initiation of breastfeeding (EIB), exclusive breastfeeding under six months (EBF), predominant breastfeeding under six months (PBF), and children ever breastfed (ever BF), were analyzed. EIB and ever BF were defined according to World Health Organization indicators.[19] The final calculation of EIB prevalence and ever BF prevalence was conducted among mothers with children from 0-12 months of age. EIB prevalence was defined as the proportion of children born in the last 12 months who were put to the breast within an hour after birth. Ever BF prevalence was defined as the proportion of children who were ever breastfed among all children aged 0-12 months. EBF and PBF were defined and evaluated according to Wellstart International's toolkit for monitoring and evaluating breastfeeding activities,[20] using a 24-hour recall methodology. Mothers were asked to recall the food they fed their children in the last 24 hours, and the final calculation of EBF prevalence and PBF prevalence was conducted among mothers with children from 0-6 months. EBF prevalence was defined as the proportion of infants 0–6 months of age who were exclusively fed breast milk. PBF prevalence was defined as the proportion of infants 0–6 months of age who were predominantly breastfed, which mainly comprised children who were fed by breast milk and water.[20] Furthermore, to explore mothers' current breastfeeding situation, the present study also implemented an indicator of current breastfeeding (CBF) prevalence, defined as any breastfeeding in the last 24 hours of children less than 12 months of age.[20]

For peer review only - http://bmjopen.bmj.com/site/about/guidelines.xhtml

- $EIB = \frac{\text{children aged from } 0-12 \text{ months who were put to the breast within an hour after birth}}{\text{children aged from } 0-12 \text{ months}}$
- $EBF = \frac{\text{children aged from } 0-6 \text{ months who were exclusively fed breast milk in the last 24 hours}}{\text{children aged from } 0-6 \text{ months}}$
- $PBF = \frac{\text{children aged from } 0-6 \text{ months who were fed predominantly breast milk in the last 24 hours}}{\text{children aged from } 0-6 \text{ months}}$
- $Ever\ BF = \frac{\text{children aged from } 0-12 \text{ months who were ever breastfed.}}{\text{children aged from } 0-12 \text{ months}}$
- $CBF = \frac{\text{children aged from } 0-12 \text{ months who were breastfed in the last 24 hours.}}{\text{children aged from } 0-12 \text{ months}}$

Mother-infant indicators and family socioeconomic status were the two categories of covariates incorporated in the present study. Mother-infant indicators included maternal age, infant age, pre-pregnancy BMI, gestational age, infant birth weight, infant sex, parity, and delivery method. All covariate data were collected from the mother's self-reported answers, aside from infant age and pre-pregnancy BMI. Infant age was calculated by the infants' birth date and survey date. Pre-pregnancy BMI was calculated based on the mothers' self-reported weight and height before pregnancy. Apart from maternal education, family socioeconomic status included household income, residency status, and maternal occupation, which were also collected based on participants' self-reported answers. In the baseline survey, household income was a continuous variable. In the present study, household income was classified into ≤ 40,000 Yuan (1 USD ≈ 6.33 Chinese Yuan); 40,001-80,000 Yuan; and > 80,000 Yuan. Occupation was categorized as agriculture and related workers, factory workers, white-collar, or others. Residency status was classified as local or migrant, according to whether the mother lives at their place of residence. Health-seeking behaviors, including considering breastfeeding before pregnancy, attending postpartum mother support groups, and receiving breastfeeding education during the perinatal period, were analyzed in the present study.

Data analysis

Descriptive analyses were used to illustrate the basic demographic, socioeconomic, and lifestyle characteristics in different maternal education groups. The data from the mothers that did not complete the questionnaires as required were excluded from the multivariable analyses. Since the prevalence of the

For peer review only - http://bmjopen.bmj.com/site/about/guidelines.xhtml

outcomes was high, the odds ratios tended to overestimate the risk ratios. Thus, a log-binomial (or binomial log-linear regression) model[21 22] was employed to calculate the risk ratios to explore the association between maternal education and breastfeeding outcomes. All of the risk ratios were presented with a 95% confidence interval. The primary school and uneducated group were chosen to be the reference groups for all models. Two models were fitted: 1) unadjusted; 2) adjusted for maternal age, infant birth weight, infant sex, parity, delivery method, household income, region (super city/urban/rural/poor rural), residency status (local/migrant), paternal education and maternal occupation. The adjusted variables were chosen by reviewing relevant literature and was in accordance with the social determinants of breastfeeding framework.[6]

To further understand the relationship between maternal education and early initiation of breastfeeding and exclusive breastfeeding, the adjusted prevalence of the three variables was calculated after stratification by household income. The adjusted prevalence of health-seeking behaviors, including considering breastfeeding before pregnancy, attending a postpartum mother support group, and receiving breastfeeding education were also calculated in the different education groups. The adjusted prevalence was based on logistic regression, and the specific method is described elsewhere.[23] All the analyses were conducted using SAS version 9.4 (SAS Institute, Cary, North Carolina, USA).

For peer review only

Patient involvement

Patients were not involved in setting the research questions or planning the study. Investigators, including both the interviewers and the researchers, do not know the identities of the study participants.

RESULTS

Table 1 shows the baseline characteristics of the study population by maternal education. Of all 10,408 participants that were included, 7.50% finished their education at primary school or below, 36.91% had a

For peer review only - http://bmjopen.bmj.com/site/about/guidelines.xhtml

middle school education, 19.12% had a high/vocational school education, and 36.46% had a college education and above. The mean maternal ages for those whose highest education was primary school and below, middle school, high/vocational school, and college and above were 28.76 (SD = 6.68), 28.41 (SD = 5.21), 28.26 (SD = 4.78), and 30.36 (SD = 4.51), respectively. The mean infant age (in days) for mothers whose highest education was primary school and below, middle school, high/vocational school, and college and above were 186.50 (SD=106.82), 182.11 (SD=104.48), 178.29 (SD=105.82), and 173.10 (SD=105.52), respectively. The detailed distribution of infant age at interview by maternal education can be found in *Supplement 1*. The distributions of pre-pregnancy BMI, gestational age, infant birth weight, and infant sex were similar across all maternal education groups. Women with an education level of primary school or below tended to be multiparous, had vaginal deliveries, lived in urban and poor rural areas, and had lower household incomes. Women who had primary school education or below were more likely to hold agricultural jobs (57.89%) and were less likely to be white-collar (11.77%). Women whose educational level was college and above were more likely to be migrants (46.35%) compared to women of other educational levels.

The overall EIB, CBF, ever BF, EBF, and PBF rates were 71.80%, 86.98%, 97.47%, 15.40%, and 36.37%, respectively. The prevalence of EIB was 64.85%, 68.24%, 71.11%, 77.21% and the prevalence of EBF was 26.53%, 15.36%, 13.64%, and 14.06% for mothers whose highest education was primary school and below, middle school, high/vocational school, and college and above, respectively. Detailed breastfeeding rates in mothers with different educational levels can be found at the end of *Table 1*.

Table 2 presents the relationship between breastfeeding practices and maternal education. No significant association was found between CBF and maternal education. Compared to the primary school and below group, other groups showed a greater risk ratio (RR) of early initiation and ever breastfeeding. Compared to the primary school or below group, other groups had a smaller risk ratio of exclusive and predominant

For peer review only - http://bmjopen.bmj.com/site/about/guidelines.xhtml

breastfeeding. After adjusting for possible confounders, the significant association between maternal education and breastfeeding outcomes was only found in the early initiation of breastfeeding (RR = 1.05, 95% CI: 1.01-1.11 for mothers with a middle school education; RR = 1.10, 95% CI: 1.03-1.17 for mothers with a high school/vocational school education; and RR = 1.16, 95% CI: 1.10-1.23 for mothers with a college or above education), and exclusive breastfeeding (RR = 0.66, 95% CI: 0.53-0.81 for mothers with a middle school education; RR = 0.63, 95% CI: 0.49-0.81 for mothers with a high school/vocational school education; and RR = 0.74, 95% CI: 0.65-0.83 for mothers with a college or above education). The detailed RRs and 95% CIs are found in Table 2.

Figure 1 presents the adjusted prevalence of early initiation of breastfeeding in the different maternal education groups, stratified by household income. The association between maternal education and early initiation of breastfeeding was only significant in those with household income > 80,000 Yuan. In this group, a lower prevalence of early initiation of breastfeeding (44.95%; 95% CI: 40.65 - 49.25%) was observed in those with a primary school education or below compared to those with a college education or above (78.39%; 95% CI: 76.25 - 80.52%).

Figure 2 presents the adjusted prevalence of exclusive breastfeeding in different maternal education groups, stratified by household income. There was no significant association between maternal education and exclusive breastfeeding prevalence in those with household income of 40,001- 80,000 Yuan. For those with household income of ≤ 40,000 Yuan and > 80,000 Yuan, there was a general negative association between maternal education and the incidence of exclusive breastfeeding. For those with household income of ≤ 40,000 Yuan, a higher prevalence of exclusive breastfeeding was observed in those with a primary school education or below (36.54%; 95% CI: 29.93 - 43.15%) to compared to those with a college education or above (11.61%; 95% CI: 10.58 - 12.65%). For those with a household income of > 80,000 Yuan, a higher prevalence of

For peer review only - http://bmjopen.bmj.com/site/about/guidelines.xhtml

For peer review only

exclusive breastfeeding was observed in those with a primary or below education (26.83%; 95% CI: 26.63 - 27.03%) compared to those with a college education or above (9.69%; 95% CI: 8.14 - 11.23%).

Figure 3 presents the adjusted prevalence of health-seeking behaviors, including considering breastfeeding before pregnancy, attending a mother support group postpartum, and receiving breastfeeding education. There was a general positive association between health-seeking behaviors and maternal education across these three factors. A lower prevalence of considering breastfeeding before pregnancy was observed in those with a primary school education or below (28.35%; 95% CI: 25.98 - 30.73%) compared to those with a college education or above (53.92%; 95% CI: 52.09 - 55.75%). A higher prevalence of attending mother support groups postpartum was observed in those with a primary school education or below (10.09%; 95% CI: 9.20 - 10.98%) compared to those with a college education or above (56.03%; 95% CI: 54.55 - 57.51%). A lower prevalence of receiving breastfeeding education was also observed in those with a primary school education or below (32.65%; 95% CI: 30.71 - 34.59%) compared to those with a college education or above (66.47%; 95% CI: 65.16 - 67.78%).

DISCUSSION

Several significant findings are illustrated in the present study. First, maternal education is inversely related to exclusive breastfeeding. Second, maternal education is positively related to the early initiation of breastfeeding in the high-income households. Third, after stratification by income, the patterns of association between maternal education and breastfeeding indicators differed in the different income groups. The positive relationship between maternal education and early initiation of breastfeeding is observed in the group with the highest household income. An inverse relationship between maternal education and exclusive breastfeeding exists in the low and high household income groups, and the association is stronger in the low-income households.

For peer review only - http://bmjopen.bmj.com/site/about/guidelines.xhtml

This study is based on a large, national representative, population-based survey. The large sample size ensures high precision in the findings. The associations observed in the present study accorded with and contributed to the development of the social determinants of breastfeeding framework.[6] This is one of the few studies mainly focused on the association between maternal education and breastfeeding practices in China. We specified breastfeeding indicators, explored the associations separately, and stratified using possible influential factors. Quantitative data were used to analyze the health-seeking behaviors. Admittedly, there are some potential limitations in the present study. First, causal inference between maternal education and breastfeeding practices is limited due to the study being cross-sectional. Further prospective study is needed in order to establish a causal relationship. Second, maternal education in this study represents formal education gained through schooling, and it may not reflect the actual health literacy of the mothers. Third, feeding practices were determined using the 'last-24-hour method', which tends to overestimate the prevalence of EBF as compared to the 'since birth method'[24], and the information used in the analysis was all based on mothers' self-report, which might introduce recall biases. Last, one of the potential limitations of the present study is that we did not survey the number of current births among the mothers. As the association between maternal education and infant breastfeeding practices might differ in twins and singletons, this could affect the accuracy of the present findings.

In contrast to the early initiation of breastfeeding, maternal education is inversely related to exclusive breastfeeding, which is consistent with previous studies conducted in China.[18] A systematic review conducted in China exploring the relationship between maternal education and breastfeeding indicators found a negative association. The review speculated that maternal leave, occupation, and incorrect traditional perceptions contributed to the negative association.[18] One of the traditional perceptions was that breastfeeding would change the mothers' body shape and have a negative impact on her health.[25] Nevertheless, our finding was

For peer review only - http://bmjopen.bmj.com/site/about/guidelines.xhtml

opposite to those in developed countries.[14 15] In China, highly educated mothers are usually working full time in responsible and formal positions. The maternity leave regulated by legislation is 98 days in China,[26] which is not enough to guarantee the six-month exclusive breastfeeding time recommended by WHO. It is also equally difficult for mothers who have already returned to work to continue breastfeeding due to the lack of related supporting facilities and increased working pressure.[27] Another possible explanation is that highly educated mothers tend to have higher household incomes, and thus are able to afford infant formula, which is especially relevant in China.[28] Due to prevalent advertising of infant formula and a lack of proper breastfeeding education, the use of infant formula is sometimes regarded as healthier and is also a symbol of wealth in China.[28 29] Besides, incorrect perceptions in some areas maintain that breastfeeding changes a mothers' body shape.[25] Women with higher education generally live in urban areas and are of a relatively higher social class. Women in those urban areas and higher social classes may face higher social pressures. Thus, they may pay more attention to their body shape and are more willing to choose infant formula.[30]

In the present study, a positive association between the early initiation of breastfeeding and maternal education is observed in the household incomes > 80,000 Yuan. The positive association between maternal education and early initiation is consistent with studies conducted in the developed countries.[14 15 31] Previous studies in developing countries showed inconsistent results concerning the relationship between maternal education and the early initiation of breastfeeding.[13 32-34] A study conducted in Laos found that the early initiation of breastfeeding was most prevalent among mothers with higher education[32] and a study in Nepal observed the similar result. [13] A study conducted in Vietnam, however, found that mothers with no education were more likely to initiate breastfeeding.[34] Results from the WHO Global Survey that was conducted primarily in developing countries found an overall negative association between maternal education and early initiation of breastfeeding.[33] The positive association in this study needs to be explored. In the present study, mothers with higher educational attainment tended to exhibit proactive health behaviors, early consideration of feeding

For peer review only - http://bmjopen.bmj.com/site/about/guidelines.xhtml

methods, and were more likely to receive breastfeeding education, all of which may foster the early initiation of breastfeeding. It has been suggested that women with higher education tend to have stronger social support,[35] and are more likely to attend mother support groups, which was also observed in this study. Previous studies have found that social support is an important contributor to the early initiation of breastfeeding.[36 37] Furthermore, women with higher education are more likely to live in super cities and have a higher household income. There is a considerable disparity of health services between rural and urban areas in China.[38] Thus, women who live in super cities are more likely to have better access to quality health services. Previous studies found that the professional knowledge from nurses and doctors may foster early initiation of breastfeeding.[39 40] A review conducted in India suggested that interventions designed to increase the knowledge and skills of healthcare workers were important in promoting the early initiation of breastfeeding.[41]

After stratification by income, the patterns of association between maternal education and breastfeeding indicators differed across income groups. The positive relationship between maternal education and the early initiation of breastfeeding was only significant in the high household income group. Higher education may promote better breastfeeding knowledge and health behaviors.[42] Nevertheless, apart from knowledge, the early initiation of breastfeeding requires social support and access to healthcare services.[3] Women with lower household incomes may not have the financial ability to obtain healthcare services. These women are more likely to give birth at home, where initiation of breastfeeding within one hour of birth may not be promoted. Mothers may recognize the benefits of delivery at healthcare facilities, yet they may not be able to afford the expensive hospitals stays and professional services. The inverse relationship between maternal education and exclusive breastfeeding was the strongest in low-income households. A possible explanation is that women with a lower income and higher education tend to have a heavier workload in order to meet the demands of the family, which consequently shortened the duration of exclusive breastfeeding. People with a lower income tend to live in more rural areas, where health infrastructure and the expertise of healthcare workers may be

For peer review only - http://bmjopen.bmj.com/site/about/guidelines.xhtml

less advanced than urban areas. Traditional values may also be more prevalent than in the cities, such as routine supplementary feeding with water,[43] which may have given rise to the associations found in this study.

Associations found in the present study had implications for future public health interventions to promote breastfeeding in China. There are also several related factors to consider when designing such interventions. In the present study, the overall EBF rates were 15.40%. A national representative survey of 14,539 infants aged under 2 years in 55 counties from 30 provinces in China indicated that the crude EBF rates were 20.7% and weighed EBF rates were 18.6%. [44] The lower EBF rates observed in this research might indicate that EBF rates have been decreasing from 2013, which should raise the attention of policymakers and public health workers. In the present study, ever BF rates were much higher than EIB rates, which might be partly due to the high cesarean section prevalence in China.[7] Baby Friendly Hospital Initiative is utilized in most of the hospitals with obstetrical departments in China and has had positive impacts on breastfeeding practices, but the implementation of this Initiative was highly questionable due to a lack of routine monitoring and evaluation, which might reduce EIB rates.[7 45] Sociocultural norms in China, such as postpartum confinement, are widely practiced, which may also affect mothers' breastfeeding behaviors.[43] In addition, despite a nation-wide melamine milk scandal in 2008, the infant formula marketing became even more prevalent in China [28 29] after the Chinese government abolished the International Code of Marketing of Breast-milk Substitutes.[46] All of these suggest that an extensive public campaign involving multisectoral stakeholders to promote breastfeeding practices is highly needed in China.

Conclusions

A positive association between maternal education and the early initiation of breastfeeding, and a negative association between maternal education and exclusive breastfeeding were observed in this study, with the consideration of health-seeking behavior and household income. Routine and successful breastfeeding, as per

For peer review only - http://bmjopen.bmj.com/site/about/guidelines.xhtml

the American Academy of Pediatrics recommendation,[1] is crucial for the health of the infant and is primarily influenced by social factors. This study underscores the influence of maternal education on breastfeeding practices. Findings from this study contribute to the growing body of research on the social determinants of breastfeeding. This study suggests that extensive public health interventions on breastfeeding promotion in China should target mothers with higher levels of education, especially among those with medium to high incomes. Appropriate supportive policies and programs for this group of mothers, including enforcing full-term maternal leave, providing breastfeeding education and counseling, building a mother-friendly workplace, as well as breaking cultural and social norms, should be implemented to create an encouraging environment for breastfeeding practice among Chinese mothers.

Contributors

KT and HW contributed to the study concept and design, statistical analysis, results interpretation, and drafting and revision of the manuscript. ST contributed to the study concept and design, drafting and revision of the manuscript. TX contributed to the study concept and design, revision of the manuscript. YW and TT contributed to revision of the manuscript. YL and XQ contributed to the study concept and design and results interpretation. JG contributed to the study concept and design. All authors read and approved the final version of the manuscript.

Funding

This study was supported by Bill & Melinda Gates Foundation and China Development Research Foundation.

Competing interests

None declared.

Patient consent

Obtained.

Ethics approval

For peer review only - http://bmjopen.bmj.com/site/about/guidelines.xhtml

For peer review only

This study was conducted according to the guidelines laid down in the Declaration of Helsinki and all procedures involving human subjects/patients has been approved by the Institutional Review Boards at Peking University Health Science Center and the China National Center for Disease Control. The participants included in this study provided written informed consent.

Data sharing statement

Data are available upon reasonable request

Acknowledgement

We are grateful for Chinese Center For Disease Control And Prevention for providing the data.

References

1. Eidelman AI, Schanler RJ, Johnston M, et al. Breastfeeding and the use of human milk. *Pediatrics* 2012;129(3):e827-e41.
2. Cai X, Wardlaw T, Brown DW. Global trends in exclusive breastfeeding. *International breastfeeding journal* 2012;7(1):12.
3. Victora CG, Bahl R, Barros AJ, et al. Breastfeeding in the 21st century: epidemiology, mechanisms, and lifelong effect. *The Lancet* 2016;387(10017):475-90.
4. Yang Z, Lai J, Yu D, et al. Breastfeeding rates in China: a cross-sectional survey and estimate of benefits of improvement. *The Lancet* 2016;388:S47.
5. World Health Organization. Acceptable medical reasons for use of breast-milk substitutes. 2009
6. Rollins NC, Bhandari N, Hajeebhoy N, et al. Why invest, and what it will take to improve breastfeeding practices? *The Lancet* 2016;387(10017):491-504.
7. Xu F, Qiu L, Binns CW, et al. Breastfeeding in China: a review. *International breastfeeding journal* 2009;4(1):6.
8. Jiang H, Li M, Yang D, et al. Awareness, intention, and needs regarding breastfeeding: findings from first-time mothers in Shanghai, China. *Breastfeeding Medicine* 2012;7(6):526-34.
9. Meyrose A-K, Klasen F, Otto C, et al. Benefits of maternal education for mental health trajectories across childhood and adolescence. *Social Science & Medicine* 2018;202:170-78.
10. Güneş PM. The role of maternal education in child health: Evidence from a compulsory schooling law. *Economics of Education Review* 2015;47:1-16.
11. Desai S, Alva S. Maternal education and child health: Is there a strong causal relationship? *Demography* 1998;35(1):71-81.
12. Skafida V. The relative importance of social class and maternal education for breast-feeding initiation. *Public health nutrition* 2009;12(12):2285-92.
13. Acharya P, Khanal V. The effect of mother's educational status on early initiation of breastfeeding: further analysis of three consecutive Nepal Demographic and Health Surveys. *BMC public health* 2015;15(1):1069.
14. Heck KE, Braveman P, Cubbin C, et al. Socioeconomic status and breastfeeding initiation among California mothers. *Public health reports* 2006;121(1):51-59.
15. Bertini G, Perugi S, Dani C, et al. Maternal education and the incidence and duration of breast feeding: a prospective study. *Journal of pediatric gastroenterology and nutrition* 2003;37(4):447-52.

For peer review only - http://bmjopen.bmj.com/site/about/guidelines.xhtml

16. Alemayehu T, Haidar J, Habte D. Determinants of exclusive breastfeeding practices in Ethiopia. *Ethiopian Journal of Health Development* 2009;23(1)

17. Mihrshahi S, Kabir I, Roy SK, et al. Determinants of infant and young child feeding practices in Bangladesh: secondary data analysis of Demographic and Health Survey 2004. *Food and nutrition bulletin* 2010;31(2):295-313.

18. Zhao J, Zhao Y, Du M, et al. Maternal education and breastfeeding practices in China: A systematic review and meta-analysis. *Midwifery* 2017;50:62-71.

19. World Health Organization. Indicators for assessing infant and young child feeding practices: conclusions of a consensus meeting held 6-8 November 2007 in Washington DC, USA: World Health Organization (WHO) 2008.

20. Lung'aho M, Huffman SL, Labbok MH, et al. Tool kit for monitoring and evaluating breastfeeding practices and programs. *Washington, DC: Wellstart International* 1996

21. Barros AJ, Hirakata VN. Alternatives for logistic regression in cross-sectional studies: an empirical comparison of models that directly estimate the prevalence ratio. *BMC medical research methodology* 2003;3(1):21.

22. Greenland S. Model-based estimation of relative risks and other epidemiologic measures in studies of common outcomes and in case-control studies. *American journal of epidemiology* 2004;160(4):301-05.

23. Santos CAS, Fiaccone RL, Oliveira NF, et al. Estimating adjusted prevalence ratio in clustered cross-sectional epidemiological data. *BMC Medical Research Methodology* 2008;8(1):80.

24. Khanal V, Lee AH, Scott JA, et al. Implications of methodological differences in measuring the rates of exclusive breastfeeding in Nepal: findings from literature review and cohort study. *BMC pregnancy and childbirth* 2016;16(1):389.

25. Gao Ll, Chan SWc, You L, et al. Experiences of postpartum depression among first-time mothers in mainland China. *Journal of Advanced Nursing* 2010;66(2):303-12.

26. Jia N, Dong X-y, Song Y-p. Paid Maternity Leave and Breastfeeding in Urban China. *Feminist Economics* 2017:1-23.

27. Bai DL, Fong DYT, Tarrant M. Factors associated with breastfeeding duration and exclusivity in mothers returning to paid employment postpartum. *Maternal and child health journal* 2015;19(5):990-99.

28. Kent G. Global infant formula: monitoring and regulating the impacts to protect human health. *International breastfeeding journal* 2015;10(1):6.

29. Tang L, Lee AH, Binns CW, et al. Widespread usage of infant formula in China: a major public health problem. *Birth* 2014;41(4):339-43.

30. Gottschang SZ. Infant Feeding and the Feminine Body in Urban China. *China urban: Ethnographies of contemporary culture* 2001

31. Meedya S, Fahy K, Kable A. Factors that positively influence breastfeeding duration to 6 months: a literature review. *Women and Birth* 2010;23(4):135-45.

32. Kounnavong S, Pak-Gorstein S, Akkhavong K, et al. Key determinants of optimal breastfeeding practices in Laos. *Food and Nutrition Sciences* 2013;4(10):61.

33. Takahashi K, Ganchimeg T, Ota E, et al. Prevalence of early initiation of breastfeeding and determinants of delayed initiation of breastfeeding: secondary analysis of the WHO global survey. *Scientific reports* 2017;7:44868.

34. Bui QT-T, Lee H-Y, Le AT-K, et al. Trends and determinants for early initiation of and exclusive breastfeeding under six months in Vietnam: results from the Multiple Indicator Cluster Surveys, 2000–2011. *Global health action* 2016;9(1):29433.

35. Heaney CA, Israel BA. Social networks and social support. *Health behavior and health education: Theory, research, and practice* 2008;4:189-210.

For peer review only - http://bmjopen.bmj.com/site/about/guidelines.xhtml

36. Patel A, Bucher S, Pusdekar Y, et al. Rates and determinants of early initiation of breastfeeding and exclusive breast feeding at 42 days postnatal in six low and middle-income countries: A prospective cohort study. *Reproductive health* 2015;12(2):S10.
37. Bai DL, Fong DYT, Lok KYW, et al. Relationship between the infant feeding preferences of Chinese mothers' immediate social network and early breastfeeding cessation. *Journal of Human Lactation* 2016;32(2):301-08.
38. Liu M, Zhang Q, Lu M, et al. Rural and urban disparity in health services utilization in China. *Medical care* 2007;45(8):767-74.
39. Balogun OO, Dagvadorj A, Anigo KM, et al. Factors influencing breastfeeding exclusivity during the first 6 months of life in developing countries: a quantitative and qualitative systematic review. *Maternal & child nutrition* 2015;11(4):433-51.
40. Sharma IK, Byrne A. Early initiation of breastfeeding: a systematic literature review of factors and barriers in South Asia. *International breastfeeding journal* 2016;11(1):17.
41. Aguayo VM, Gupta G, Singh G, et al. Early initiation of breastfeeding on the rise in India. *BMJ global health* 2016;1(2):e000043.
42. Adler NE, Boyce T, Chesney MA, et al. Socioeconomic status and health: the challenge of the gradient. *American psychologist* 1994;49(1):15.
43. Raven JH, Chen Q, Tolhurst RJ, et al. Traditional beliefs and practices in the postpartum period in Fujian Province, China: a qualitative study. *BMC Pregnancy and Childbirth* 2007;7(1):8.
44. Duan Y, Yang Z, Lai J, et al. Exclusive breastfeeding rate and complementary feeding indicators in China: A national representative survey in 2013. *Nutrients* 2018;10(2):249.
45. Tang L, Binns CW, Lee AH, et al. Low prevalence of breastfeeding initiation within the first hour of life in a rural area of Sichuan Province, China. *Birth* 2013;40(2):134-42.
46. China NHaFPCotPsRo. Order No. 17: National health and family planning Commission's decree on deciding to repeal the regulations on the sale of substitutes for breast milk substitutes. 2017

For peer review only

For peer review only - http://bmjopen.bmj.com/site/about/guidelines.xhtml

Table 1 Demographic and social-economic characteristics of the participants

	Maternal Education			
	Primary school and below (N=781)	Middle School (N=3842)	High School/Vocational School (N=1990)	College and above (N=3795)
Mean maternal age, year (SD)	28.76(6.68)	28.41(5.21)	28.26(4.78)	30.36(4.51)
Mean pre-pregnancy BMI, Kg/m^2 (SD)	22.67(4.66)	22.44(4.16)	21.74(4.04)	21.75(4.12)
Mean gestational age, week (SD)	39.02(1.38)	39.03(1.40)	38.94(1.51)	38.98(1.38)
Mean infant birth weight, kg (SD)	3.23(0.47)	3.33(0.59)	3.34(0.67)	3.43(0.75)
Mean infant age, day (SD)	186.50(106.82)	182.11(104.48)	178.29(105.82)	173.10(105.52)
Infant sex, %				
Male	50.67	50.64	52.17	50.08
Female	49.33	49.36	47.83	49.92
Parity, %				
Primiparous	29.70	33.87	43.49	62.06
Multiparous	70.30	66.13	56.51	37.94
Delivery method, %				
Vaginal	65.77	56.72	54.59	57.01
Cesarean section	34.23	43.28	45.41	42.99
Region, %				
Super city	9.70	14.16	30.82	61.55
Urban	43.15	32.08	39.09	27.41
Rural	7.15	25.39	21.09	7.29
Poor rural	40.00	28.37	9.00	3.75
Household income, %				
≤40,000 yuan	61.74	37.44	20.06	11.81
40,000-79,999 yuan	22.39	30.01	26.21	13.82
≥80,000 yuan	3.48	7.84	16.40	38.89
Maternal Occupation, %				
Agriculture related	57.89	33.61	9.37	2.27
Factory workers	11.53	19.95	20.31	8.66
White-collar	11.77	26.74	47.23	73.03
Others[1]	18.81	19.71	23.09	16.04
Resident status, %				
Local	69.70	69.15	59.50	53.65
Migrant	30.30	30.85	40.50	46.35
Breastfeeding practice, %				
Early initiation	64.85	68.24	71.11	77.21
Current BF	87.88	88.53	85.09	86.21

[1] Others refer to unidentified occupation, unemployed, and housewives

For peer review only - http://bmjopen.bmj.com/site/about/guidelines.xhtml

For peer review only

Ever BF	95.52	97.31	97.65	97.93
Exclusive BF (0-6 months)	26.53	15.36	13.64	14.06
Predominant BF (0-6 months)	50.00	39.99	18.44	39.29
Health Seeking Behaviors				
Considered feeding method before pregnancy	28.31	45.35	50.24	55.29
Attended mother support group	8.24	24.40	38.20	49.36
Received breastfeeding education	38.59	47.23	59.13	64.23

	Unadjusted Risk Ratio (95% CI)			
	Primary school and below	Middle School	High School/Vocational School	College and above
Early initiation	1	1.05(0.97,1.13)	1.10(1.01,1.19)	1.19(1.09,1.29)
Current BF	1	1.01(1.01,1.01)	0.97(0.96,1.00)	0.98(0.97,1.01)
Ever BF	1	1.02(1.02,1.03)	1.02(1.02,1.02)	1.03(1.03,1.03)
Exclusive BF (0-6 months)	1	0.58(0.52,0.64)	0.51(0.42,0.60)	0.53(0.47,0.59)
Predominant BF (0-6 months)	1	0.80(0.72,0.87)	0.37(0.24,0.50)	0.79(0.70,0.89)
	Adjusted Risk Ratio (95% CI)			
	Primary school and below	Middle School	High School/Vocational School	College and above
Early initiation	1	1.05(1.01,1.11)	1.10(1.03,1.17)	1.16(1.10,1.23)
Current BF	1	1.01(0.99,1.04)	1.02(0.99,1.05)	1.04(1.01,1.07)
Ever BF	1	1.01(1.01,1.01)	1.01(1.01,1.01)	1.01(1.01,1.01)
Exclusive BF (0-6 months)	1	0.66(0.53,0.81)	0.63(0.49,0.81)	0.74(0.65,0.83)
Predominant BF (0-6 months)	1	0.89(0.79,1.00)	0.84(0.72,0.97)	0.73(0.62,0.85)

Table 2. Relations between breastfeeding practices and maternal education*

**Adjusted for maternal age, infant birth weight, infant sex, parity, delivery method, household income, region (super city/urban/rural/poor rural), residency (local/migrant), paternal education, and occupation.*

For peer review only

For peer review only - http://bmjopen.bmj.com/site/about/guidelines.xhtml

Figure 1 Adjusted Prevalence* of early initiation in different maternal education groups, stratified by household Income

**After adjusting for maternal age, infant sex, parity, delivery method, paternal education, region (super city/urban/rural/poor rural), residency (local/migrant), occupation*

Figure 2 Adjusted Prevalence* of exclusive breastfeeding in different maternal education groups, stratified by household Income

*After adjusting for maternal age, infant sex, parity, delivery method, paternal education, region (super city/urban/rural/poor rural), residency (local/migrant), occupation

Figure 3 Adjusted Prevalence* of considering feeding method before pregnancy, attending mother support group, receiving breastfeeding education in different maternal education groups*

**After adjusting for maternal age, infant sex, parity, delivery method, paternal education, household income, region (super city/urban/rural/poor rural), residency (local/migrant), occupation.*

For peer review only - http://bmjopen.bmj.com/site/about/guidelines.xhtml
For peer review only

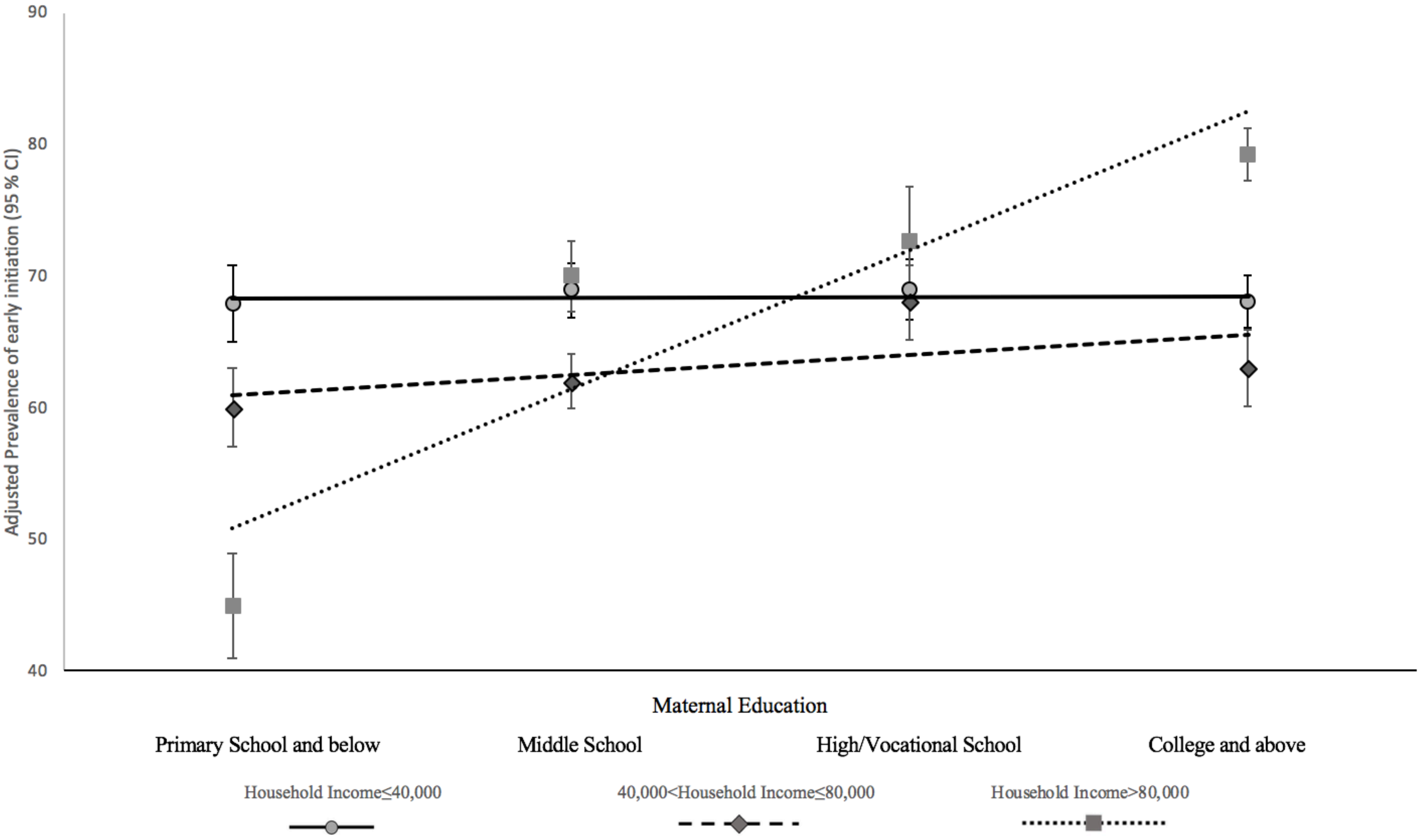
90
80
70
60
50
40
Adjusted Prevalence of early initiation (95 % CI)
Maternal Education
Primary School and below
Middle School
High/Vocational School
College and above
Household Income≤40,000
40,000<Household Income≤80,000
Household Income>80,000

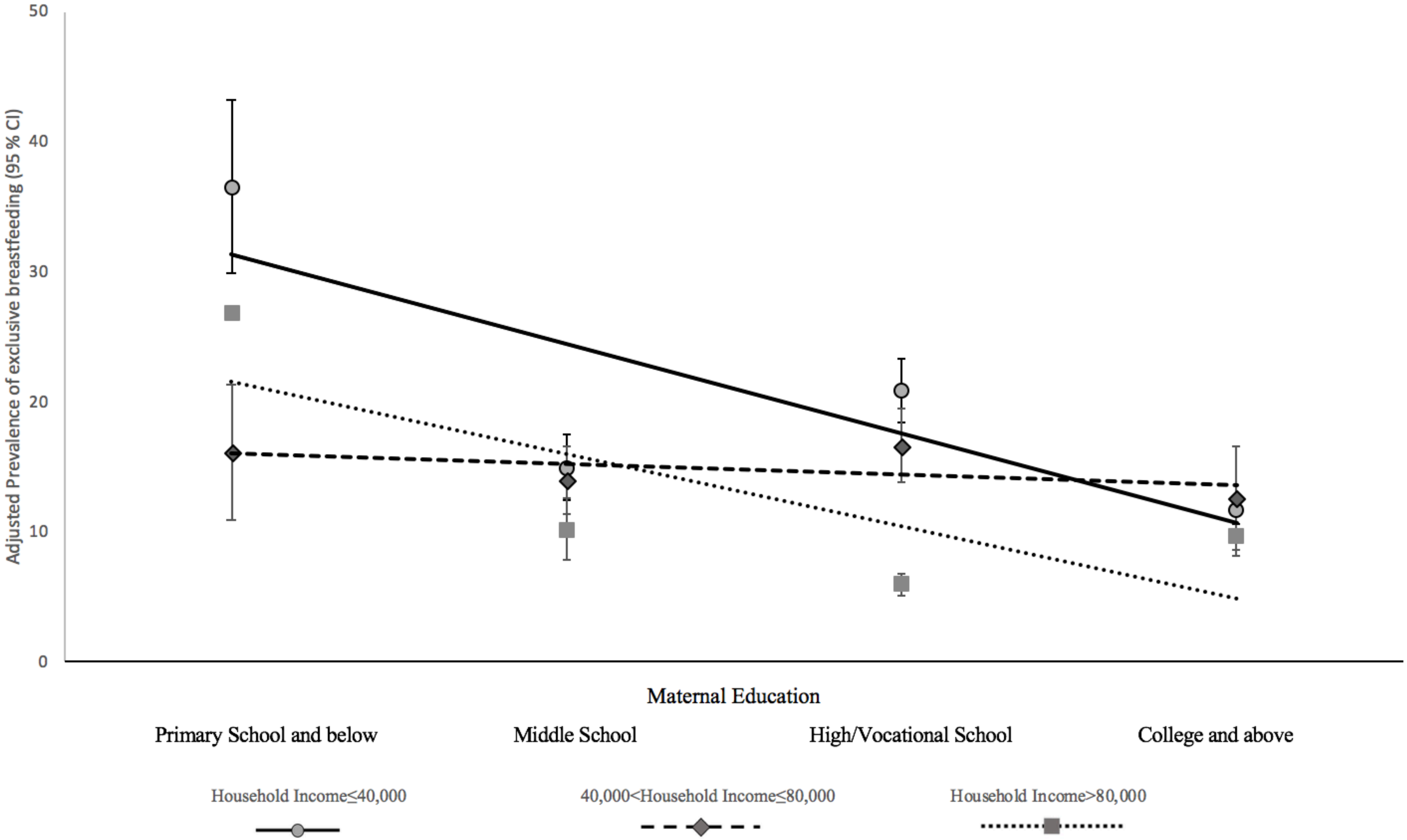
50
40
30
20
10
0
Adjusted Prevalence of exclusive breastfeeding (95 % CI)
Maternal Education
Primary School and below
Middle School
High/Vocational School
College and above
Household Income≤40,000
40,000<Household Income≤80,000
Household Income>80,000

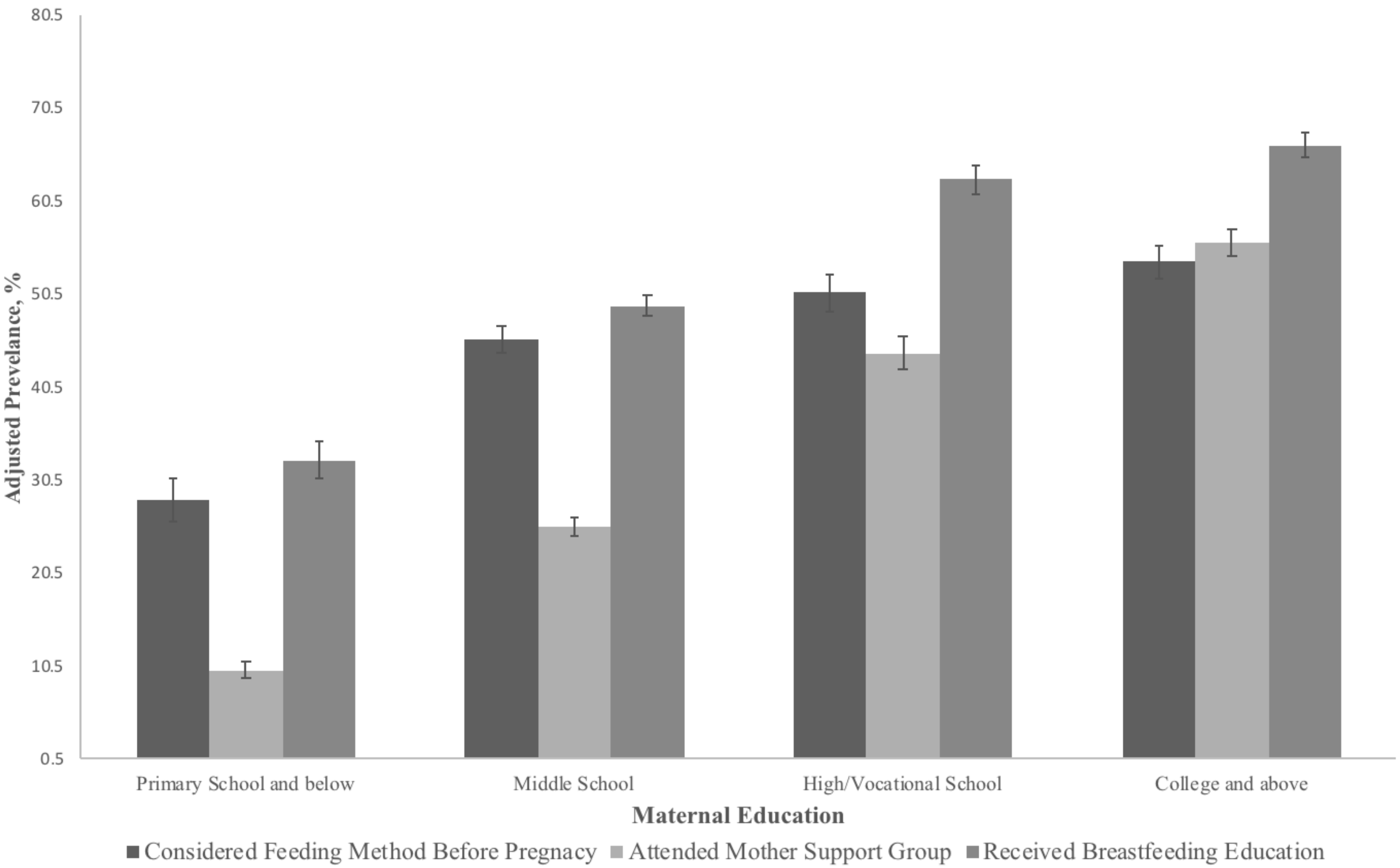
80.5
70.5
60.5
50.5
40.5
30.5
20.5
10.5
0.5
Adjusted Prevelance, %
Primary School and below
Middle School
High/Vocational School
College and above
Maternal Education
Considered Feeding Method Before Pregnacy
Attended Mother Support Group
Received Breastfeeding Education

Supplementary 1. Infant age at interview by maternal education.

	Maternal Education			
	Primary school and below (N=781)	Middle School (N=3842)	High School/Vocational School (N=1990)	College and above (N=3795)
Mean infant age, day (SD)	186.50(106.82)	182.11(104.48)	178.29(105.82)	173.10(105.52)
Range of infant age, day	0-364	0-364	0-364	0-364
Median	190.00	183.00	181.00	170.00
Distribution of infant age, %				
0~91 days (>0, ≤3 months)	23.30	24.83	25.83	27.27
92~183 days (>3, ≤6 months)	24.46	25.25	25.08	26.03
184~274 days (>6, ≤9 months)	24.33	25.43	26.13	23.90
275~364 days (>9, ≤12 months)	27.91	24.49	22.96	22.79

For peer review only

Reporting checklist for cross sectional study.

Based on the STROBE cross sectional guidelines.

Instructions to authors

Complete this checklist by entering the page numbers from your manuscript where readers will find each of the items listed below.

Your article may not currently address all the items on the checklist. Please modify your text to include the missing information. If you are certain that an item does not apply, please write "n/a" and provide a short explanation.

Upload your completed checklist as an extra file when you submit to a journal.

In your methods section, say that you used the STROBE cross sectional reporting guidelines, and cite them as:

von Elm E, Altman DG, Egger M, Pocock SJ, Gotzsche PC, Vandenbroucke JP. The Strengthening the Reporting of Observational Studies in Epidemiology (STROBE) Statement: guidelines for reporting observational studies.

		Reporting Item	Page Number
Title	#1a	Indicate the study's design with a commonly used term in the title or the abstract	1
Abstract	#1b	Provide in the abstract an informative and balanced summary of what was done and what was found	2-3
Background / rationale	#2	Explain the scientific background and rationale for the investigation being reported	4
Objectives	#3	State specific objectives, including any prespecified hypotheses	5
Study design	#4	Present key elements of study design early in the paper	5
Setting	#5	Describe the setting, locations, and relevant dates, including periods of recruitment, exposure, follow-up, and data collection	5-6
Eligibility criteria	#6a	Give the eligibility criteria, and the sources and methods of selection of participants.	5-6

For peer review only

	#7	Clearly define all outcomes, exposures, predictors, potential confounders, and effect modifiers. Give diagnostic criteria, if applicable	6-7
Data sources / measurement	#8	For each variable of interest give sources of data and details of methods of assessment (measurement). Describe comparability of assessment methods if there is more than one group. Give information separately for for exposed and unexposed groups if applicable.	6-8
Bias	#9	Describe any efforts to address potential sources of bias	6-8
Study size	#10	Explain how the study size was arrived at	5-6
Quantitative variables	#11	Explain how quantitative variables were handled in the analyses. If applicable, describe which groupings were chosen, and why	6-8
Statistical methods	#12a	Describe all statistical methods, including those used to control for confounding	8-9
	#12b	Describe any methods used to examine subgroups and interactions	8-9
	#12c	Explain how missing data were addressed	8-9
	#12d	If applicable, describe analytical methods taking account of sampling strategy	8-9
	#12e	Describe any sensitivity analyses	8-9
Participants	#13a	Report numbers of individuals at each stage of study—eg numbers potentially eligible, examined for eligibility, confirmed eligible, included in the study, completing follow-up, and analysed. Give information separately for for exposed and unexposed groups if applicable.	5-6
	#13b	Give reasons for non-participation at each stage	5-6
	#13c	Consider use of a flow diagram	5-6
Descriptive data	#14a	Give characteristics of study participants (eg demographic, clinical, social) and information on exposures and potential confounders. Give information separately for exposed and unexposed groups if applicable.	5-6
	#14b	Indicate number of participants with missing data for each variable of interest	5-6

For peer review only

Outcome data	#15	Report numbers of outcome events or summary measures. Give information separately for exposed and unexposed groups if applicable.	8
Main results	#16a	Give unadjusted estimates and, if applicable, confounder-adjusted estimates and their precision (eg, 95% confidence interval). Make clear which confounders were adjusted for and why they were included	9-11
	#16b	Report category boundaries when continuous variables were categorized	9-11
	#16c	If relevant, consider translating estimates of relative risk into absolute risk for a meaningful time period	9-11
Other analyses	#17	Report other analyses done—e.g., analyses of subgroups and interactions, and sensitivity analyses	9-10
Key results	#18	Summarise key results with reference to study objectives	12
Limitations	#19	Discuss limitations of the study, taking into account sources of potential bias or imprecision. Discuss both direction and magnitude of any potential bias.	12
Interpretation	#20	Give a cautious overall interpretation considering objectives, limitations, multiplicity of analyses, results from similar studies, and other relevant evidence.	13-15
Generalisability	#21	Discuss the generalisability (external validity) of the study results	13-15
Funding	#22	Give the source of funding and the role of the funders for the present study and, if applicable, for the original study on which the present article is based	17

The STROBE checklist is distributed under the terms of the Creative Commons Attribution License CC-BY. This checklist was completed on 10. December 2018 using http://www.goodreports.org/, a tool made by the EQUATOR Network in collaboration with Penelope.ai

For peer review only
